# Insights into olfactory ensheathing cell development from a laser-microdissection and transcriptome-profiling approach

**DOI:** 10.1002/glia.23870

**Published:** 2020-08-28

**Authors:** Surangi N. Perera, Ruth M. Williams, Rachel Lyne, Oliver Stubbs, Dennis P. Buehler, Tatjana Sauka-Spengler, Masaharu Noda, Gos Micklem, E. Michelle Southard-Smith, Clare V. H. Baker

**Affiliations:** 1 Department of Physiology, Development and Neuroscience, University of Cambridge, Cambridge, UK; 2 MRC Weatherall Institute of Molecular Medicine, Radcliffe Department of Medicine, University of Oxford, Oxford, UK; 3 Department of Genetics, University of Cambridge, Cambridge, UK; 4 Division of Genetic Medicine, Department of Medicine, Vanderbilt University School of Medicine, Nashville, Tennessee; 5 Division of Molecular Neurobiology, National Institute for Basic Biology, Okazaki, Japan

**Keywords:** boundary cap cells, neural crest, OECs, oligodendrocytes, Ptprz1, trigeminal Schwann cells, Wnt pathway

## Abstract

Olfactory ensheathing cells (OECs) are neural crest-derived glia that ensheath bundles of olfactory axons from their peripheral origins in the olfactory epithelium to their central targets in the olfactory bulb. We took an unbiased laser microdissection and differential RNA-seq approach, validated by in situ hybridization, to identify candidate molecular mechanisms underlying mouse OEC development and differences with the neural crest-derived Schwann cells developing on other peripheral nerves. We identified 25 novel markers for developing OECs in the olfactory mucosa and/or the olfactory nerve layer surrounding the olfactory bulb, of which 15 were OEC-specific (that is, not expressed by Schwann cells). One pan-OEC-specific gene, *Ptprz*1, encodes a receptor-like tyrosine phosphatase that blocks oligodendrocyte differentiation. Mutant analysis suggests Ptprz1 may also act as a brake on OEC differentiation, and that its loss disrupts olfactory axon targeting. Overall, our results provide new insights into OEC development and the diversification of neural crest-derived glia.

## 1 Introduction

Olfactory ensheathing cells (OECs) are neural crest-derived glia (Barraud et al., 2010; Forni, Taylor-Burds, Melvin, Williams, & Wray, 2011) that ensheath bundles of axons extending from olfactory receptor neurons in the olfactory epithelium (which are continuously regenerated throughout life) to their glomerular targets in the olfactory bulb of the rostral forebrain (reviewed by Ekberg, Amaya, Mackay-Sim, & St John, 2012; Radtke & Kocsis, 2014; Barton, St John, Clarke, Wright, & Ekberg, 2017). OECs have traditionally been considered unique amongst glia in being found in both the peripheral and central nervous system (CNS) and forming the glia limitans of the olfactory system (Doucette, 1991). Recently, the glia limitans was reported to be formed by astrocytes, just as elsewhere in the CNS, suggesting that all OECs are peripheral glia, including those within the olfactory nerve layer (ONL) of the olfactory bulb (Nazareth et al., 2019). However, juxtaglomerular OECs (in the innermost ONL) are coupled to astrocytes by gap junctions (Beiersdorfer, Scheller, Kirchhoff, & Lohr, 2019) and although astrocytes form the blood-brain barrier in the glomerular layer and the inner ONL, OECs in the outer ONL contribute to the blood-brain barrier (Beiersdorfer et al., 2020). Overall, OECs seem to exhibit properties of both CNS and peripheral glia and form part of the transitional zone between the peripheral and central nervous systems (Beiersdorfer et al., 2020).

OECs cultured from either the olfactory mucosa (accessible in the nasal cavity) or the olfactory bulb have shown promise in cell-mediated repair of CNS lesions in animal models: they migrate freely in the CNS environment, promote axon sprouting, remyelinate axons, and phagocytose debris (reviewed by Ekberg & St John, 2014; Radtke & Kocsis, 2014; Roet & Verhaagen, 2014; Assinck, Duncan, Hilton, Plemel, & Tetzlaff, 2017; Yao et al., 2018). Schwann cells (the neural crest-derived glia of all other peripheral nerves; Jacob, 2015; Jessen, Mirsky, & Lloyd, 2015) have also shown potential in cell-mediated repair of CNS lesions (reviewed by Bunge, 2016; Assinck et al., 2017). However, they induce astrocytes to undergo hypertrophy and produce axon growth-inhibiting chondroitin sulfate proteoglycans (Lakatos, Barnett, & Franklin, 2003; Lakatos, Franklin, & Barnett, 2000), and CNS myelin inhibits Schwann cell migration and promotes Schwann cell death (Chaudhry et al., 2017). The contamination of mucosal OEC cultures with Schwann cells (from trigeminal nerve branches) is problematic but there are no reliable antigenic markers to distinguish OECs and Schwann cells in culture (Ekberg & St John, 2014). An understanding of the molecular mechanisms underlying neural crest differentiation into OECs, as opposed to Schwann cells (whose development has been much more extensively studied; Jacob, 2015; Jessen et al., 2015), could therefore provide insights into how to optimize the use of OECs in clinical contexts (Ekberg & St John, 2014; Yao et al., 2018), as well as the diversification of neural crest-derived glia (Jacob, 2015).

Moreover, OECs are not a homogeneous population (reviewed by Ekberg et al., 2012; Ekberg & St John, 2014; Yao et al., 2018). The thick rostral and ventral regions of the ONL (where olfactory axons enter the ONL, after traversing the cribriform plate) have distinct inner and outer layers (Au, Treloar, & Greer, 2002; Treloar, Feinstein, Mombaerts, & Greer, 2002). In the olfactory mucosa, OECs ensheath bundles of heterotypic olfactory axons (i.e., expressing different olfactory receptors) projecting from the olfactory epithelium; in the outer ONL these axons are defasciculated, then extend into the inner ONL where they are fasciculated into homotypic bundles projecting to glomeruli (Ekberg et al., 2012; Mombaerts et al., 1996; Treloar et al., 2002). Dorsal and caudal regions of the ONL, in contrast to the rostral and ventral ONL, are relatively thin, primarily containing sorted axons projecting to their target glomeruli (St John & Key, 2001). A few molecular differences have been identified in vivo between OECs in different regions of the olfactory system. In the mouse, inner ONL-OECs express neuropeptide tyrosine (Npy; Au et al., 2002; Barraud, St John, Stolt, Wegner, & Baker, 2013), the transcription factor gene *Runx*1 (Murthy, Bocking, Verginelli, & Stifani, 2014), and the serine protease gene *Prss56* (Jourdon et al., 2016), and are enriched (over outer ONL-OECs and mucosal OECs) for the secreted Wnt inhibitor gene *Frzb* (Rich et al., 2018). Conversely, outer ONL-OECs and mucosal OECs were reported to express the low-affinity neurotrophin receptor p75^NTR^ (Au et al., 2002; Au et al., 2002; Franceschini & Barnett, 1996), while a subset of OECs at the outer edge of the ONL expresses the secreted semaphorin gene *Sema3a* (Schwarting et al., 2000).

In addition to these molecular differences in vivo, olfactory axons cocultured with mucosal OECs are less dispersed than those cocultured with rostral ONL-OECs containing a mixture of inner and outer ONL-OECs (Windus et al., 2010). Furthermore, mucosal OECs mostly adhere to other mucosal OECs in culture, while ONL-OECs are more heterogeneous in their response to other ONL-OECs, showing contact-adhesion, contact-repulsion, and “cross-over,” that is, exploration without either adhesion or repulsion (Windus et al., 2010). Physiological differences have also been identified between different OEC subpopulations. Outer but not inner ONL-OECs show an increase in intracellular calcium in response to a variety of agonists (Thyssen et al., 2013). Conversely, inner but not outer ONL-OECs propagate Ca^2+^ waves via gap junctions and display inward rectifier (K_ir_) potassium currents (Rela, Bordey, & Greer, 2010; Rela, Piantanida, Bordey, & Greer, 2015; Stavermann et al., 2015).

Taken together, these molecular and phenotypic differences between different OEC subpopulations in vivo and in vitro provide indirect support for the hypothesis that mucosal OECs help fasciculate olfactory axons into heterotypic bundles, whereas outer ONL-OECs defasciculate them, and inner ONL-OECs help to sort and refasciculate homotypic axons (Ekberg et al., 2012; Ekberg & St John, 2014). Indeed, olfactory axon targeting is disrupted in mouse embryos lacking *Frzb*, which is enriched in inner ONL-OECs over outer ONL-OECs and mucosal OECs (Rich et al., 2018). Deletion of the transcription factor gene *Sox10,* the earliest known marker for developing OECs and maintained throughout mucosal and ONL-OEC development (Barraud et al., 2010; Barraud et al., 2013; Forni et al., 2011), disrupts OEC differentiation and, in turn, olfactory axon targeting (Barraud et al., 2013; Pingault et al., 2013). Sox10 deletion (but not *Frzb* deletion; Rich et al., 2018) also significantly reduces the proportion of gonadotropin-releasing hormone (GnRH) neurons that enter the embryonic forebrain (Barraud et al., 2013; Pingault et al., 2013). GnRH neurons are surrounded by OECs as they migrate along olfactory, vomeronasal and terminal nerve axons to the forebrain (Geller et al., 2017; Geller, Kolasa, Tillet, Duittoz, & Vaudin, 2013; Taroc, Prasad, Lin, & Forni, 2017), where they are required in the adult hypothalamus for fertility (Cariboni, Maggi, & Parnavelas, 2007; Forni & Wray, 2015).

What molecular differences between OEC subpopulations (mucosal OECs, outer ONL-OECs, inner ONL-OECs) might underlie the postulated differences in their interaction with olfactory axons (Ekberg et al., 2012; Ekberg & St John, 2014), and also perhaps their interactions with migrating GnRH neurons? How are such differences established during OEC development? To what extent do the molecular mechanisms controlling OEC development, and the formation of distinct OEC subpopulations, differ from those that underlie Schwann cell development? The transcriptional profiles of late-embryonic or adult mucosal OECs, ONL-OECs and/or Schwann cells have previously been compared using microarrays (Franssen, De Bree, Essing, Ramón-Cueto, & Verhaagen, 2008; Guérout et al., 2010; Pastrana et al., 2006; Roet, Bossers, Franssen, Ruitenberg, & Verhaagen, 2011; Ulrich et al., 2014; Vincent, Taylor, Choi-Lundberg, West, & Chuah, 2005). However, these studies focused on cells grown in culture, which has a significant impact on gene expression (see, e.g., Franssen et al., 2008; Ulrich et al., 2014).

Here, we aimed to shed light on the mechanisms underlying OEC development and diversification, and identify molecular differences between developing OEC subpopulations and Schwann cells in vivo. We took an unbiased differential RNA-seq approach, comparing the transcriptomes of embryonic olfactory and trigeminal nerve regions isolated in situ by laser microdissection, and validating candidate genes by in situ hybridization. Our results suggest that developing OECs and Schwann cells are already distinct from early stages of OEC development, and provide novel insight into the transcription factors and signalling pathways likely to be important for the development and function of OECs, including different OEC subpopulations. Furthermore, we identify *Ptprz*1, encoding a receptor-like tyrosine phosphatase whose activity blocks oligodendrocyte differentiation (Kuboyama et al., 2012; Kuboyama, Fujikawa, Suzuki, & Noda, 2015; Kuboyama, Fujikawa, Suzuki, Tanga, & Noda, 2016; Tanga et al., 2019), as a pan-OEC-specific gene (i.e., not expressed by trigeminal Schwann cells). Deletion of Ptprz1 seems to promote OEC differentiation, and disrupts olfactory axon targeting but not GnRH neuron entry into the forebrain.

## 2 Materials and Methods

### 2.1 Ethics statement

Experiments involving *Sox10:Histone2BVenus* mice (Corpening et al., 2011) were approved by the Vanderbilt University Institutional Animal Care and Use Committee (IACUC). Experiments involving the generation of *Ptprz1^lacZ^* and *Ptn* knockout embryos (Muramatsu et al., 2006; Shintani, Watanabe, Maeda, & Noda, 1998) were approved by the IACUC of the National Institutes of Natural Sciences, Japan.

### 2.2 Mouse embryos

All transgenic or mutant mouse embryos used were previously described: *Sox10:Histone2BVenus* (MGI strain ID 5544794; Corpening et al., 2011), *Ptprz1^lacZ^* (MGI strain ID 2678757; Shintani et al., 1998) and *Ptn* knockout (MGI strain ID 3053747; Muramatsu et al., 2006).

### 2.3 Laser microdissection and RNA extraction

Embryos were dissected in phosphate-buffered saline (PBS), embedded in optimal cutting temperature compound (OCT, Tissue Tek) and flash-frozen in isopentane on dry ice. Cryosections of 8 μm were collected on polyester (POL) membrane slides (Leica Microsystems, Wetzlar, Germany). The POL membrane slides with collected sections were dehydrated in a desiccation chamber with Drierite (with indicator) for 30-45 min as described (Morrison, Bailey, & Kulesa, 2012; Morrison, Box, McKinney, McLennan, & Kulesa, 2015). Laser microdissection (Frost, Eltoum, Siegal, Emmert-Buck, & Tangrea, 2015) was performed using a Leica LMD 600 in the Cambridge Advanced Imaging Centre (University of Cambridge, Cambridge, UK).

Laser-microdissected tissue from four to seven sections was pooled for each different nerve-region sample at each embryonic stage. Samples were collected from four different embryos at each stage except for E11.5 olfactory nerve (*n* = 2) and E16.5 trigeminal nerve (*n* = 3). RNA was extracted from microdissected tissue (collected in the lysis buffer) using the Arcturus PicoPure RNA isolation kit (Thermo Fisher Scientific, Waltham, MA) according to the manufacturer’s instructions. An in-column DNase treatment was performed after the RNA was bound to the column using the RNase-free DNase set (Qiagen, Hilden, Germany) according to the manufacturer’s instructions. The quality and quantity of the RNA was assessed using the RNA 6000 Pico Kit in the 2100 Bioanalyser (Agilent Technologies, Santa Clara, CA).

### 2.4 cDNA synthesis and amplification, library preparation, and sequencing

The SMART-seq V4 ultra low-input RNA kit for sequencing (Takara Bio, Kusatsu, Japan) was used for cDNA synthesis and amplification according to the manufacturer’s instructions using ~2,000 pg of RNA. Amplified cDNA was purified using Agencourt AMPure XP Beads (Beckman Coulter, Brea, CA) according to the manufacturer’s instructions. One nanogram of amplified cDNA was used for paired-end library preparation using the Nextera XT DNA library prep kit (Illumina, San Diego, CA) according to the manufacturer’s instructions. Amplified libraries were purified using Agencourt AMPure XP Beads according to the manufacturer’s instructions. The concentration of cDNA/libraries was checked using a Qubit (Thermo Fisher Scientific) and validated using the 2100 bioanalyser or Tapestation (Agilent Technologies).

Libraries were pooled (usually 12-13 libraries were pooled together) to a final concentration of 4 nM and quantified using the KAPA Library Quantification Kit (ABI Prism) from Kapa Biosystems (Wilmington, MA) according to the manufacturer’s instructions. After quantification, the pooled libraries were reconcentrated and loaded on a NextSeq flowcell (Illumina) for sequencing on a NextSeq 500 (Illumina).

### 2.5 Bioinformatics

Sequencing quality was assessed with FastQC (http://www.bioinformatics.babraham.ac.uk/projects/fastqc/) and aligned to the mouse reference genome mm10 (version grcm38.84 downloaded from Ensembl) using Tophat2 (Kim et al., 2013) version 2.1.0. Alignment files were sorted by name and a count matrix was produced using HTSeq (Anders, Pyl, & Huber, 2015). The fragments per kilobase of transcript per million mapped reads (FPKM) and differential expression analysis were generated using DESeq2 (Love, Huber, & Anders, 2014) version 3.7.

RNA-seq data have been deposited in the NCBI Gene Expression Omnibus (GEO) database under accession code GSE138596.

### 2.6 Riboprobes

Digoxigenin-labelled antisense riboprobes were generated from cDNA clones as described (Henrique et al., 1995). *Frzb* was a gift of Christine Hartmann (Westfälische Wilhelms-Universitat Munster, Munster, Germany). *Hes*1 was previously described (Ishibashi et al., 1995). *Hes5* was a gift of Bernd Fritzsch (University of Iowa, Iowa City, IA). *Mpz* (from rat; Morgan, Jessen, & Mirsky, 1994) was a gift from Kristjan Jessen and Rhona Mirsky (University College London, London, UK). *Npy* was previously described (Rich et al., 2018). *Wif*1 (Park et al., 2014) was a gift from Jose Teixeira (Michigan State University, Grand Rapids, MI). Clones used to generate riboprobes for some of the candidate genes validated as not being expressed in OECs or Schwann cells (File S3) were also gifts: *Ascl*1 (James Briscoe, Francis Crick Institute, London, UK), *Bmper, Dlxi,* and *Pou3f3* (Juhee Jeong, New York University College of Dentistry, New York, NY), *Cnpy1* (Jean Hebert, Albert Einstein College of Medicine, New York, NY), *Ebf2* (Alain Vincent, CNRS/Universite Toulouse III, France), *Epha3* and *Sall1* (Yasuhiko Kawakami, University of Minnesota, Minneapolis, MN), *Fezf1* (Masahiko Hibi, Nagoya University, Nagoya, Japan), *Neurod6* (Joshua Sanes, Harvard University, Boston, MA), *Neurog1* (François Guillemot, Francis Crick Institute, London, UK), *Nrn1* (Peter Oliver, University of Oxford, Oxford, UK), *Nts* (Takeshi Sakurai, University of Tsukuba, Tsukuba, Japan), *Osr2* and *Shox2* (Rulang Jiang, Cincinnati Children’s Hospital Medical Center, Cincinnati, OH), *Sfrp2, Wnt7a* and *Wnt7b* (Vassiliki Fotaki, University of Edinburgh, Edinburgh, UK), *Six1* (Isabelle Chang, Max Planck Research Unit for Neurogenetics, Frankfurt, Germany), *Tbx18* (Andreas Kispert, Hannover Medical School, Hannover, Germany), *Zic1* (Kate Barald, University of Michigan Medical School, Ann Arbor, MI).

For all other genes, a 400-1,000-bp fragment of cDNA (File S4) was amplified by polymerase chain reaction (PCR) from single-strand cDNA (prepared using Thermo Fisher Scientific’s high-capacity cDNA Reverse Transcription Kit on total RNA extracted with Trizol [Invitrogen] from E13.5 mouse embryo heads). Each cDNA fragment was cloned into pDrive (Qiagen) using the Qiagen PCR cloning kit and sequenced (Biochemistry Department DNA Sequencing Facility, University of Cambridge, Cambridge, UK). Primer-BLAST software from NCBI (Ye et al., 2012) was used to design PCR primers and check their specificity. Primer melting temperature and self-complementarity were checked using Primer3Plus (Untergasser et al., 2012).

### 2.7 Cryosectioning and in situ hybridization

Embryos were fixed in 4% paraformaldehyde in PBS overnight at 4°C, cryoprotected in 30% sucrose and embedded in OCT (Tissue Tek, Sakura Finetek, Torrance, CA) for cryosectioning at 10 μm. In situ hybridization (ISH) was performed as described (Miller, Perera, & Baker, 2017) on cryosections from at least two different embryos. ISH data are only reported here if results were consistent across different embryos. All *n* numbers given for ISH data refer to embryos. ISH is not quantitative: slides from wild-type and mutant mouse embryos were only compared directly when ISH was performed in the same round, that is, all slides were treated identically, including the development of the colour reaction and image processing.

### 2.8 Immunohistochemistry

Immunohistochemistry was performed as described (Rich et al., 2018). Primary antibodies were used against olfactory marker protein (Omp; goat, Wako 019-22291, Research Resource Identifier [RRID]: AB_664696; 1:500), Sox10 (rabbit, gift of Vivian Lee, Medical College of Wisconsin, WI, 1:3,000; Meng, Yuan, & Lee, 2011; Yardley & Garcia-Castro, 2012), Tbr1 (rabbit, Abcam ab31940, RRID: AB_2200219; 1:1,000; expression data summarized in File S3) and Tubb3 (neuronal αIII tubulin; mouse IgG2a, clone TUJ1, Covance MMS-435P, RRID:AB_2313773; 1:250). Matched AlexaFluor-conjugated secondary antibodies (Molecular Probes, Thermo Fisher Scientific) were used at 1:1,000. For triple immunostaining, anti-Tubb3 was detected by a biotinylated secondary antibody (goat antimouse IgG2a, Invitrogen, 1:100), followed by Alexa350-conjugated NeutrAvidin (Molecular Probes, 1:100). Slides were mounted with Fluoromount G (Southern Biotech, Birmingham, AL) or Vectashield with 4’,6-diamidino-2’-phenylindole dihydrochloride (DAPI; Vector Labs, Burlingame, CA).

### 2.9 Image capture and processing

Images were captured using a Zeiss Axioskop 2 microscope (Carl Zeiss, Oberkochen, Germany) equipped with a QImaging Retiga 2000R camera and an RGB pancake (Qimaging, Surrey, BC, Canada), using QCapture Pro 6.0 software (Qimaging). Images were processed in Adobe Photoshop CS6 (Adobe Systems Inc., San Jose, CA).

### 2.10 Analysis of olfactory receptor neuron maturation and olfactory epithelium thickness

Immunostaining for the olfactory receptor neuron maturation marker Omp (Farbman & Margolis, 1980; Monti Graziadei, Stanley, & Graziadei, 1980) and the general axonal/neuronal marker Tubb3, plus counterstaining for DAPI, were performed on 10-μm serial coronal sections spanning the rostrocaudal extent of the olfactory bulb at E14.5-E16.5 (10 slides per series: on each slide, each section was collected every 100 μm). After imaging, Adobe Photoshop CS6 was used to place a 200-μm bar in the Tubb3-channel image (to reduce bias) along the dorsal olfactory epithelium on left and right sides of the nasal septum (i.e., two measurements per section), for 4-6 sections per embryo (i.e., 8-12 measurements per embryo). Within this 200-μm region of olfactory epithelium, all Tubb3-positive and DAPI-positive cells (i.e., neurons) and all triple Omp-positive, Tubb3-positive, DAPI-positive cells (i.e., mature neurons) were counted, and the thickness of the epithelium measured at three points (at each edge of the bar, and at a third point near the centre of the bar).

### 2.11 Analysis of GnRH neuron numbers

GnRH neurons (identified by in situ hybridization for *Gnrh1)* were counted on 10-μm coronal sections through the olfactory system and forebrain (10 slides per series: on each slide, each section was collected every 100 μm) at E16.5.

### 2.12 Statistical analysis

Microsoft Excel was used for initial data analysis. GraphPad Prism 7 (GraphPad Software, La Jolla, CA) was used for all statistical analyses and to generate scatter plots showing mean and SD. For data sets passing the Shapiro-Wilk normality test (*n* ≥ 3; *p* > .05), means of two groups were compared using an unpaired two-tailed Student’s *t* test, and means of three groups (i.e., wild-type, heterozygous, and homozygous littermates) were compared using one-way analysis of variance with Dunnett’s multiple comparison test (comparing the mean for each mutant genotype with the wild-type mean). For data sets where *p* < .05 for the Shapiro-Wilk normality test, three groups were compared using the Kruskal-Wallis test followed by Dunn’s multiple comparisons test.

## 3 Results

### 3.1 Laser microdissection of embryonic nerves for transcriptome profiling

**Figure 1:**
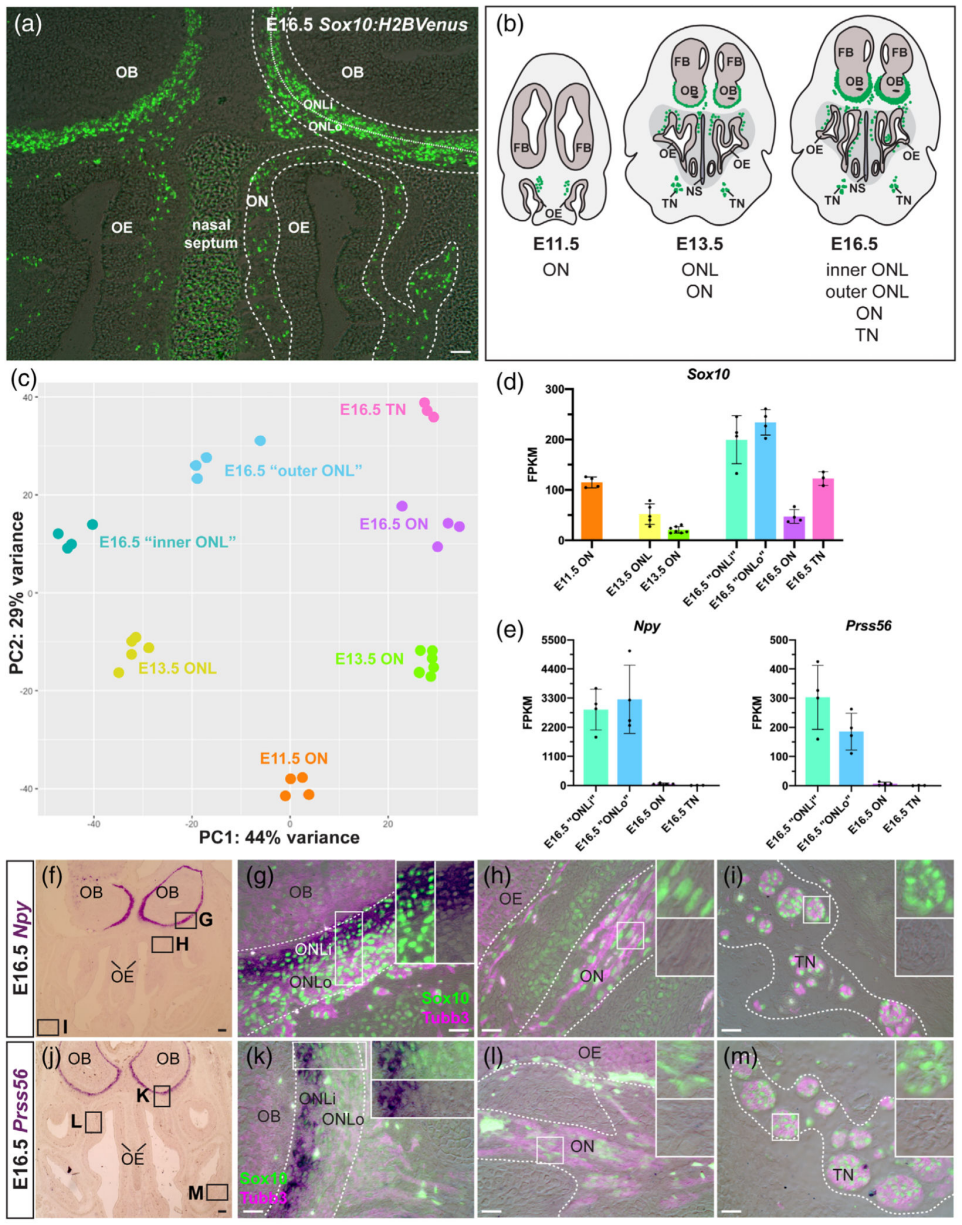
Laser microdissection and transcriptomic profiling of embryonic nerves identify known differences between OEC subpopulations and trigeminal Schwann cells. (a) Frontal section through the olfactory system of an E16.5 *Sox10:H2BVenus* BAC transgenic mouse embryo (native Venus fluorescence overlaid on bright-field image) showing Venus-expressing OECs associated with olfactory nerve fascicles adjacent to the olfactory epithelium (i.e., in the lamina propria of the olfactory mucosa) and throughout the ONL, which comprises an outer ONL (ONLo) and inner ONL (ONLi) at this stage. (Trigeminal nerve branches are present on this section but cannot be seen in the image.) (b) Schematic coronal sections through the olfactory system showing the location of OECs and Schwann cells (green). The nerve regions laser-microdissected at each stage are indicated below each schematic. (c) PC analysis of all transcriptomes reveals clustering of replicates (E11.5 olfactory nerve, *n* =4 from two embryos; E13.5 olfactory nerve, *n* = 7 from four embryos; E13.5 ONL, *n* = 5 from four embryos; E16.5 olfactory nerve and ONL, *n* =4 from four embryos for all samples; E16.5 trigeminal nerve, *n* = 3 from three embryos), and separation of transcriptomes by nerve region (PC1, accounting for 44% of the variance) and embryonic stage (PC2, accounting for 29% of the variance). (d) Bar chart showing mean expression values (FPKM) for *Sox10* across all transcriptomes at all stages. Error bars indicate SD. (e) Bar charts showing mean expression values for *Npy* and *Prss56* across all transcriptomes at E16.5. (f-m) Coronal sections through the mouse olfactory system at E16.5, with immunofluorescence images overlaid on the bright-field image. Sections were immunostained for Sox10 (green nuclei) to identify OECs and Schwann cells and for Tubb3 (magenta) to identify axons, following in situ hybridization for: (f-i) *Npy,* which is strongly expressed by inner ONL-OECs and much more weakly expressed by outer ONL-OECs, but not by mucosal OECs or trigeminal Schwann cells (*n* > 10); (j-m) *Prss56,* which is restricted to inner ONL-OECs, with no expression in outer ONL-OECs, mucosal OECs or trigeminal Schwann cells (*n* > 10). Scale bars: (a) 50 μm; (f,j) 100 μm; (g-i,k-m) 25 μm. FB, forebrain; FPKM, fragments per kilobase of transcript per million mapped reads; OB, olfactory bulb; OE, olfactory epithelium; OEC, olfactory ensheathing cell; ON, olfactory nerve; ONL, olfactory nerve layer; ONLi, inner olfactory nerve layer; ONLo, outer olfactory nerve layer; PC, principal component; TN, trigeminal nerve [Color figure can be viewed at wileyonlinelibrary.com]

Our overall aim was to use comparative transcriptome profiling to gain insight into how the development of OECs, or OEC subpopulations, might differ from the development of Schwann cells, and to identify molecular markers that could be used to distinguish these neural crest-derived glial cells. To achieve this, we took advantage of the fact that OECs and Schwann cells express *Sox10* throughout their development (Barraud et al., 2010; Barraud et al., 2013; Forni et al., 2011; Jessen et al., 2015) and can be identified on cryosections of *Sox10:H2BVenus* BAC transgenic mouse embryos (Corpening et al., 2011) by native fluorescence, without any need for immunostaining (Figure 1a). This facilitated laser microdissection (under epifluorescence) of pieces of olfactory and trigeminal nerve (the latter from trigeminal nerve branches adjacent to the olfactory system) from cryosections at different embryonic stages (Figure 1b).

At E11.5, pieces of olfactory nerve were laser-microdissected from the lamina propria of the olfactory mucosa (Figure 1b; *n* = 4 from two embryos). This stage is only 12 hours after OECs are first detectable by immunostaining for the early glial marker fatty acid-binding protein 7 (Fabp7, also known as brain lipid-binding protein, Blbp) in the “migratory mass” of olfactory axons and migrating neurons (including GnRH neurons) emerging from the olfactory epithelium (Miller, Treloar, & Greer, 2010). At this stage, the migratory mass has extended along the rostromedial surface of the telencephalon and olfactory axons have contacted its rostral tip, but remain outside (Doucette, 1989; Marin-Padilla & Amieva, 1989; Treloar, Miller, Ray, & Greer, 2010).

At E13.5, pieces of olfactory nerve were laser-microdissected from both the lamina propria of the olfactory mucosa (*n* = 7 from four embryos) and the ONL (Figure 1b; *n* = 5 from four embryos). This is roughly a day after the olfactory bulb is first morphologically distinct as an evagination from the telencephalon, and when the developing ONL around the olfactory bulb is starting to thicken (Doucette, 1989; Marin-Padilla & Amieva, 1989; Treloar et al., 2010).

At E16.5, pieces of olfactory nerve were laser-microdissected from the lamina propria of the olfactory mucosa (*n* = 4 from four embryos), the “inner ONL” (ONLi; closest to the olfactory bulb; *n* =4 from four embryos; Figure 1a,b) and the “outer ONL” (ONLo; furthest from the olfactory bulb; *n* = 4 from four embryos; Figure 1a,b). (Quotation marks are used for “inner” versus “outer” ONL tissue because these laser-microdissections were based on the anatomical location of Sox10^+^ cells closest to or furthest from the olfactory bulb, respectively, and ultimately proved not to separate the molecularly distinct inner ONL-OEC and outer ONL-OEC subpopulations; see next section.) Pieces of trigeminal nerve were also laser-microdissected from branches near the olfactory system on the same sections (Figures 1b and S1; *n* = 3 from three embryos).

Total RNA was extracted from the laser-microdissected nerve pieces (Figure S1) for cDNA library synthesis and next-generation sequencing, to generate nerve transcriptomes expected to include OEC subpopulations (i.e., mucosal OECs, ONL-OECs) or Schwann cells at different developmental stages. Cross-wise differential expression analysis between the various nerve transcriptomes using DESeq2 (Love et al., 2014) identified thousands of differentially expressed genes (Files S1 and S2). Principal component (PC) analysis of all transcriptomes showed that the replicates for each sample clustered together (Figure 1c). PC1 separated the samples by nerve region, while PC2 separated them by embryonic stage (Figure 1c).

### 3.2 In situ hybridization validates the use of differential RNA-seq on microdissected nerve pieces to identify differences between OEC subpopulations and Schwann cells

All transcriptomes included *Sox10* (Figure 1d), suggesting that OECs and Schwann cells were represented, as expected (Barraud et al., 2010; Barraud et al., 2013; Forni et al., 2011; Jessen et al., 2015). (This did not of course mean that the laser-microdissected tissues only contained glial cells, but showed that they included the target populations.) Furthermore, *Npy,* which is specific to inner ONL-OECs by immunoreactivity at E16.5 (Barraud et al., 2013) as well as in adult mice (Au et al., 2002), was highly enriched in both “inner ONL” and “outer ONL” versus the olfactory and trigeminal nerve transcriptomes at E16.5 (Figure 1e). Finally, *Prss56* (encoding serine protease 56), which was recently reported to be specifically expressed by inner ONL-OECs in adult mice, and by OECs in the ONL but not olfactory nerve at E13.5 (Jourdon et al., 2016), was highly enriched at E16.5 in both “inner ONL” and “outer ONL” versus olfactory nerve transcriptomes, and essentially undetectable in the trigeminal nerve (Figure 1e).

The differences in expression levels of *Npy* and *Prss56* at E16.5 in the ONL, olfactory nerve and trigeminal nerve transcriptomes (as shown in the bar charts in Figure 1e) were validated using ISH on cryosections, followed by immunohistochemistry for Sox10 to identify OECs (Barraud et al., 2010; Barraud et al., 2013; Forni et al., 2011) and Schwann cells (Jessen et al., 2015), and for neuronal beta-III tubulin (Tubb3) to identify axons. All ISH data are presented with a low-power view for orientation, followed by higher power views of the nerve regions used for the RNA-seq analyses shown in the bar charts, that is, the ONL, olfactory nerve fascicles near the olfactory epithelium, and the trigeminal nerve. (Figure S1a-d shows this in schematic form.) The ISH analysis showed that *Npy* (Figure 1f-i) was highly enriched in inner ONL-OECs, with only very weak expression in outer ONL-OECs (Figure 1g) and no obvious expression in either mucosal OECs (Figure 1h) or trigeminal Schwann cells on the same section (Figure 1i). As previously reported for adult mice (Jourdon et al., 2016), expression of *Prss56* at E16.5 (Figure 1j-m) was restricted to inner ONL-OECs (Figure 1k), with no detectable expression in either outer ONL-OECs (Figure 1k), mucosal OECs (Figure 1l) or trigeminal Schwann cells (Figure 1m).

Taken together, these data show that known molecular differences between ONL-OECs and mucosal OECs, and between OECs and Schwann cells, are reflected in the transcriptomes of micro-dissected ONL and olfactory nerve from the olfactory mucosa, and of trigeminal nerve, validating this approach to identify novel molecular differences between ONL-OECs, mucosal OECs, and Schwann cells. However, the “inner ONL” and “outer ONL” transcriptomes do not accurately reflect molecular differences between inner ONL-OECs and outer ONL-OECs, likely because there was no morphological boundary to guide microdissection.

We used ISH to validate the expression of multiple candidate genes identified from the cross-wise transcriptome comparisons at E16.5, focusing on genes encoding transcription factors, transmembrane receptors, and secreted proteins. This identified 30 genes expressed by OECs, of which 25 were novel for OECs. Most were differentially expressed between OECs (either all or specifically ONL-OECs) and Schwann cells, or between ONL-OECs and mucosal OECs (and Schwann cells). However, the ISH validation screen also showed that the laser-microdissected nerve pieces included some adjacent tissue, as many candidate genes proved to be expressed not by glia, but in tissues immediately adjacent to the laser-microdissected nerve regions. Some genes, for example, *Tfap2b, Dlk1* and *Cavin1,* showed very specific expression patterns in mesenchyme surrounding the ONL and/or olfactory or trigeminal nerve fascicles (Figure S2 and File S3), while others, such as *Hes6* and *Calb2,* were expressed in the olfactory bulb subjacent to the ONL and/or the olfactory epithelium (Figure S3 and File S3). This showed the importance of validating by ISH the candidate genes identified by differential expression analysis. Given the representation of olfactory bulb tissue in the “inner ONL” transcriptome, we used “outer ONL” for transcriptomic comparisons with peripheral olfactory nerve and trigeminal nerve at E16.5, to identify genes differentially expressed by OEC subpopulations and Schwann cells, which could be important for controlling their diversification.

### 3.3 OECs at E16.5 express multiple genes involved in oligodendrocyte development

**Figure 2:**
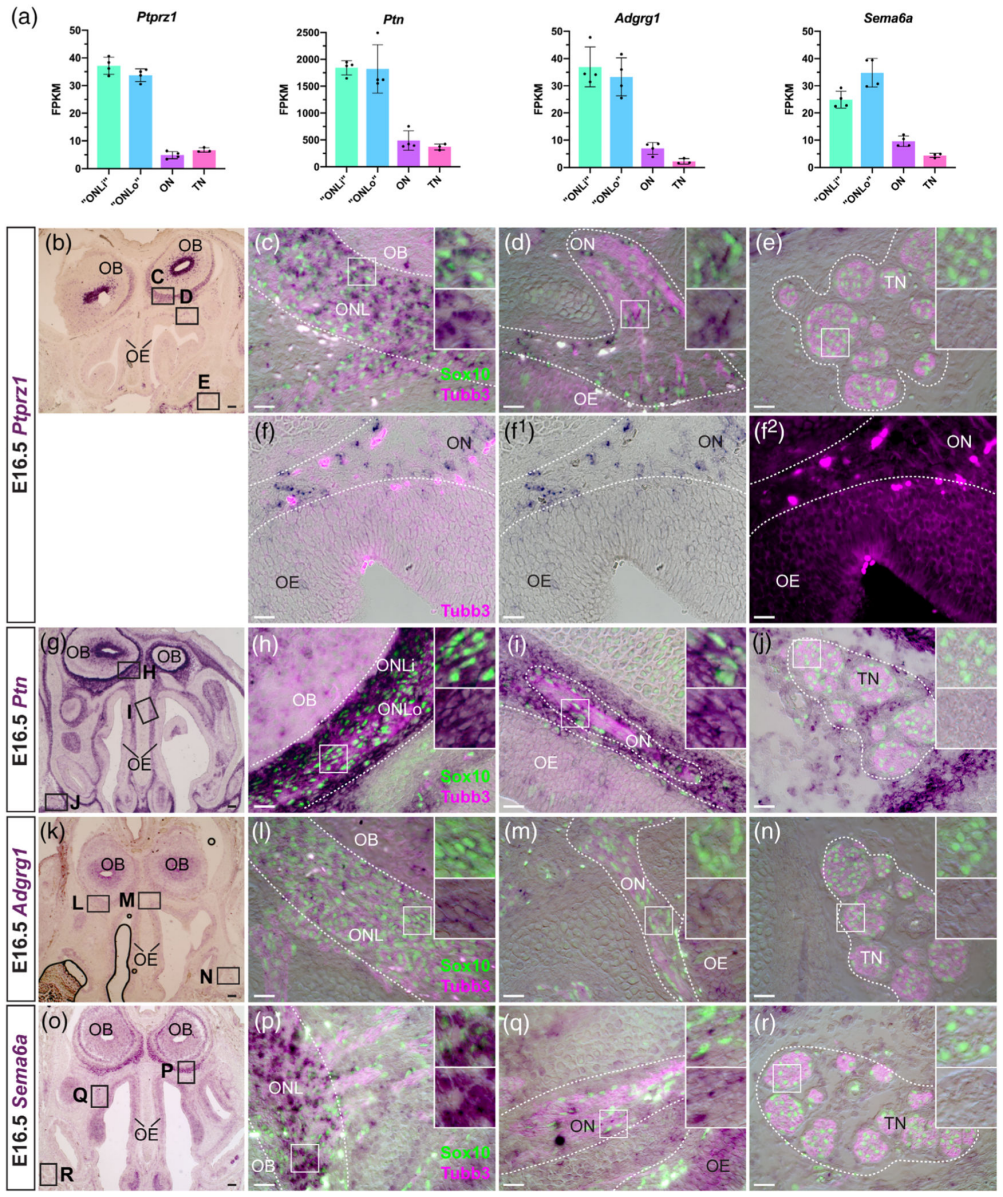
Four genes involved in oligodendrocyte development are OEC-specific at E16.5. (a) Bar charts showing mean expression values across all transcriptomes at E16.5 for *Ptprz1, Ptn, Adgrg1,* and *Sema6a.* Error bars indicate SD. (b-r) Coronal sections through the mouse olfactory system at E16.5, immunostained for Sox10 (green nuclei) to identify OECs and Schwann cells and for Tubb3 (magenta) to identify axons, following in situ hybridization for: (b-f^2^) *Ptprz1,* which is expressed by ONL-OECs and mucosal OECs, but not trigeminal Schwann cells on the trigeminal nerve (or olfactory neurons in the olfactory epithelium: panels [f-f^2^] show a slightly more ventral region than in [d], including more of the olfactory epithelium) (*n* = 10); (g-j) Ptn, which is expressed by ONL-OECs, most strongly by inner ONL-OECs (and mesenchyme around the ONL), mucosal OECs (and mesenchyme around olfactory nerve fascicles), but not trigeminal Schwann cells, though there is some expression in adjacent mesenchyme (*n* = 10); (k-n) *Adgrg1 (Gpr56),* which is weakly expressed by ONL-OECs and by at least some mucosal OECs, but not trigeminal Schwann cells (*n* = 4); (o-r) *Sema6a,* which is expressed by ONL-OECs and mucosal OECs, but not trigeminal Schwann cells (*n* = 4). Scale bars: (b,g,k,o) 100 μm; (c-f^2^,h-j,l-n,p-r) 25 μm. FPKM, fragments per kilobase of transcript per million mapped reads; OB, olfactory bulb; OE, olfactory epithelium; OEC, olfactory ensheathing cell; ON, olfactory nerve; ONL, olfactory nerve layer; ONLi, inner olfactory nerve layer; ONLo, outer olfactory nerve layer; TN, trigeminal nerve [Color figure can be viewed at wileyonlinelibrary.com]

Our differential expression analysis at E16.5 (Figure 2a) and ISH validation at the same stage (Figure 2b-r) identified four pan-OEC-specific genes (i.e., not expressed by trigeminal Schwann cells) that have been implicated in controlling the development of oligodendrocytes (the myelinating glia of the CNS). All four genes—*Ptprz1, Ptn, Adgrg1 (Gpr56),* and *Sema6a—* were enriched in ONL transcriptomes over both olfactory nerve and trigeminal nerve transcriptomes at E16.5 (Figure 2a).

*Ptprz1* encodes receptor-type tyrosine protein phosphatase zeta (Ptprz), whose activity maintains oligodendrocyte precursor cells in an undifferentiated state (Kuboyama et al., 2012). Binding of the secreted heparin-binding growth factor pleiotrophin (encoded by *Ptn*) represses Ptprz activity and promotes oligodendrocyte differentiation, myelination, and remyelination (Kuboyama et al., 2012; Kuboyama et al., 2015; Kuboyama et al., 2016). At E16.5, *Ptrpz1* was expressed by both ONL-OECs and mucosal OECs (and also by scattered cells deep inside the olfactory bulb, presumably oligodendrocyte precursor cells), but not by trigeminal Schwann cells (Figure 2b-f^2^). Although the differential expression analysis showed apparently negligible levels of *Ptprz1* expression in the olfactory nerve (Figure 2a), ISH revealed clear expression in mucosal OECs (Figure 2d,f,f^1^). This is likely due to the much lower density of mucosal OECs versus ONL-OECs (compare Figures 2c,d).

*Ptn* similarly proved to be expressed by all OECs, with particularly strong expression in the inner ONL, but not by trigeminal Schwann cells (Figure 2g-j). Scattered cells in the olfactory epithelium seemed to express *Ptn*, but only at low levels (Figure 2i). Strong *Ptn* expression was seen in a thin layer of mesenchyme immediately surrounding the ONL and olfactory nerve fascicles (Figure 2h,i), and in mesenchyme surrounding trigeminal nerve fascicles (Figure 2j). The latter likely explains the relatively high *Ptn* expression levels in the trigeminal nerve transcriptome despite the lack of expression in Schwann cells (Figure 2a).

*Adgrg1* (*Gpr56*) encodes an adhesion-class G protein-coupled receptor that promotes oligodendrocyte precursor cell proliferation and inhibits oligodendrocyte differentiation (Ackerman, Garcia, Piao, Gutmann, & Monk, 2015; Giera et al., 2015; Giera et al., 2018). Like *Ptprz1* and *Ptn*, *Adgrg1* was pan-OEC-specific at E16.5, albeit with only weak expression (Figure 2k-n), as was *Sema6a* (Figure 2o-r), which encodes a transmembrane semaphorin that can also signal cell-autonomously (Perez-Branguli et al., 2016). *Sema6a* is expressed by oligodendrocyte precursor cells and may be involved in their migration (Okada & Tomooka, 2012; Okada & Tomooka, 2013). It is also highly expressed by postnatal oligodendrocytes and its deletion delays their differentiation (Bernard et al., 2012).

Furthermore, although not specific to OECs, ISH confirmed stronger expression of the SoxD-class transcription factor gene *Sox5* in ONL-OECs than in mucosal OECs or trigeminal Schwann cells (Figure S4a-e). In oligodendrocyte development, Sox5 and the closely related transcription factor Sox6 act together to repress oligodendrocyte specification and terminal differentiation (Baroti et al., 2016; Stolt et al., 2006). Finally, we identified expression in both OECs and Schwann cells of *Cntn1* (Figure S4a,f-i), which encodes a membrane-tethered cell-adhesion molecule that binds Ptprz, promoting oligodendrocyte differentiation (Lamprianou, Chatzopoulou, Thomas, Bouyain, & Harroch, 2011). *Cntn1* may also be expressed by olfactory axons, given its high expression level in the olfactory epithelium (Figure S4f,h).

Overall, these expression data suggest potential parallels between the development of OECs (perhaps especially ONL-OECs) and oligo-dendrocytes that distinguish them from trigeminal Schwann cells.

### 3.4 The expression of different Wnt pathway-modulating genes distinguishes ONL-OECs from mucosal OECs and Schwann cells at E16.5

**Figure 3:**
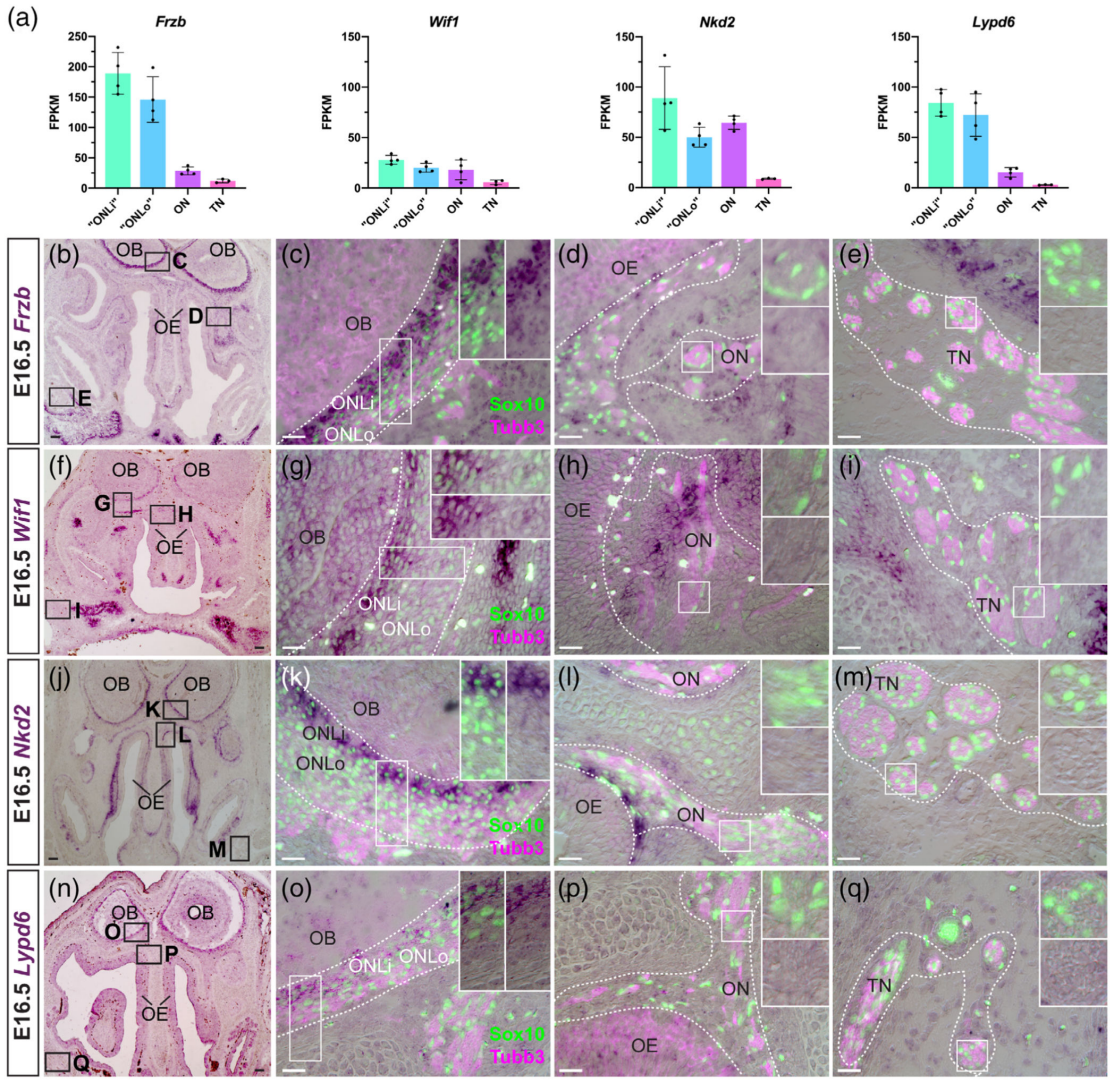
ONL-OECs express multiple Wnt pathway-modulating genes at E16.5 that distinguish them from mucosal OECs and trigeminal Schwann cells. (a) Barcharts showing mean expression values across all transcriptomes at E16.5 for *Frzb, Wif1, Nkd,* and *Lypd6.* Error bars indicate SD. (b-q) Coronal sections through the mouse olfactory system at E16.5, immunostained for Sox10 (green nuclei) to identify OECs and Schwann cells and for Tubb3 (magenta) to identify axons, following in situ hybridization for: (b-e) *Frzb,* which is expressed by ONL-OECs (more strongly by inner ONL-OECs) and by a few mucosal OECs, but not by trigeminal Schwann cells (*n* = 6 at E15.6-16.5); (f-i) *Wif1,* which is expressed strongly by inner ONL-OECs and more weakly by outer ONL-OECs, but not by mucosal OECs (though there is expression in patches of mesenchyme near the olfactory epithelium) or trigeminal Schwann cells (*n* = 4 at E15.6-16.5); (j-m) *Nkd2,* which is expressed strongly by inner ONL-OECs and more weakly by outer ONL-OECs, but not by mucosal OECs (though there is strong expression in mesenchyme near the olfactory epithelium) or trigeminal Schwann cells (*n* = 6 at E15.6-16.5); (n-q) *Lypd6,* which is expressed strongly by inner ONL-OECs and more weakly by outer ONL-OECs, but not by mucosal OECs or trigeminal Schwann cells (*n* = 4 at E15.6-16.5). Scale bars: (b,f,j,n) 100 μm; (c-e,g-i,k-m,o-q) 25 μm. FPKM, fragments per kilobase of transcript per million mapped reads; OB, olfactory bulb; OE, olfactory epithelium; OEC, olfactory ensheathing cell; ON, olfactory nerve; ONL, olfactory nerve layer; ONLi, inner olfactory nerve layer; ONLo, outer olfactory nerve layer; TN, trigeminal nerve [Color figure can be viewed at wileyonlinelibrary.com]

We recently reported that *Frzb,* encoding a secreted Frizzled-related protein that inhibits Wnt signalling, is expressed by ONL-OECs at E16.5 (most strongly in the inner ONL), but only by a few mucosal OECs, and that deletion of *Frzb* disrupts olfactory axon targeting (Rich et al., 2018). Our RNA-seq data at E16.5 were consistent with this and further suggested that *Frzb* is not expressed by trigeminal Schwann cells (Figure 3a), which we validated by ISH at E15.5-16.5 (Figure 3b-e).

We also identified three Wnt pathway-modulating genes with strong expression in inner ONL-OECs and weaker expression in outer ONL-OECs, but no expression in mucosal OECs or trigeminal Schwann cells at E15.5-16.5. Two of these genes act to inhibit Wnt signalling. *Wif1* (Figure 3a,f-i), like *Frzb*, encodes a secreted Wnt inhibitor (Hsieh et al., 1999), whereas *Nkd2* (Figure 3a,j-m) encodes a myristoylated protein that cell-autonomously inhibits Wnt/beta-catenin signalling by binding to Dishevelled1 at the plasma membrane (Hu et al., 2010). In contrast, *Lypd6* (Figure 3a,n-q) encodes a membrane-associated Wnt/beta-catenin signalling feedback enhancer (Özhan et al., 2013). Expression of *Wif1* and *Nkd2* in mesenchyme near the olfactory epithelium likely explains the relatively high expression levels of these genes in the olfactory nerve transcriptome (Figure 3a).

**Figure 4:**
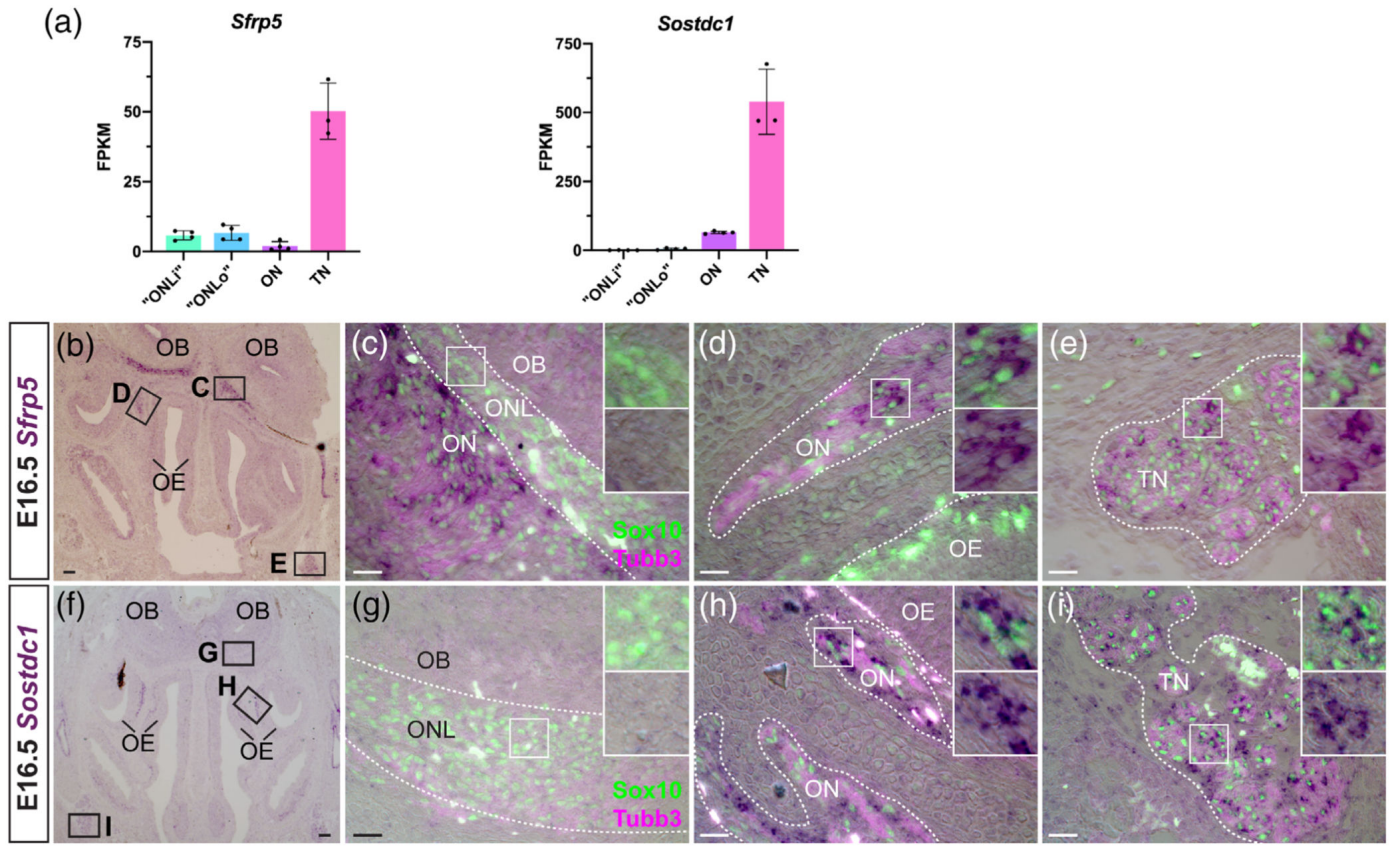
Mucosal OECs and trigeminal Schwann cells express secreted Wnt-inhibitor genes at E16.5 that distinguish them from ONL-OECs. (a) Bar charts showing mean expression values across all transcriptomes at E16.5 for *Sfrp5* and *Sostdc1 (Wise).* Error bars indicate SD. (b-i) Coronal sections through the mouse olfactory system at E16.5, immunostained for Sox10 (green nuclei) to identify OECs and Schwann cells and for Tubb3 (magenta) to identify axons, following in situ hybridization for: (b-e) *Sfrp5,* which is not expressed by ONL-OECs but shows strong expression in mucosal OECs and trigeminal Schwann cells (*n* = 5); (f-i) *Sostdc1 (Wise),* which is not expressed by ONL-OECs but shows weak expression in mucosal OECs (and strong expression in Sox10-negative, Tubb3-positive cells on the olfactory nerve, presumably migratory neurons), and strong expression in trigeminal Schwann cells (*n* = 4). Scale bars: (b,f) 100 μm; (c-e,g-i) 25 μm. FPKM, fragments per kilobase of transcript per million mapped reads; OB, olfactory bulb; OE, olfactory epithelium; OEC, olfactory ensheathing cell; ON, olfactory nerve; ONL, olfactory nerve layer; ONLi, inner olfactory nerve layer; ONLo, outer olfactory nerve layer; TN, trigeminal nerve [Color figure can be viewed at wileyonlinelibrary.com]

Conversely, we found expression of two different secreted Wnt inhibitor genes in mucosal OECs and Schwann cells, but not ONL-OECs: *Sfrp5* (Figure 4a-e) and the secreted dual Wnt/Bmp inhibitor gene *Sostdc1 (Wise;* Figure 4a,f-i; also expressed strongly by Sox10-negative, Tubb3-positive cells on the olfactory nerve, presumably migrating neurons).

Overall, these expression data at E16.5 suggest that both noncell-autonomous and cell-autonomous modulation (primarily inhibition) of Wnt signalling by ONL-OECs is important for OEC development and/or function, particularly in the inner ONL. This is consistent with our previous demonstration that *Frzb* is required for olfactory axon targeting (Rich et al., 2018). However, the expression of different Wnt inhibitor genes in mucosal OECs and Schwann cells suggests that Wnt inhibition may also be important for shared aspects of the development and/or function of these peripheral nerve glial cells, potentially different from ONL-OECs.

### 3.5 OEC subpopulations and Schwann cells at E16.5 differentially express genes encoding proteins involved in axon guidance, neurite outgrowth, and/or cell migration

**Figure 5:**
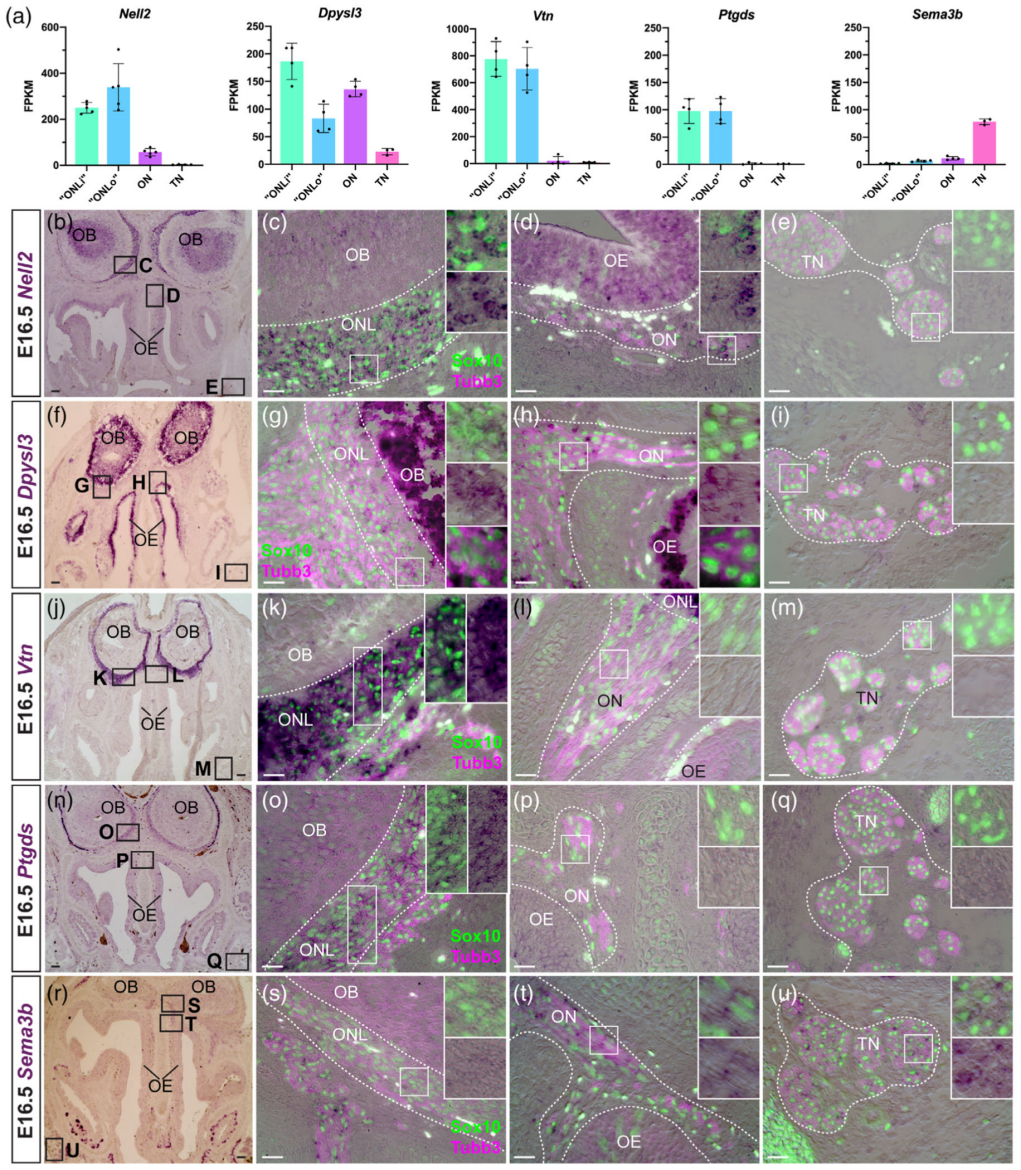
Genes involved in axon guidance, neurite outgrowth and/or cell migration are differentially expressed between OEC subpopulations and trigeminal Schwann cells at E16.5. (a) Bar charts showing mean expression values across all transcriptomes at E16.5 for *Nell2*, *Dpysl3 (Crmp4), Vtn, Ptgds,* and *Sema3b.* Error bars indicate SD. (b-u) Coronal sections through the mouse olfactory system at E16.5, immunostained for Sox10 (green nuclei) to identify OECs and Schwann cells and for Tubb3 (magenta) to identify axons, following in situ hybridization for: (b-e) *Nell2,* which is expressed by ONL-OECs and mucosal OECs, but not trigeminal Schwann cells (*n* = 5); (f-i) *Dpysl3 (Crmp4),* which is expressed by ONL-OECs (more strongly by inner ONL-OECs; also very strongly by the olfactory bulb) and mucosal OECs (also very strongly by the olfactory epithelium and scattered Sox10-negative, Tubb3-positive cells on the olfactory nerve, presumably migrating neurons), but not by trigeminal Schwann cells (*n* = 5); (j-m) *Vtn*, which is expressed by ONL-OECs, but not by mucosal OECs or trigeminal Schwann cells (*n* = 5); (n-q) *Ptgds,* which is expressed very strongly in the leptomeninges (visible at low-power in panel [n] as the thin, dark-blue stripe lateral to the olfactory bulbs), as expected (Urade & Hayaishi, 2000), and by ONL-OECs, but not by mucosal OECs or trigeminal Schwann cells (*n* = 5); (r-u) *Sema3b,* which is not expressed by ONL-OECs, but is expressed by mucosal OECs and by trigeminal Schwann cells (*n* = 5). Scale bars: (b,f,j,n,r) 100 μm; (c-e,g-i,k-m,o-q,s-u) 25 μm. FPKM, fragments per kilobase of transcript per million mapped reads; OB, olfactory bulb; OE, olfactory epithelium; OEC, olfactory ensheathing cell; ON, olfactory nerve; ONL, olfactory nerve layer; ONLi, inner olfactory nerve layer; ONLo, outer olfactory nerve layer; TN, trigeminal nerve [Color figure can be viewed at wileyonlinelibrary.com]

The failure of normal OEC differentiation in Sox10-null mice results in defects in both olfactory axon targeting and GnRH neuron migration to the olfactory bulb (Barraud et al., 2013; Pingault et al., 2013). We described above our identification of *Sema6a* as a novel pan-OEC-specific marker at E16.5. This transmembrane semaphorin has roles in axon guidance (Jongbloets & Pasterkamp, 2014) as well as in oligodendrocyte differentiation (Bernard et al., 2012; Okada & Tomooka, 2012; Okada & Tomooka, 2013). Our differential expression analysis at E16.5 (Figure 5a) and ISH validation at the same stage (Figure 5b-u) identified five other genes encoding proteins involved in axon guidance, neurite outgrowth, and/or cell migration that were differentially expressed in OECsubpopulations and/or trigeminal Schwann cells.

*Nell2* (Figure 5a-e), encoding a secreted axon repellant (Jaworski et al., 2015), and *Dpysl3* (also known as collapsin response-mediator protein 4, *Crmp4;* Figure 5a,f-i), encoding an F-actin-bundling, microtubule-stabilising protein that inhibits process outgrowth and cell migration (Nagai, Baba, & Ohshima, 2017), were both pan-OEC-specific at E16.5, that is, expressed by ONL-OECs and mucosal OECs but not trigeminal Schwann cells, like *Sema6a* (Figure 2a,o-r). (*Dpysl3* was also expressed by scattered Sox10-negative cells on the olfactory nerve, likely migrating neurons.)

ONL-OEC-specific genes (i.e., not expressed by mucosal OECs or trigeminal Schwann cells) included *Vtn* (Figure 5a,j-m), encoding the secreted extracellular matrix-associated glycoprotein vitronectin, which has multiple roles including in neurite outgrowth and neuronal migration (Katic et al., 2014; Plantman, 2013); and *Ptgds* (Figure 5a,n-q), encoding lipocalin-type prostaglandin-D synthase (a multifunctional protein that synthesizes prostaglandin D2 and acts as an extracellular transporter of small hydrophobic molecules, including retinoic acid; Urade & Hayaishi, 2000), which promotes astrocyte migration (Lee et al., 2012) and is also expressed by adult oligodendrocytes and oligodendrocyte precursor cells (Sakry, Yigit, Dimou, & Trotter, 2015; Urade & Hayaishi, 2000).

Finally, mucosal OECs and trigeminal Schwann cells, but not ONL-OECs, expressed *Sema3b* (Figure 5a,r-u), encoding a secreted axon guidance molecule (Jongbloets & Pasterkamp, 2014).

Taken together, these data show that different OEC subpopulations express different axon guidance cues (mucosal but not ONL-OECs: Sema3b) and other secreted proteins involved in neurite outgrowth and/or cell migration (ONL-OECs but not mucosal OECs: vitronectin, lipocalin-type Ptgds). This is consistent with different OEC subpopulations interacting differently with axons (Ekberg et al., 2012) and potentially also migrating neurons.

### 3.6 OEC subpopulations and trigeminal Schwann cells at E16.5 differentially express genes encoding myelin-associated proteins

**Figure 6:**
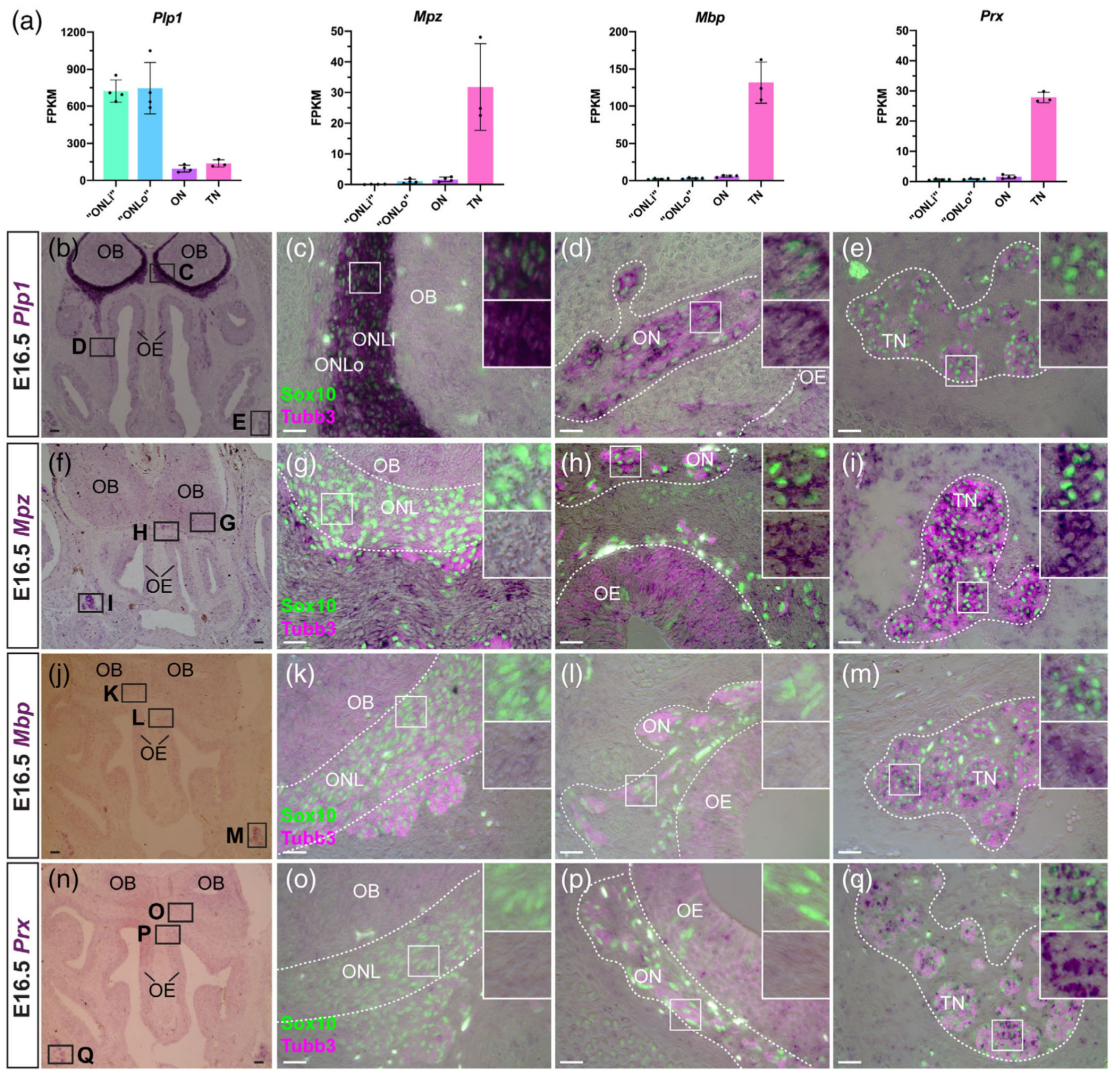
OEC subpopulations and trigeminal Schwann cells differentially express genes encoding myelin-associated proteins at E16.5. (a) Bar charts showing mean expression values across all transcriptomes at E16.5 for *Plp1, Mpz, Mbp,* and Prx. Error bars indicate SD. (b-q) Coronal sections through the mouse olfactory system at E16.5, immunostained for Sox10 (green nuclei) to identify OECs and Schwann cells and for Tubb3 (magenta) to identify axons, following in situ hybridization for: (b-e) *Plp1,* which is very strongly expressed by ONL-OECs (most strongly by inner ONL-OECs), and less strongly by mucosal OECs and trigeminal Schwann cells (*n* = 10); (f-i) Mpz, which is not expressed by ONL-OECs but by mucosal OECs and trigeminal Schwann cells (*n* = 4); (j-m) Mbp, which is not expressed by ONL-OECs or mucosal OECs, but is expressed by trigeminal Schwann cells (*n* = 4); (n-q) Prx, which is not expressed by ONL-OECs or mucosal OECs, but is expressed by trigeminal Schwann cells (*n* = 4). Scale bars: (b,f,j,n) 100 μm; (c-e,g-i,k-m,o-q) 25 μm. FPKM, fragments per kilobase of transcript per million mapped reads; OB, olfactory bulb; OE, olfactory epithelium; OEC, olfactory ensheathing cell; ON, olfactory nerve; ONL, olfactory nerve layer; ONLi, inner olfactory nerve layer; ONLo, outer olfactory nerve layer; TN, trigeminal nerve [Color figure can be viewed at wileyonlinelibrary.com]

OECs are not myelinating in situ but will myelinate larger-diameter axons in vitro and in vivo (see, e.g., Devon & Doucette, 1992; Franklin, 2003; Babiarz et al., 2011; Guérout et al., 2011). Our differential expression analysis (Figure 6a) and ISH validation (Figure 6b-q) identified several genes encoding myelin-associated proteins that were differentially expressed by different OEC subpopulations and/or trigeminal Schwann cells at E16.5.

Expression of the shorter *Dm20* splice isoform of *Plpi*, encoding proteolipid protein 1 (the major protein constituent of compact CNS myelin; Han, Myllykoski, Ruskamo, Wang, & Kursula, 2013), was previously reported in ONL-OECs from E14 (Griffiths, Dickinson, & Montague, 1995; Dickinson et al., 1997; also see Piantanida et al., 2019). Our E16.5 transcriptome data suggested quantitatively different levels of expression of *Plpi* (our riboprobe does not distinguish the splice isoforms) in the different glial subpopulations, with very high levels in the ONL transcriptomes, and much lower (albeit still significant) levels in the olfactory nerve and trigeminal nerve transcriptomes (Figure 6a). This was confirmed by ISH, which revealed significantly stronger *Plp1* expression in ONL-OECs (most strongly in inner ONL-OECs) than in either mucosal OECs or trigeminal Schwann cells on the same sections (Figure 6b-e).

We also examined the expression of Mpz, encoding myelin protein zero (Mpz, P0: a major component of peripheral myelin; Han et al., 2013), which is a direct Sox10 target gene (Jacob, 2015) expressed in developing rat and chicken OECs (Drapkin & Silverman, 1999; Norgren, Ratner, & Brackenbury, 1992; ), as well as Schwann cell precursors (Jessen & Mirsky, 2019). The E16.5 trans-criptome data showed minimal levels of *Mpz* in the olfactory nerve transcriptome, versus robust expression in the trigeminal nerve trans-criptome (Figure 6a). Nevertheless, ISH revealed expression in mucosal OECs (though not ONL-OECs), as well as confirming expression in trigeminal Schwann cells (Figure 6f-i).

Although we focused in this study on genes likely to be expressed by some or all OEC subpopulations, we found that trigeminal Schwann cells, but not OECs, express *Mbp* (Figure 6a,j-m). This direct Sox10 target gene (Stolt et al., 2002) encodes myelin basic protein, a major constituent of both peripheral and CNS myelin (Han et al., 2013). Likewise, trigeminal Schwann cells but not OECs express *Prx* (Figure 6a,n-q), encoding periaxin, which maintains peripheral myelin (Han et al., 2013).

Taken together, these data show that the expression of different myelin-associated proteins distinguishes OEC subpopulations (all of which are nonmyelinating in situ) and trigeminal Schwann cells at E16.5: *Plp1* is significantly enriched in ONL-OECs (particularly inner ONL-OECs) over mucosal OECs and trigeminal Schwann cells (our riboprobe recognizes both *Plpi* and the shorter *Dm20* splice variant, whose expression was previously reported in ONL-OECs; Griffiths et al., 1995; Dickinson et al., 1997). *Mpz* is specific to mucosal OECs and trigeminal Schwann cells, while *Mbp* and *Prx* are restricted to trigeminal Schwann cells. *Plpi* and *Mpz* (and *Mbp*) are also expressed by mature enteric glia, which are also nonmyelinating, suggesting that these proteins (for Plp1, most likely the Dm20 isoform; Griffiths et al., 1995; Dickinson et al., 1997) have additional, nonstructural roles (Rao et al., 2015).

### 3.7 OEC subpopulations and trigeminal Schwann cells express different Notch transcriptional effectors at E16.5

**Figure 7:**
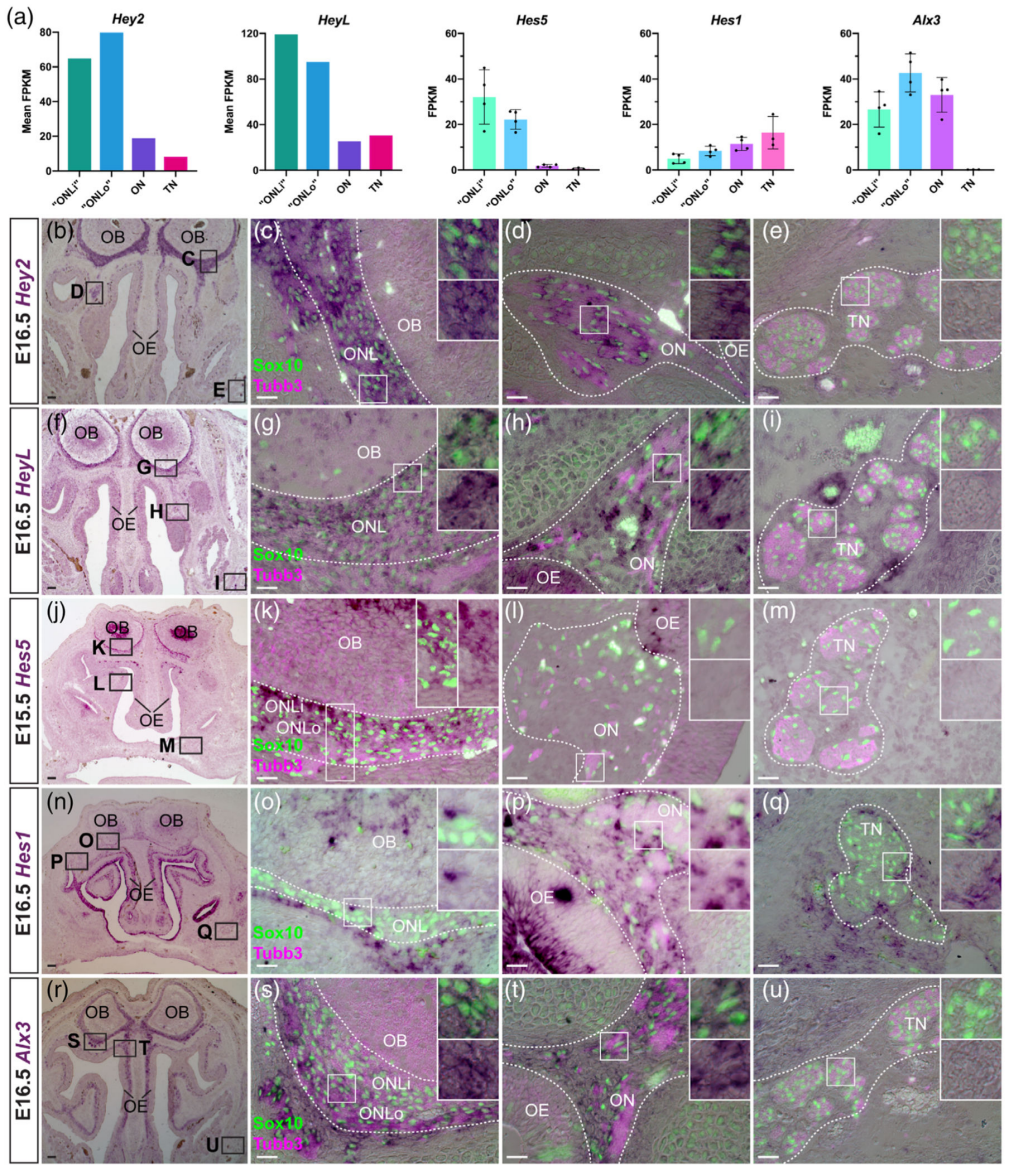
OEC-specific genes at E16.5 include three Notch transcriptional effector genes and *Alx3.* (a) Bar charts showing mean expression values across all transcriptomes at E16.5 for the Notch/Rbpj pathway transcriptional effector genes *Hey2, HeyL, Hes5,* and *Hes1,* plus the homeobox transcription factor gene *Alx3.* Error bars indicate *SD*. (b–u) Coronal sections through the mouse olfactory system at E15.5-16.5, immunostained for Sox10 (green nuclei) to identify OECs and Schwann cells and for Tubb3 (magenta) to identify axons, following in situ hybridization for: (b-e) *Hey2,* which is expressed by ONL-OECs and mucosal OECs, but not trigeminal Schwann cells (*n* = 6); (f–i) *HeyL,* which is expressed by ONL-OECs and at least some mucosal OECs, but not trigeminal Schwann cells (as well as by vascular cells surrounding autofluorescent blood cells) (*n* = 6); (j–m) *Hes5,* which is expressed by ONL-OECs (more strongly by inner ONL-OECs), but not by mucosal OECs or trigeminal Schwann cells (*n* = 6); (nj–q) *Hes1,* which is not expressed by ONL-OECs (although there is expression in mesenchyme surrounding the ONL), but which seems to be expressed by at least some mucosal OECs and a few trigeminal Schwann cells (as well as by mesenchymal cells near both olfactory and trigeminal nerve fascicles) (*n* = 6); (rj–u) *Alx3,* which is expressed by ONL-OECs (more strongly by outer ONL-OECs) and by mucosal OECs, but not by trigeminal Schwann cells (*n* = 6). Scale bars: (b,f,j,n,r) 100 μm; (cj–e,gj–i,kj–m,o-q,sj–u) 25 μm. FPKM, fragments per kilobase of transcript per million mapped reads; OB, olfactory bulb; OE, olfactory epithelium; OEC, olfactory ensheathing cell; ON, olfactory nerve; ONL, olfactory nerve layer; ONLi, inner olfactory nerve layer; ONLo, outer olfactory nerve layer; TN, trigeminal nerve [Color figure can be viewed at wileyonlinelibrary.com]

The transition from Schwann cell precursor to immature Schwann cell is promoted by canonical Notch signalling transduced by the Rbpj transcription factor (Woodhoo et al., 2009). Our differential expression analysis at E16.5 (Figure 7a) and ISH validation at E15.5-16.5 (Figure 7b-q) revealed differential expression by OEC subpopulations and trigeminal Schwann cells, of four *hairy/enhancer-of-split-related (Hes, Hey)* basic helix-loop-helix (bHLH) transcriptional repressor genes, all direct targets and effectors of Notch/Rbpj signalling (Dennis, Han, & Schuurmans, 2019; Weber, Wiese, & Gessler, 2014).

*Hey2* (Figure 7a-e) was pan-OEC-specific, that is, expressed by ONL-OECs and mucosal OECs, but not by trigeminal Schwann cells. *HeyL* (Figure 7a,f-i) was expressed by ONL-OECs (expression was previously reported surrounding the olfactory bulb; Leimeister, Schumacher, Steidl, & Gessler, 2000) and at least some mucosal OECs, but not trigeminal Schwann cells. (Strong *HeyL* expression was also seen in vascular cells, presumably smooth muscle as previously reported; Leimeister et al., 2000.) *Hes5* (Figure 7a,j-m) was expressed by ONL-OECs (more strongly by inner ONL-OECs), but not by either mucosal OECs or trigeminal Schwann cells. Conversely, *Hes1* (Figure 7a,n-q) was not expressed by ONL-OECs, but seemed to be expressed by at least some mucosal OECs and a few trigeminal Schwann cells. *Hes1* expression in mesenchyme adjacent to the ONL and to olfactory and trigeminal nerve fascicles likely explains why this gene was detected in the nerve transcriptomes. Oscillatory expression of *Hes1* (Imayoshi & Kageyama, 2014; Kageyama, Shimojo, & Imayoshi, 2015) could explain its expression in only a subset of mucosal OECs and trigeminal Schwann cells.

We also identified one additional pan-OEC-specific transcription factor: the homeobox gene *Alx3* (Figure 7a,r–u). In contrast to *Hey2* and *HeyL*, *Alx3* was expressed more strongly by outer ONL-OECs and mucosal OECs than by inner ONL-OECs (Figure 7r-u).

Taken together, the differing expression patterns of these Notch transcriptional effector genes at E16.5 (*Hey2* and *HeyL* in ONL-OECs and mucosal OECs but not trigeminal Schwann cells; *Hes5* restricted to ONL-OECs; *Hesi* in at least some mucosal OECs and trigeminal Schwann cells, but not in ONL-OECs) suggest roles for Notch/Rbpj signalling via these different *Hey/Hes* genes in the development of OECs versus Schwann cells, with Notch signalling via *Hes5* potentially being important specifically for ONL-OECs.

### 3.8 Identification of novel markers for OECs that are also expressed by Schwann cells

**Figure 8:**
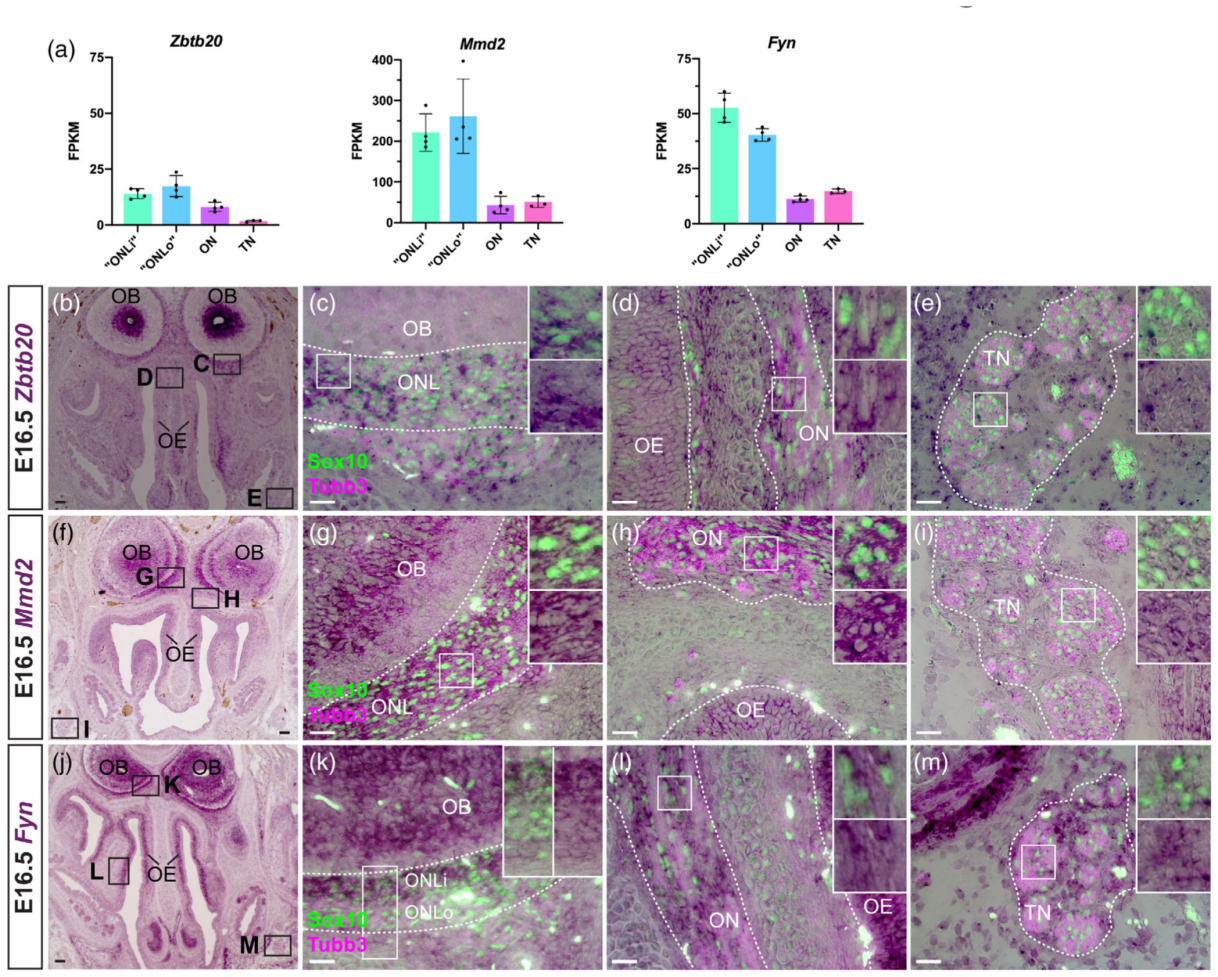
Novel markers for OECs expressed at higher levels in some or all OECs than in trigeminal Schwann cells at E16.5. (a) Bar charts showing mean expression values across all transcriptomes at E16.5 for *Zbtb20, Mmd2,* and Fyn. Error bars indicate *SD*. (b–q) Coronal sections through the mouse olfactory system at E16.5, immunostained for Sox10 (green nuclei) to identify OECs and Schwann cells and for Tubb3 (magenta) to identify axons, following in situ hybridization for: (b–e) *Zbtb20,* which is expressed more strongly by ONL-OECs than by mucosal OECs or trigeminal Schwann cells (*n* = 4); (f–i) *Mmd2,* which is expressed more strongly by ONL-OECs and mucosal OECs than by trigeminal Schwann cells (*n* = 3); (j–m) Fyn, which is expressed more strongly by inner ONL-OECs than by outer ONL-OECs, mucosal OECs, or trigeminal Schwann cells (*n* = 3). Scale bars: (b,f,j) 100 μm; (c–e,g–i,k–m) 25 μm. FPKM, fragments per kilobase of transcript per million mapped reads; OB, olfactory bulb; OE, olfactory epithelium; OEC, olfactory ensheathing cell; ON, olfactory nerve; ONL, olfactory nerve layer; ONLi, inner olfactory nerve layer; ONLo, outer olfactory nerve layer; TN, trigeminal nerve [Color figure can be viewed at wileyonlinelibrary.com]

We identified several other novel markers for OECs at E16.5 that, like *Sox5* (Figure S4a-e) and *Plp1* (Figure 6a,r-u), were expressed at significantly higher levels in the ONL transcriptomes versus the olfactory and trigeminal nerve transcriptomes, and for which validation by ISH confirmed expression at some level in all OECs and trigeminal Schwann cells. These were the transcription factor gene *Zbtb20* (Figure 8a,b-e), the integral membrane (mitochondrial or Golgi) protein gene *Mmd2* (Figure 8a,f-i), and the Src family tyrosine kinase gene *Fyn* (Figure 8a,j-m). Although these genes were expressed by all OECs and trigeminal Schwann cells, *Mmd2* showed detectably higher expression in OECs versus trigeminal Schwann cells on the same sections (Figure 8g-i), while *Fyn* was enriched in inner ONL-OECs over outer ONL-OECs (Figure 8k-m).

### 3.9 Expression summary for 25 novel OEC markers identified at E16.5

File S5 and Figure S5 summarize the transcriptome expression levels and ISH expression patterns at E16.5 for the 25 novel genes validated as being expressed by some or all OEC subpopulations at E16.5, together with four previously identified markers for some or all embryonic mouse OECs that we also validated in the current study: the inner ONL-OEC-specific gene *Prss56* (Jourdon et al., 2016); the ONL-OEC-specific gene *Npy,* which is enriched in inner ONL-OECs (Au et al., 2002; Barraud et al., 2013); the OEC-specific gene *Frzb* (enriched in inner ONL-OECs but also expressed by some mucosal OECs; Rich et al., 2018); and the pan-OEC and Schwann cell marker *Sox10* (Barraud et al., 2010; Barraud et al., 2013; Forni et al., 2011; Jessen et al., 2015).

The 25 novel markers identified for mouse OECs at E16.5 comprise four ONL-OEC-specific genes (not expressed by mucosal OECs or trigeminal Schwann cells) that are enriched in inner ONL-OECs *(Hes5, Lypd6, Nkd2, Wif1);* two additional ONL-OEC-specific genes (*Ptgds*, *Vtn*); nine pan-OEC-specific genes (expressed by ONL-OECs and mucosal OECs but not by trigeminal Schwann cells: *Adgrg1* [*Gpr56*], *Alx3*, *Dpysl3* [*Crmp4*], *Hey2*, *HeyL*, *Nell2*, *Ptn*, *Ptprzl*, *Sema6a*); five genes expressed by mucosal OECs and trigeminal Schwann cells but not by ONL-OECs (*Hes1* [some cells within each population], *Mpz*, *Sema3b*, *Sfrp5*, *Sostdc1* [*Wise*]), and five genes expressed at some level by ONL-OECs, mucosal OECs, and trigeminal Schwann cells: *Sox5* was enriched in ONL-OECs over mucosal OECs and trigeminal Schwann cells; *Mmd2* was enriched in OECs over trigeminal Schwann cells; *Fyn* was enriched in inner ONL-OECs over outer ONL-OECs; *Cntn1* and *Zbtb20* showed no obvious difference across the various glial populations.

### 3.10 Pan-OEC markers at E16.5 are already expressed by mucosal OECs at E11.5

To gain insight into the onset and pattern of OEC differentiation, we assessed the expression of the E16.5 OEC marker genes at earlier stages. We checked their transcriptome expression levels at E11.5, when only mucosal OECs are present, and at E13.5, when the ONL is starting to thicken (File S6), and validated their expression by ISH.

**Figure 9:**
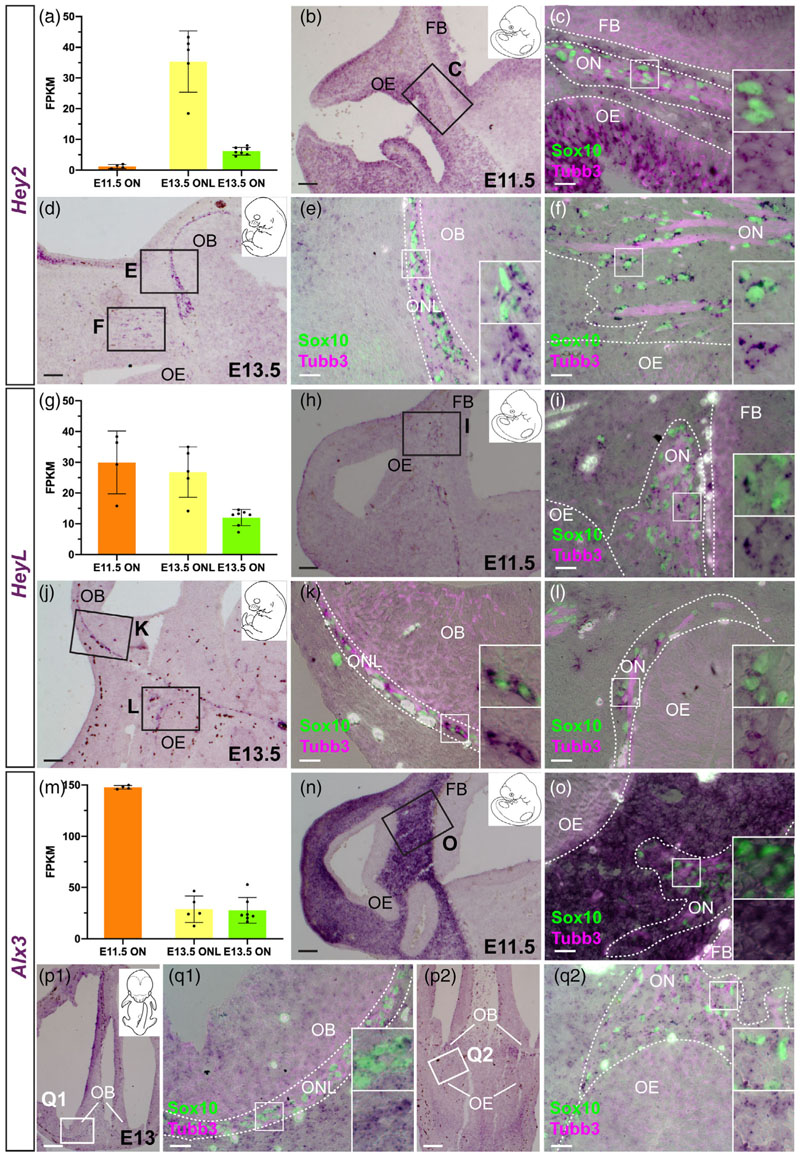
Mucosal OECs at E11.5 already express transcription factor genes that are pan-OEC-specific at E16.5. Bar charts showing mean expression values across all transcriptomes at E11.5 and E13.5 (error bars indicate *SD*), and parasagittal or coronal sections through the mouse olfactory system at E11.5 or E13.0–13.5 (orientation identified by redrawn Theiler stage schematics from the EMAP eMouse Atlas Project, http://www.emouseatlas.org; Richardson et al., 2014), immunostained for Sox10 (green nuclei) to identify OECs and Schwann cells and for Tubb3 (magenta) to identify axons, following in situ hybridization for: (a-f) *Hey2 (n* = 2 at E11.5; *n* = 3 at E13.0–13.5); (g-l) *HeyL* (*n* = 2 at E11.5; *n* = 3 at E13.0–13.5); (m-q2) *Alx3* (*n* = 2 at E11.5; *n* = 3 at E13.0–13.5; panels [p1-q2] show sections from the same embryo). All three genes are expressed by mucosal OECs at E11.5 and by both ONL-OECs and mucosal OECs at E13.0–13.5. Scale bars: (b,d,h,j, n,p1,p2) 100 μm; (c,e,f,i,k,l,o, q1,q2) 25 μm. FB, forebrain; FPKM, fragments per kilobase of transcript per million mapped reads; OB, olfactory bulb; OE, olfactory epithelium; OEC, olfactory ensheathing cell; ON, olfactory nerve; ONL, olfactory nerve layer [Color figure can be viewed at wileyonlinelibrary.com]

All nine pan-OEC-specific markers at E16.5 (i.e., genes expressed by ONL-OECs and mucosal OECs but not by trigeminal Schwann cells) were already expressed by mucosal OECs at E11.5, and by both ONL-OECs and mucosal OECs at E13.0–13.5. These include the three pan-OEC-specific transcription factor genes at E16.5, that is, the Notch transcriptional effector genes *Hey2* (Figure 9a-f) and *HeyL* (Figure 9g-l), plus *Alx3* (Figure 9m-q2). *Alx3* was also strongly expressed by all frontonasal mesenchyme cells at E11.5 (Figure 9n,o), suggesting its expression may be maintained in the frontonasal mesenchyme cells that colonize the developing migratory mass, upregulate *Sox10* expression and develop as OECs (Barraud et al., 2010; Barraud et al., 2013; Forni et al., 2011; Miller et al., 2010).

The four pan-OEC-specific genes at E16.5 that are associated with oligodendrocyte development *(Ptprz1, Ptn, Adgrg1,* and *Sema6a)* were also expressed by mucosal OECs at E11.5 and by both ONL-OECs and mucosal OECs at E13.0–13.5 (Figure S6). *(Ptn* expression was also seen throughout the olfactory epithelium at E11.5 and more weakly at E13.0–13.5, when it was strongly expressed in olfactory mucosal mesenchyme; Figure S6.) The other two pan-OEC-specific genes at E16.5, *Nell2* and *Dpysl3* (*Crmp4*), were likewise expressed at E11.5 by mucosal OECs and at E13.0–13.5 by both ONL-OECs and mucosal OECs (Figure S7). (*Dpysl3* was also expressed strongly by Sox10-negative cells on the olfactory nerve at both stages, likely migrating neurons; Figure S7.)

Taken together, these data—showing that the nine pan-OEC markers at E16.5 are already expressed by mucosal OECs at E11.5— suggest that developing OECs are already distinct from Schwann cells at E11.5. This is a day after Sox10-positive cells are seen in association with the forming migratory mass (Barraud et al., 2013; Forni et al., 2011), and only half a day after the early glial marker Fabp7 is first detectable in the migratory mass (Miller et al., 2010).

Furthermore, *Plp1* and the five novel genes expressed by all OECs and Schwann cells at E16.5 were also expressed by mucosal OECs at E11.5 and by both ONL-OECs and mucosal OECs at E13.0–13.5. These were the transcription factor genes *Zbtb20* and *Sox5* (Figure S8, also including *Plp1),* the integral membrane (mitochondrial or Golgi) protein-encoding gene *Mmd2*, the Src family tyrosine kinase gene *Fyn*, and the membrane-tethered cell-adhesion molecule gene *Cntn1* (Figure S9). (*Cntn1* expression was also seen in Sox10-negative cells on the olfactory nerve at E13.5, which could be migrating neurons; Figure S9).

Overall, these results show that the 14 genes that we identified as being expressed by all OECs at E16.5, whether pan-OEC-specific or also expressed by trigeminal Schwann cells, are already expressed by mucosal OECs at least as early as E11.5, and maintained in both mucosal and ONL-OECs at E13.0–13.5.

### 3.11 ONL-OEC-specific genes at E16.5 are not expressed by mucosal OECs at earlier stages

We next asked whether, like pan-OEC genes at E16.5 (see previous section), genes that are ONL-OEC-specific at E16.5 are expressed by mucosal OECs initially but then turned off before E16.5, or only ever expressed by ONL-OECs. We previously reported that the secreted Wnt inhibitor gene *Frzb,* which at E16.5 is enriched in inner ONL-OECs and almost exclusively restricted to ONL-OECs (with only a few olfactory mucosal OECs and no trigeminal Schwann cells showing any expression; Figure 3b-e; Rich et al., 2018), is not expressed at E11.5, but is expressed at E13.5 by ONL-OECs and a few olfactory mucosal OECs (Rich et al., 2018).

**Figure 10:**
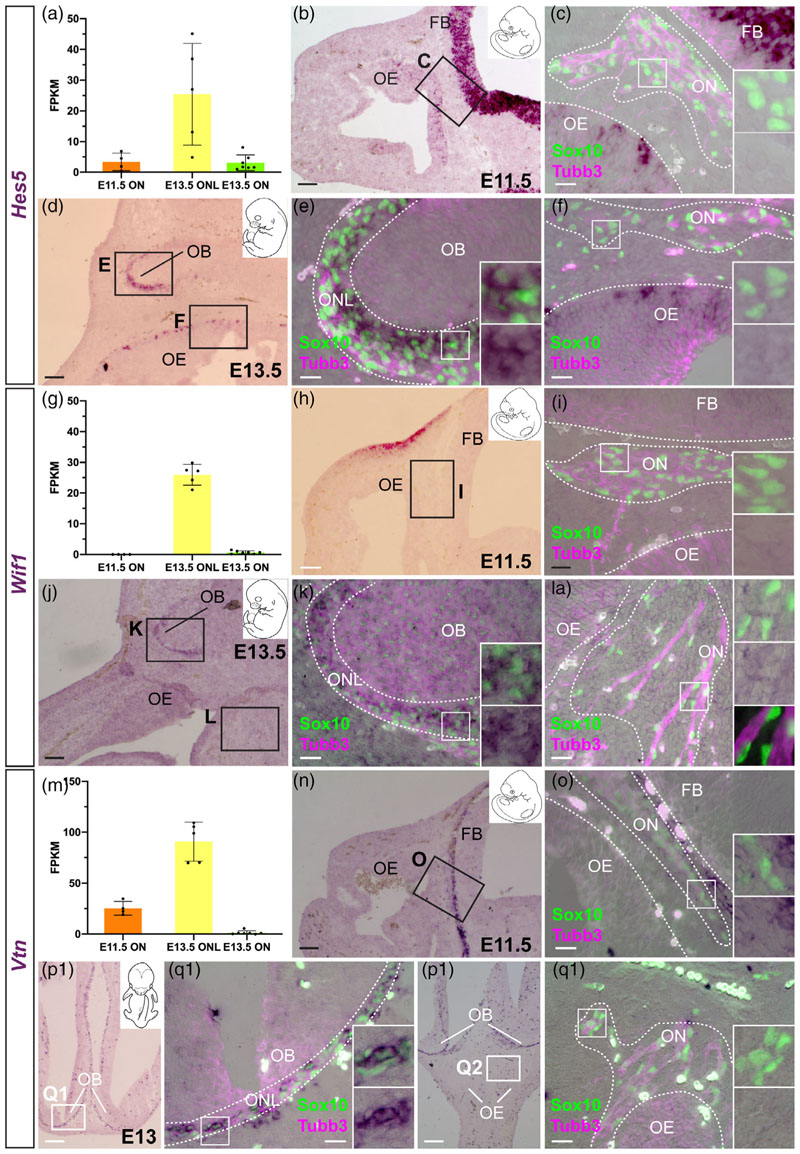
Mucosal OECs at E11.5 and E13.0–13.5 do not express genes that are ONL-OEC-specific at E16.5: *Hes5, Wif1, Vtn.* Bar charts showing mean expression values across all transcriptomes at E11.5 and E13.5 (error bars indicate *SD*), and parasagittal or coronal sections through the mouse olfactory system at E11.5 or E13.0–13.5 (orientation identified by redrawn Theiler stage schematics from the EMAP eMouse Atlas Project, http://www.emouseatlas.org; Richardson et al., 2014), immunostained for Sox10 (green nuclei) to identify OECs and Schwann cells and for Tubb3 (magenta) to identify axons, following in situ hybridization for: (a-f) *Hes5 (n* = 3 at E11.5; *n* = 7 at E13.0–13.5); (g-l) *Wif1 (n* = 2 at E11.5; *n* = 4 at E13.0–13.5); (m–q2) *Vtn (n* = 2 at E11.5; *n* = 3 at E13.0–13.5; panels [p1-q2] show sections from the same embryo). No expression is seen in mucosal OECs at any stage; all three genes are expressed by ONL-OECs at E13.0–13.5 (though *Hes5* expression is restricted to ONL-OECs closest to the olfactory bulb). Scale bars: (b,d,h,j,n,p1,p2) 100 μm; (c,e,f,i,k,l,o,q1,q2) 25 μm. FB, forebrain; FPKM, fragments per kilobase of transcript per million mapped reads; OB, olfactory bulb; OE, olfactory epithelium; OEC, olfactory ensheathing cell; ON, olfactory nerve; ONL, olfactory nerve layer [Color figure can be viewed at wileyonlinelibrary.com]

Like *Frzb*, and in contrast to the pan-OEC markers at E16.5, none of the ONL-OEC-specific genes at E16.5 were expressed by mucosal OECs at earlier stages, although several showed ONL-OEC-specific expression at E13.0–13.5. These were the Notch effector gene *Hes5* (Figure 10a-f), the secreted Wnt inhibitor gene *Wif1* (Figure 10g–l), the secreted glycoprotein gene *Vtn* (Figure 10m–q2), the neuropeptide gene *Npy* (Figure S10a–e2), and the serine protease gene *Prss56* (Figure S10f–j2). This confirms the previous report of ONL-specific *Prss56* expression at E13.5 (Jourdon et al., 2016). In contrast, no OEC expression was seen at E13.0–13.5 for the Wnt transduction inhibitor gene *Nkd2,* the Wnt transduction enhancer gene *Lypd6,* or the lipocalin-type prostaglandin-D synthase gene *Ptgds* (Figure S11), which are all ONL-OEC-specific at E16.5.

The later onset of ONL-OEC-specific gene expression, and the different stage of onset depending on the gene (with E13.0–13.5 expression seen for *Hes5, Wif1, Vtn, Npy,* and *Prss56,* but not for *Nkd2, Lypd6,* or *Ptgds)* suggests that signals from the olfactory bulb (which becomes morphologically distinct from E12.5, as an evagination from the telencephalon; Doucette, 1989; Marin-Padilla & Amieva, 1989; Treloar et al., 2010) likely induce ONL-OEC differentiation, with variation in timing of signals and/or responsiveness of targets.

Furthermore, of the six inner ONL-OEC-enriched genes at E16.5 that also showed ONL-OEC expression at E13.0-E13.5, four were expressed throughout the ONL at this earlier stage. These were *Wif1* (Figure 10k) and *Npy* (Figure S10e1), which were ONL-OEC-specific at E16.5, plus *Plp1* (Figure S8e1) and *Fyn* (Figure S6j1), which were also expressed by mucosal OECs and trigeminal Schwann cells at E16.5. In contrast, expression of *Hes5* (inner ONL-OEC-enriched at E16.5) was restricted at E13.0–13.5 to ONL-OECs within roughly three cell diameters of the olfactory bulb (Figure 10e; compare with, for example, *Wif1* expression throughout the ONL in Figure 10k), while expression of *Prss56* (the only inner ONL-OEC-specific gene at E16.5) was restricted to OECs within roughly two cell diameters of the olfactory bulb (Figure S10j1). This may suggest longer-range diffusible inductive cues for *Wif1* and *Npy*, versus more localized (potentially contact-dependent) inductive cues for *Hes5* and *Prss56.*

Taken together, these data support the hypothesis that ONL-OEC differentiation is a multi-step process, with ONL-OEC-specific genes being induced at different times by signals from the olfactory bulb with differing ranges of action.

### 3.12 The onset of expression of mucosal OEC-specific markers varies

**Figure 11:**
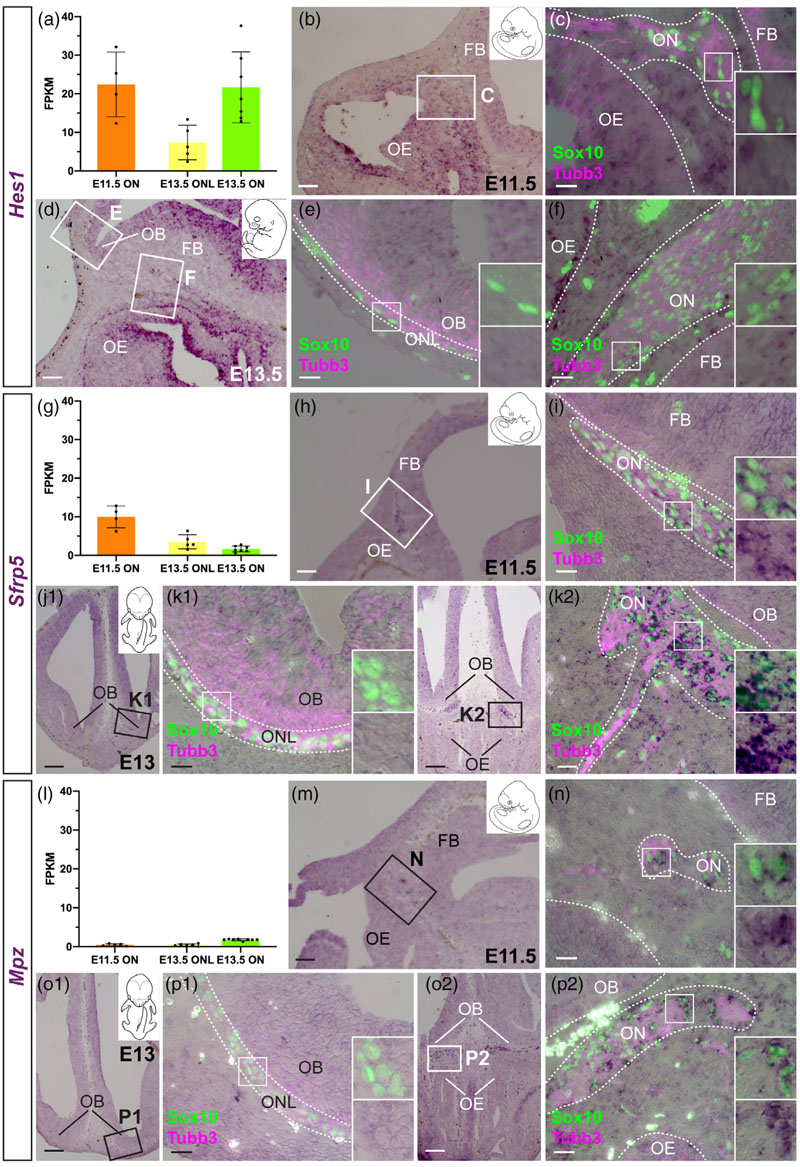
Some genes expressed by mucosal OECs but not ONL-OECs at E16.5 are already expressed at E11.5. Bar charts showing mean expression values across all transcriptomes at E11.5 and E13.5 (error bars indicate *SD*), and parasagittal or coronal sections through the mouse olfactory system at E11.5 or E13.0–13.5 (orientation identified by redrawn Theiler stage schematics from the EMAP eMouse Atlas Project, http://www.emouseatlas.org; Richardson et al., 2014), immunostained for Sox10 (green nuclei) to identify OECs and Schwann cells and for Tubb3 (magenta) to identify axons, following in situ hybridization for: (a–f) *Hes1,* only expressed by some mucosal OECs (*n* = 2 at E11.5; *n* = 4 at E13.0–13.5); (g-k2) *Sfrp5 (n* = 2 at E11.5; *n* = 3 at E13.0–13.5; panels [i1-j2] show sections from the same embryo); (l-p2) *Mpz (n* = 2 at E11.5; *n* = 3 at E13.0–13.5; panels [n1-o2] show sections from the same embryo). All three genes are expressed by mucosal OECs at E11.5 and E13.0–13.5, with no expression seen in ONL-OECs at E13.0–13.5. Scale bars: (b,d,h,j1,j2,m,o1,o2) 100 μm; (c,e,f,i,k1,k2,n,p1,p2) 25 μm. FB, forebrain; FPKM, fragments per kilobase of transcript per million mapped reads; OB, olfactory bulb; OE, olfactory epithelium; OEC, olfactory ensheathing cell; ON, olfactory nerve; ONL, olfactory nerve layer [Color figure can be viewed at wileyonlinelibrary.com]

Of the five genes expressed at E16.5 by mucosal OECs (and trigeminal Schwann cells) but not by ONL-OECs, three were already expressed by mucosal OECs at E11.5 and maintained at E13.0–13.5. These were the Notch effector gene *Hes1,* expressed by some cells only (Figure 11a–f), the secreted Wnt inhibitor gene *Sfrp5* (Figure 11g-k2), and the peripheral myelin-associated gene *Mpz* (Figure 11l-p2). In contrast, the secreted axon-guidance cue gene *Sema3b* was only expressed by mucosal OECs at E13.0–13.5 (Figure S12a–e2), while the secreted Wnt/Bmp inhibitor gene *Sostdc1* was not expressed by OECs at either E11.5 or E13.0–13.5 (Figure S12f–j2). Thus, the expression of different mucosal OEC marker genes is initiated at different times during OEC development, likely reflecting different roles for these genes in the development and/or function of mucosal OECs.

### 3.13 Deletion of *Ptprz1* (but not Ptn) leads to increased expression of OEC-specific genes and disrupts olfactory axon targeting, but not GnRH neuron migration

As described earlier, we identified *Ptprz1,* encoding a receptor-type tyrosine phosphatase, as a pan-OEC-specific marker (expressed by all OECs but not by trigeminal Schwann cells) expressed from at least as early as E11.5. Ptprz1 activity maintains oligodendrocyte precursor cells in an undifferentiated state, whereas binding of the inhibitory ligand pleiotrophin (encoded by *Ptn)* promotes oligodendrocyte differentiation (Kuboyama et al., 2012; Kuboyama et al., 2015; Kuboyama et al., 2016; Tanga et al., 2019). We also identified *Ptn* as a pan-OEC-specific marker, with stronger expression in inner ONL-OECs. Furthermore, *Ptn* expression was seen in the olfactory epithelium (likely including olfactory receptor neurons) at E11.5, though less strongly at E13.0–13.5, and only in scattered cells at E16.5 (Figures S6g-j2 and 2i), in olfactory mucosal mesenchyme at E13.0–13.5 (though not at E11.5) and E16.5 (Figures S6g-j2 and 2i), and in a thin layer of mesenchyme subjacent to the ONL at E16.5 (Figure 2h), but not at E13.0–13.5 (Figure S6i,j1). These expression data suggested the intriguing possibility that Ptprz1 and pleiotrophin may play similar roles in OEC differentiation as in oligodendrocyte differentiation (Kuboyama et al., 2012; Kuboyama et al., 2015; Kuboyama et al., 2016; Tanga et al., 2019).

**Figure 12:**
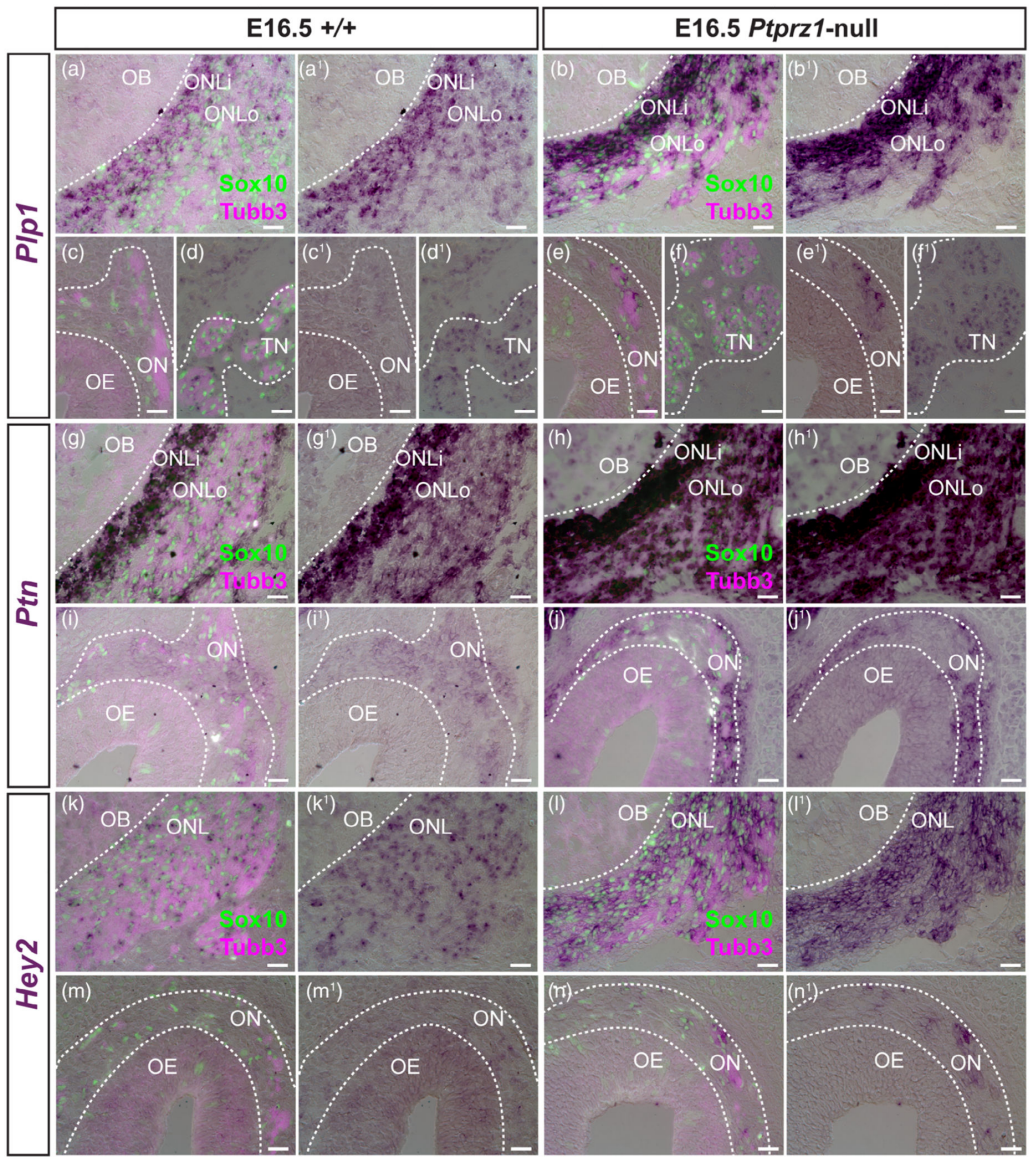
*Ptprz1* deletion leads to increased expression in OECs of the pan-OEC markers *Plp1, Ptn,* and *Hey2.* Coronal sections through the olfactory system of wild-type (*n* =4 from one litter for each gene) or Ptprz1-null *(Ptprz1^lacZ/lacZ^)* littermates at E16.5 (*n* = 3 from one litter for each gene), immunostained for Sox10 (green nuclei) to identify OECs and for Tubb3 (magenta) to identify axons, following in situ hybridization for: (a-f^1^) the pan-OEC (inner ONL-enriched) and trigeminal Schwann cell marker *Plp1,* whose expression is stronger in Ptprz1-null ONL-OECs and mucosal OECs, but not in trigeminal Schwann cells, versus wild-type embryos; (g–j^1^) the pan-OEC-specific (inner ONL-enriched) marker Ptn, whose expression is stronger in Ptprz1-null ONL-OECs and mucosal OECs; (k–m^1^) the pan-OEC-specific marker *Hey2,* whose expression is much stronger in Ptprz1-null ONL-OECs and mucosal OECs. The ISH color reaction was stopped at the same time for each pair of sections shown from the different genotypes. Panels with the superscript 1 (a^1^, b^1^, etc) show the ISH signal alone. Scale bar: 25 μm. ISH, in situ hybridization; OB, olfactory bulb; OE, olfactory epithelium; OEC, olfactory ensheathing cell; ON, olfactory nerve; ONL, olfactory nerve layer; ONLi, inner olfactory nerve layer; ONLo, outer olfactory nerve layer; TN, trigeminal nerve [Color figure can be viewed at wileyonlinelibrary.com]

As a first step to understanding the role of Ptprz1 in OEC development, we analyzed OEC marker gene expression on cryosections of Ptprz1-null *(Ptprz1^lacZ/lacZ^)* embryos (Shintani et al., 1998) and wild-type littermates at E16.5, to determine whether OEC differentiation is affected in the absence of *Ptprz1*. Compared to wild-type littermates (*n* = 4), *Ptprz1*-null embryos (*n* = 3) showed stronger expression of the pan-OEC (inner ONL-enriched) and trigeminal Schwann cell marker gene *Plp1* in both ONL-OECs (inner and outer) and mucosal OECs (which normally express *Ptprz1*), but not trigeminal Schwann cells (Figure 12a–f^1^), which do not express *Ptprz1*. (ISH is not quantitative: slides from wild-type and *Ptprz1*-null embryos, from the same litter, were only compared when ISH was performed in the same round, that is, all slides were treated identically, including the development of the colour reaction and image processing.) Similarly, stronger expression was seen in both ONL-OECs and mucosal OECs for the pan-OEC-specific genes *Ptn* (Figure 12g–j^1^) and *Hey2* (Figure 12k–n^1^).

Furthermore, stronger expression of both the inner ONL-OEC-specific marker *Prss56* and the ONL-OEC-specific (inner ONL-enriched) marker *Npy* was seen in *Ptprz1*-null embryos compared to wild-type littermates (Figure S13a-d^1^). However, there was no obvious difference in the expression of another ONL-OEC-specific (inner ONL-enriched) marker, *Nkd2* (Figure S13e-f^1^).

Overall, deletion of *Ptprz1* led to noticeably stronger expression by ISH of all three pan-OEC genes examined (in both ONL-OECs and mucosal OECs), and of two out of three ONL-OEC-specific genes examined. The increased expression of *Plp1* in *Ptprz1*-null embryos is restricted to OECs, which normally express *Ptprz1*, and is not seen in trigeminal Schwann cells, which do not express *Ptprz1*. This suggests that Ptprz1 potentially acts as a brake on OEC differentiation, as it does for oligodendrocytes (Kuboyama et al., 2012; Kuboyama et al., 2015; Kuboyama et al., 2016; Tanga et al., 2019).

We next aimed to determine whether *Ptprz1* deletion affects OEC function. In *Sox10*-null embryos, defective OEC differentiation disrupts both olfactory axon targeting and GnRH neuron entry into the forebrain (Barraud et al., 2013; Pingault et al., 2013), whereas in embryos null for the inner ONL-OEC-enriched gene *Frzb*, olfactory axon targeting is disrupted, but not GnRH neuron entry into the fore-brain (Rich et al., 2018). For these experiments, we used the maturation of olfactory receptor neurons as an indirect but quantifiable proxy for olfactory axon targeting (Barraud et al., 2013; Rich et al., 2018), because expression by these neurons of the maturation marker Omp correlates with synaptogenesis (Farbman & Margolis, 1980; Monti Graziadei et al., 1980).

**Figure 13:**
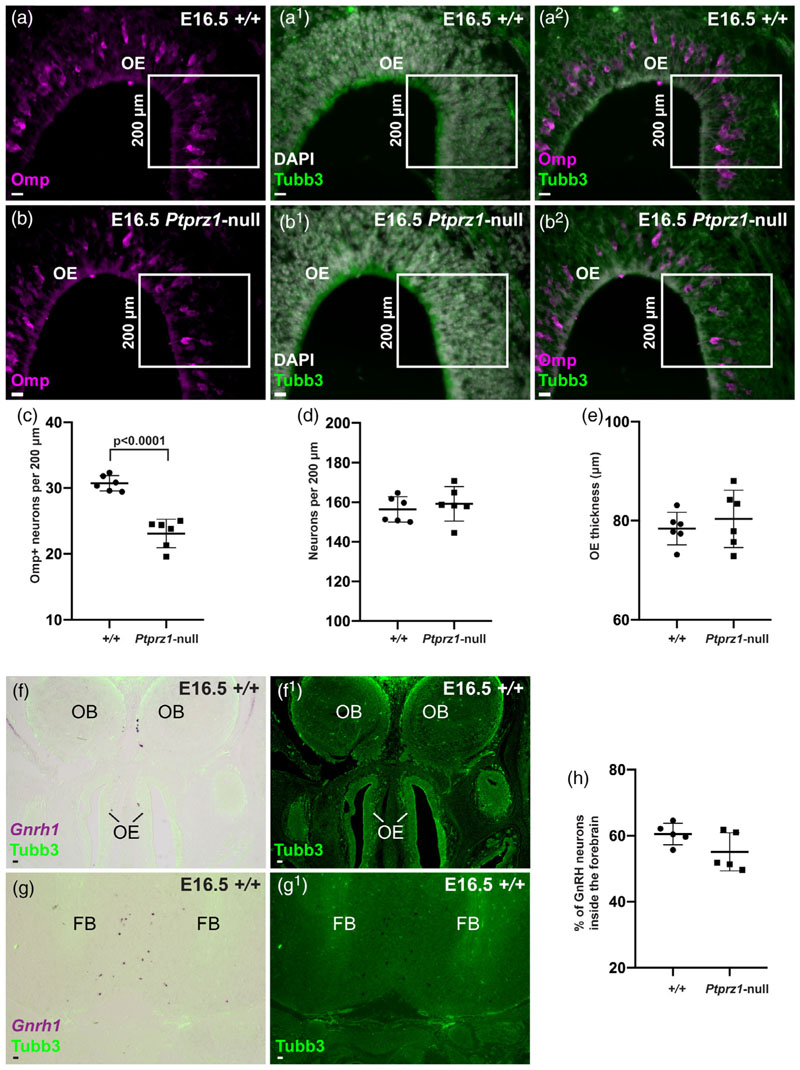
*Ptprz1* deletion disrupts olfactory receptor neuron maturation but not GnRH neuron entry into the forebrain. (a–b^2^) Example coronal sections through a region of dorsal olfactory epithelium at E16.5 from a wild-type embryo (a-a^2^) and a Ptprz1-null *(Ptprz1^lacZ/lacZ^)* littermate (b-b^2^), immunostained for the maturation marker Omp and the general neuronal/axonal marker Tubb3 and counter-stained with DAPI. A 200 μm length of olfactory epithelium was selected (white box) on both left and right sides of at least three coronal sections per embryo, within which were counted all Omp-positive (mature) neurons (magenta dots; a,b) and all Tubb3-positive neurons (green dots; a^1^,b^1^), and the width measured in three places. (c) Scatter plot (bars indicate mean and SD) showing the mean number per embryo of Omp-positive (mature) neurons per 200 μm of olfactory epithelium. This is significantly higher for wild-type embryos (mean 30.7 ± *SD* 1.2%; *n* = 6 from one litter) than Ptprz1-null littermates (mean 23.1 ± 2.2; *n* = 6; *p* < 0.0001; unpaired two-tailed Student’s t-test; t = 7.59; 10 degrees of freedom). (d) Scatter plot (bars indicate mean and SD) showing the mean number per embryo of neurons per 200 μm of olfactory epithelium. There is no significant difference between wild-type embryos (mean 156.4 ± 6.4; *n* = 6) and Ptprz1-null littermates (mean 159.2 ± 8.7; *n* = 6; *p* = 0.54; unpaired two-tailed Student’s *t* test). (e) Scatter plot (bars indicate mean and SD) showing the mean thickness per embryo of the olfactory epithelium. There is no significant difference between wild-type embryos (mean 78.4 ± 3.3 μm; *n* = 6) and Ptprz1-null littermates (mean 80.4 ± 5.8 μm; *n* = 6; *p* = 0.49; unpaired two-tailed Student’s *t* test). (f–g^1^) Example coronal sections at more rostral (f,f^1^) and more caudal levels (g,g^1^) of a wild-type embryo at E16.5, immunostained for Tubb3 following in situ hybridization for the GnRH neuron marker *Gnrh1.* (h) Scatter plot (bars indicate mean and SD) showing the percentage of GnRH neurons (at least 149 counted per embryo) found inside the brain for wild-type embryos (mean 60.5 ± 3.2%; *n* = 5 from one litter) versus Ptprz1-null littermates (mean 55.1 ± 5.8%; *n* = 5). The difference between the means is not significant (p = 0.11; unpaired two-tailed Student’s *t* test). Scale bars: (a–b^2^) 25 μm; (f-g^1^) 100 μm. FB, forebrain; OB, olfactory bulb; OE, olfactory epithelium [Color figure can be viewed at wileyonlinelibrary.com]

To compare olfactory receptor neuron maturation between *Ptprz1*-null embryos and wild-type littermates, we randomly selected a 200-^m span of dorsal olfactory epithelium on both left and right sides of each of three to six coronal sections per embryo, immuno-stained for Omp and the neuronal marker Tubb3, and counter-stained for DAPI to label nuclei. Within each 200-μm span, we counted all Omp-positive (mature) neurons and all neurons (Figure 13a-b^2^), and measured the thickness of the epithelium at three different points. The mean number per embryo of Omp-positive (mature) olfactory receptor neurons per 200 μm of epithelium was significantly lower in Ptprz1-null embryos than in wild-type littermates (Figure 13c), whereas there was no significant difference in the mean number of neurons (Figure 13d) or the mean thickness of the epithelium (Figure 13e). Overall, these data suggest that *Ptprz1* deletion specifically disrupts the maturation of olfactory receptor neurons, which in turn suggests a defect in olfactory axon targeting. In contrast, we found no significant difference in the mean proportion of GnRH neu-rons inside the forebrain in *Ptprz1*-null embryos versus wild-type lit-termates (Figure 13f-h).

To test whether pleiotrophin, the inhibitory Ptprz1 ligand, might promote OEC differentiation as well as oligodendrocyte differentiation (Kuboyama et al., 2012; Kuboyama et al., 2015; Kuboyama et al., 2016; Tanga et al., 2019), we undertook a similar analysis in *Ptn*-null embryos (Muramatsu et al., 2006) at E16.5. We found no obvious differences between wild-type and *Ptn*-null littermates in the expression of pan-OEC markers (Figure S14a–h^1^) or ONL-OEC-specific markers (Figure S14i–n^1^), nor between wild-type, heterozygous or *Ptn*-null littermates in terms of olfactory receptor neuron maturation (as a proxy for olfactory axon targeting; Figure S15a-c) or the proportion of GnRH neurons entering the forebrain (Figure S15d). Despite the lack of any apparent effect of *Ptn* deletion, we cannot rule out an interaction between pleiotrophin and Ptprz1 during OEC development because there could be redundancy with other ligands for Ptprz1. For example, *Cntn1* (encoding a membrane-tethered cell-adhesion molecule that binds Ptprz, promoting oligodendrocyte differentiation (Lamprianou et al., 2011), is expressed by OECs and potentially also by olfactory axons (Figure S4f-i).

Overall, our results suggest that Ptprz1 may act as a brake on OEC differentiation, and support a requirement for Ptprz1 for normal olfactory axon targeting.

## 4 Discussion

Here, we took an unbiased differential RNA-seq approach to identify molecular differences between developing OEC subpopulations and Schwann cells in vivo, aiming to shed light on OEC development and diversification, how this differs from Schwann cell development, and the molecular basis for interactions between OECs and olfactory axons or migrating GnRH neurons. We used laser microdissection to isolate olfactory nerve regions (from the olfactory mucosa, subjacent to the olfactory epithelium, and from the ONL, around the olfactory bulb) and trigeminal nerve from the same cryosections of E16.5 mouse embryos, and compared their transcriptomes to identify candidate genes differentially expressed between mucosal OECs, ONL-OECs, and/or trigeminal Schwann cells. Validation of top-ranked candidate genes by in situ hybridization (focusing mainly on genes encoding transcription factors, receptors, secreted factors and other signalling pathway members) identified 25 novel markers for developing mouse OECs in vivo, of which 15 were expressed by ONL-OECs and/or mucosal OECs but not by trigeminal Schwann cells, while 11 distinguished mucosal OECs from ONL-OECs (summarized in File S5 and Figure S5). Our expression data suggest that OECs are distinct from Schwann cells as soon as they emerge, that OEC subpopulation diversification involves signals from the olfactory bulb, and that Wnt pathway activity must be tightly controlled in the inner ONL and likely also in the olfactory mucosa for normal OEC function (further supporting our recent study showing that the inner ONL-OEC-enriched secreted Wnt inhibitor gene *Frzb* is required for normal olfactory axon targeting; Rich et al., 2018). We identified differential expression between ONL-OECs and mucosal OECs of genes encoding proteins involved in axon guidance, neurite outgrowth and/or cell migration, supporting different interactions between OEC subpopulations and olfactory axons (Ekberg et al., 2012; Schwarting et al., 2000; Schwarting, Raitcheva, Crandall, Burkhardt, & Puschel, 2004), and potentially also with migrating GnRH neurons. Finally, we identified intriguing molecular parallels with oligodendrocyte development, including expression of the receptor-like tyrosine phosphatase Ptprz1 (whose activity blocks oligodendrocyte differentiation; Kuboyama et al., 2012; Kuboyama et al., 2015; Kuboyama et al., 2016; Tanga et al., 2019), which we found may play a similar role in OEC development and is required for normal olfactory axon targeting.

### 4.1 Developing OECs are distinct from Schwann cells at all stages in the mouse

We identified nine novel genes expressed by all OECs (i.e., mucosal OECs and ONL-OECs) that distinguish them in vivo from trigeminal Schwann cells at E16.5: the transcription factor genes *Hey2*, *HeyL*, and *Alx3*; the receptor genes *Adgrg1* (*Gpr56*) and *Ptprz1*; the ligand genes *Ptn*, *Sema6a*, and *Nell2*; and the cytosolic process-outgrowth/ cell migration-inhibitor gene *Dpysl3* (*Crmp4*). All these pan-OEC-specific genes were already expressed in OECs from at least as early as E11.5 (the earliest stage examined), which is only 12 hours after developing OECs in the migratory mass upregulate the early glial marker Fabp7 (Blbp), at E11 (Miller et al., 2010).

One of the pan-OEC-specific genes, the homeobox transcription factor gene *Alx3,* was also expressed throughout the frontonasal mesenchyme subjacent to the olfactory epithelium at E11.5. Strong *Alx3* expression in frontonasal mesenchyme was previously reported as early as E9.5 (ten Berge et al., 1998), when the olfactory placodes are morphologically detectable but the migratory mass of emigrating neurons and olfactory axons has not yet formed (Miller et al., 2010). Taken together with pan-OEC-specific *Alx3* expression at all stages examined (including E11.5), this suggests that *Alx3* is not induced in differentiating OECs, but maintained throughout OEC development from its earlier expression in neural crest-derived frontonasal mesenchyme cells. These cells are plastic: for example, we previously showed that constitutive activation of Notch/Rbpjsignalling in chicken frontonasal mesenchyme cells converts them to a perivascular fate (Miller et al., 2017). Some of these cells colonize the migratory mass as it forms and upregulate *Sox10* at least as early as E10.5, forming OEC precursors (Barraud et al., 2010; Barraud et al., 2013; Forni et al., 2011). Although *Sox10* is expressed by neural crest cells emigrating from all axial levels of the neural tube, its expression is lost in all neural crest derivatives except neuroglial precursors and melanocytes, including the frontonasal mesenchyme (Herbarth et al., 1998; Kuhlbrodt, Herbarth, Sock, Hermans-Borgmeyer, & Wegner, 1998; Pusch et al., 1998), well before the olfactory placodes are detectable at E9.5 and the migratory mass starts to form at E10.0 (Miller et al., 2010).

*Alx3* has primarily been studied in the context of craniofacial skeletal development: homozygous recessive mutations in humans cause frontonasal dysplasia (Twigg et al., 2009). Although *Alx3*-null mice are viable and fertile, this is due to redundancy with *Alx4*, which has a similar expression pattern: double mutants die at birth with severe nasal clefting and skull defects (Beverdam, Brouwer, Reijnen, Korving, & Meijlink, 2001). However, the maintenance of *Alx3* in all OECs, even at E16.5, suggests a role in OEC development rather than simply inheritance of *Alx3* expression from the frontonasal mesenchyme precursors of OECs. Alx3 directly represses the melanocyte-specifying transcription factor gene *Mitf* (Mallarino et al., 2016), which is also a direct Sox10 target gene (Bondurand et al., 2000; Lang & Epstein, 2003). Mitf and Sox10 co-operate to induce a melanocyte fate (Marathe et al., 2017). Some Schwann cell precursors give rise to melanocytes during normal development (Adameyko et al., 2009; Adameyko et al., 2012; Nitzan, Pfaltzgraff, Labosky, & Kalcheim, 2013), as well as to multiple other derivatives (reviewed by Furlan & Adameyko, 2018; Jessen & Mirsky, 2019). The glial lineage of Schwann cell precursors is maintained by Sox2, which directly represses *Mitf*, preventing their specification as melanocytes (Adameyko et al., 2012). Sox2 is expressed by developing OECs in chicken, but not mouse (Miller, Perera, Benito, Stott, & Baker, 2016; also see, e.g., Beites, Kawauchi, Crocker, & Calof, 2005; Forni et al., 2011). Intriguingly, the *Alx3* gene has been lost in chicken (McGonnell et al., 2011). Taken together, this suggests the hypothesis that the direct *Mitf*-repressor Alx3 (Mallarino et al., 2016) may substitute for Sox2 in repressing *Mitf* in developing mouse OECs, maintaining their glial lineage.

Furthermore, although *Alx3*-null mice have no obvious phenotype, double *Alx3*/*Alx4* mutants have a discrete patch of apoptotic cells immediately subjacent to the olfactory placodes at E10.0 (Beverdam et al., 2001), precisely where the migratory mass is forming (Miller et al., 2010). This was interpreted as somehow being important for the later craniofacial phenotype of the double mutants (Beverdam et al., 2001). However, *Alx4* has a similar expression level to *Alx3* in the “inner ONL” transcriptome at E16.5 (File S1), which is least likely to have any mesenchymal contamination. This suggests that *Alx4* is likely also expressed by developing OECs. It is therefore tempting to speculate that the patch of apoptotic cells subjacent to each olfactory placode in *Alx3/Alx4* double mutants (Beverdam et al., 2001) represents dying OEC precursors, suggesting the hypothesis (which is beyond the scope of this work to test) that *Alx3* and *Alx4* are redundantly required for the survival of those frontonasal mesenchyme cells that colonize the olfactory nerve and upregulate *Sox10* to form OEC precursors.

Overall, our data suggest that the mouse OEC lineage is molecularly distinct from developing Schwann cells as soon as it forms.

Interestingly, several of the embryonic mouse OEC markers identified here were reported (though not validated) as expressed by adult rat ONL-OECs in a microarray study that compared ONL-OECs cultured for 3.5 weeks, sciatic-nerve Schwann cells cultured for two weeks, and “native” ONL-OECs acutely dissected from the ONL (Franssen et al., 2008). Pairwise microarray comparisons between the different populations identified ONL-OEC-specific *Hes5* and Vtn, pan-OEC-specific *Ptprz1* and *Ptn*, and the pan-OEC and Schwann cell marker *Plp1,* as being enriched in native OECs over cultured OECs (Franssen et al., 2008). Similarly, OEC-specific *Nell2* was enriched in cultured ONL-OECs over cultured Schwann cells, although OEC-specific *Dpysl3* was enriched in cultured Schwann cells over cultured ONL-OECs (Franssen et al., 2008). Taken together, the reported (albeit not validated) expression in adult rat ONL-OECs (whether acutely isolated or after 3.5 weeks in culture; Franssen et al., 2008) of several of the embryonic mouse OEC marker genes identified in this study suggests that at least some of these markers may be useful for the identification of adult OECs in culture.

### 4.2 OEC subpopulation diversification likely requires signals from adjacent tissues

Our results suggest, perhaps unsurprisingly, that proximity to the developing olfactory bulb, which is morphologically distinct at E12.5 (Doucette, 1989; Marin-Padilla & Amieva, 1989; Treloar et al., 2010), is important for the diversification of OEC subpopulations. We identified five mucosal OEC-specific genes at E16.5 (all shared with trigeminal Schwann cells, but not expressed by ONL-OECs) of which three (the Notch effector *Hes1*, expressed by only some OECs, plus the myelin protein gene *Mpz* and the secreted Wnt inhibitor gene *Sfrp5*) were already expressed at least as early as E11.5, when only mucosal OECs are present. Their absence in ONL-OECs at E13.0–13.5, as well as at E16.5, suggests they may be repressed in developing ONL-OECs by diffusible signals emanating from the olfactory bulb. The other two mucosal OEC-specific genes (the secreted semaphorin gene *Sema3b* and the secreted dual Wnt/Bmp inhibitor *Sostdc1)* were absent at E11.5 but expressed by E13.0–13.5. Their expression might be induced at a later stage by cues upregulated in the mesenchyme adjacent to olfactory nerve fascicles. We identified several genes with highly localized expression in mesenchyme surrounding olfactory and/or trigeminal nerve fascicles (and/or the ONL; Figure S2), providing indirect support for the feasibility of mesenchyme-derived cues affecting OEC differentiation.

Conversely, signals from the developing olfactory bulb likely induce and maintain ONL-OEC-specific genes. In addition to the previously characterized inner ONL-OEC-enriched neuropeptide gene *Npy* (Au et al., 2002; Barraud et al., 2013) and the inner ONL-OEC-specific serine protease gene *Prss56* (Jourdon et al., 2016), we identified six novel ONL-OEC-specific genes at E16.5: four with enriched expression in inner ONL-OECs (the Notch-effector gene *Hes5,* the cell-intrinsic Wnt pathway modulator genes *Lypd6* and *Nkd2*, and the secreted Wnt inhibitor gene *Wif1)* plus two genes encoding secreted proteins *(Ptgds, Vtn). Prss56* and *Hes5* were expressed at E13.0–13.5 only by OECs within roughly 2-3 cell diameters of the olfactory bulb, suggesting the possibility of induction by contact-dependent or minimally diffusible signals. The Notch target gene *Hes5,* at least, could be induced directly in OECs nearest the olfactory bulb by Notch ligands expressed in the outer olfactory bulb: mucosal OECs and ONL-OECs express *Notch1* at E14.5 (Miller et al., 2016), while expres-sion of the Notch ligand gene *Jag1* was reported in the superficial mantle layer of the olfactory bulb at least as early as E14.0, and in the nascent glomerular layer/external plexiform layer (i.e., immediately beneath the ONL), as well as in the deeper mitral cell layer, at E17.5 (Williams et al., 2007). Expression of the Notch ligand gene *Jag2* was also reported deep to the ONL at E16.5 (Miller et al., 2016).

In contrast, *Npy*, *Wif1*, and *Vtn* were expressed by OECs throughout the ONL at E13.0–13.5, suggesting induction by diffusible signals from the olfactory bulb. Finally, *Lypd6, Nkd2,* and *Ptgds* were not expressed at E13.0–13.5, suggesting that these genes are induced in ONL-OECs by different signals at a later stage.

### 4.3 OEC subpopulations differentially express cues that guide axons, promote neurite outgrowth, and promote cell migration

Defects in both olfactory axon targeting and GnRH neuron entry into the forebrain result from disrupted OEC differentiation in Sox10-null mice (Barraud et al., 2013; Pingault et al., 2013). We identified several genes encoding secreted or membrane-associated proteins with roles in axon guidance, neurite outgrowth and/or cell migration that were expressed by OECs at E16.5. The secreted axon repellant gene *Nell2* (Jaworski et al., 2015) and the transmembrane semaphorin gene *Sema6a* (Jongbloets & Pasterkamp, 2014) were pan-OEC-specific (i.e., expressed by all OECs but not by trigeminal Schwann cells). However, other genes in this category were differentially expressed by OEC subpopulations, consistent with different interactions of ONL-OECs and mucosal OECs with olfactory axons (Ekberg et al., 2012) and potentially also suggesting different interactions with migrating GnRH neurons. ONL-OECs (but not mucosal OECs or trigeminal Schwann cells) expressed *Vtn*, encoding the secreted extracellular matrix-associated glycoprotein, vitronectin, which promotes neurite extension from multiple neuronal types (Plantman, 2013) and the migration of cerebellar granule neurons (Katic et al., 2014; Plantman, 2013), as well as *Ptgds*, encoding lipocalin-type prostaglandin-D synthase, which promotes astrocyte migration (Lee et al., 2012). It is also possible that OEC-secreted cues might act in a paracrine manner to affect the migration of OECs themselves.

Conversely, mucosal OECs (and trigeminal Schwann cells) but not ONL-OECs expressed the secreted semaphorin gene *Sema3b.* The related protein Sema3a is secreted from a subset of OECs in the outermost ONL (found medially at rostral levels of the olfactory bulb and ventrally at caudal levels), specifically repelling the subset of olfactory axons expressing the Sema3a coreceptor, neuropilin 1 (Nrp1) (Schwarting et al., 2000; Schwarting et al., 2004). Sema3a is also required for GnRH neuron migration, acting via both Nrp1 and Nrp2 (Cariboni, Hickok, et al., 2007; Cariboni et al., 2011). Nrp2 is a coreceptor for Sema3b (Falk et al., 2005; Pignata, Ducuing, & Castellani, 2016), raising the possibility that mucosal OEC secretion of Sema3b may also contribute to GnRH neuron migration.

### 4.4 Tight regulation of Wnt pathway activity seems to be important in the inner ONL

We recently reported that the secreted Wnt inhibitor gene *Frzb (Sfrp3)* is expressed by inner ONL-OECs (and at lower levels by outer ONL-OECs and some mucosal OECs) and that its deletion disrupts olfactory axon targeting (Rich et al., 2018) (for which we used olfactory receptor neuron maturation as a quantifiable proxy, as in the current study and in previous work; Barraud et al., 2013). Here, we identified three other inner ONL-OEC-enriched Wnt pathway modulating genes (all ONL-OEC-specific, that is, not expressed by mucosal OECs or trigeminal Schwann cells): *Wif1*, encoding a secreted protein that binds Wnts, preventing them from binding their receptors (Poggi, Casarosa, & Carl, 2018); *Nkd2*, encoding a myristoylated protein that destabilizes the essential Wnt signalling component Dishevelled (Mlodzik, 2016; Sharma, Castro-Piedras, Simmons, & Pruitt, 2018) at the plasma membrane, cell-autonomously blocking Wnt pathway transduction (Hu et al., 2010); and *Lypd6,* itself a Wnt transcriptional target gene, encoding a plasma membrane-associated protein that interacts with the Wnt receptor Frizzled8 and coreceptor Lrp6 in membrane rafts and acts as a cell-autonomous feedback enhancer of Wnt/beta-catenin transduction (Özhan et al., 2013).

The secreted Wnt inhibitors Frzb and Wif1 could act to inhibit Wnt signalling directly in olfactory axons in the inner ONL, given that olfactory receptor neurons express Dishevelled family members and different Frizzled receptors, as well as Wnts (Rodriguez-Gil & Greer, 2008; Rodriguez-Gil, Hu, & Greer, 2013). However, ONL-OEC-secreted Wnt inhibitors could also block Wnt signalling in other ONL-OECs. Their expression (most strongly in inner ONL-OECs) of both the cell-autonomous Wnt transduction inhibitor gene *Nkd2* and the cell-autonomous Wnt/beta-catenin feedback enhancer *Lypd6* supports the hypothesis that tight control of Wnt signalling activity is essential for ONL-OECs, especially inner ONL-OECs.

We also found that mucosal OECs (and trigeminal Schwann cells), but not ONL-OECs, expressed two different secreted Wnt pathway inhibitor genes: *Sfrp5*, encoding a Wnt-binding protein (Cruciat & Niehrs, 2013), and *Sostdc1 (Wise),* encoding a cystine knot-containing protein that competes with Wnts for binding to the Wnt coreceptors Lrp6 and Lrp4, and also blocks Bmp signalling by binding Bmps (Cruciat & Niehrs, 2013; Lintern, Guidato, Rowe, Saldanha, & Itasaki, 2009). Thus, Wnt pathway inhibition also seems to be important outside the ONL, whether for OECs themselves, for olfactory axons and/or for GnRH neurons, with which OECs are closely associated throughout their migration (Geller et al., 2013).

### 4.5 Molecular similarities with boundary cap cells suggest a novel role for inner ONL-OECs

Several genes expressed by inner ONL-OECs were previously identified as specific markers for boundary cap cells (a molecularly distinct population of neural crest-derived glial cells found at embryonic nerve roots; Radomska & Topilko, 2017) in a microarray analysis comparing the transcriptome profiles of mouse neural crest cells, Schwann cell precursors, and boundary cap cells (Coulpier et al., 2009). These are the previously described inner ONL-OEC-specific serine protease gene *Prss56* (Jourdon et al., 2016), the ONL-OEC-specific (and inner ONL-OEC-enriched) secreted Wnt inhibitor gene *Wif1*, plus the pan-OEC-specific transmembrane semaphorin gene *Sema6a* and the Notch effector genes *Hey2* and *HeyL*. Furthermore, immunoreactivity for the inner ONL-OEC-enriched neuropeptide Npy (Au et al., 2002; Barraud et al., 2013) was reported in embryonic nerve roots as well as OECs in rats (Ubink & Hökfelt, 2000), which was later suggested to represent expression in boundary cap cells (Ubink, Calza, & Hökfelt, 2003).

Boundary cap cells give rise to satellite glia and a subset of sensory neurons in dorsal root ganglia, as well as all the Schwann cell precursors in the dorsal and ventral roots and their derivatives (Gresset et al., 2015; Maro et al., 2004). They also prevent motor neuron cell bodies from exiting the embryonic spinal cord (Vermeren et al., 2003), and, together with Schwann cells, prevent oligodendrocytes and astrocytes from exiting the embryonic CNS (Coulpier et al., 2010; Kucenas, Wang, Knapik, & Appel, 2009), for which a non-myelination-related function of the zinc finger transcription factor Egr2 (Krox20) is required (Coulpier et al., 2010).

OECs were previously suggested to contribute to the glia limitans of the olfactory system (Doucette, 1991), but a recent report suggests that this interface is instead entirely astrocytic, as elsewhere in the CNS, suggesting that all OECs are peripheral glia rather than being part of the CNS (Nazareth et al., 2019). The molecular similarities between boundary cap cells (Coulpier et al., 2009) and inner ONL-OECs, that is, shared expression of *Prss56* (Jourdon et al., 2016) and most likely also *Npy* (Ubink et al., 2003; Ubink & Hökfelt, 2000), plus *Wif1, Sema6a, Hey2* and *HeyL* (this work), lead us to speculate that one function of inner ONL-OECs may be to prevent CNS glia from exiting the olfactory bulb, like boundary cap cells at all other regions where peripheral nerves interface with the CNS (Coulpier et al., 2010). *Egr2* is expressed at negligible levels (mean FPKM levels below 1.0) in our embryonic olfactory nerve transcriptomes (File S1), suggesting that developing OECs do not express this gene. Nevertheless, shared expression with boundary cap cells raises the possibility that the inner ONL-OEC-secreted proteins Prss56, Wif1, and Npy are involved in preventing astrocytes, oligodendrocytes, and perhaps also neurons, from exiting the olfactory bulb, rather than being important for olfactory axon targeting.

### 4.6 Parallels with oligodendrocytes suggest roles for some genes in OEC migration or process extension

One of the pan-OEC-specific genes that we identified encodes the transmembrane semaphorin Sema6a, which can also transduce signals into the expressing cell (Perez-Branguli et al., 2016). Sema6a expression in oligodendrocytes promotes the expression of myelin genes and myelination (Bernard et al., 2012), but its expression in oligodendrocyte precursor cells seems to promote their migration in response to PlexinA4 presented by neighbouring cells (Okada & Tomooka, 2012; Okada & Tomooka, 2013). Sema6a expressed by OECs could similarly be involved in cell-autonomously promoting their migration.

Another gene expressed by all OECs (though enriched in ONL-OECs over both mucosal OECs and trigeminal Schwann cells, with particularly strong expression in inner ONL-OECs) and oligodendrocyte precursor cells is the proteolipid protein gene *Plp1* (Dickinson et al., 1996; Fanarraga et al., 1996; Harlow, Saul, Culp, Vesely, & Macklin, 2014). Our *Plp1* riboprobe does not distinguish between the longer *Plp1* splice isoform and the shorter (and evolutionarily more ancient) *Dm20* splice variant (Griffiths et al., 1998), whose expression was previously reported in ONL-OECs from E14 (Dickinson et al., 1997), as well as in satellite glia and nonmyelinating Schwann cells (Griffiths et al., 1995). *Plp1* expression was also recently reported in enteric glia (Rao et al., 2015). The expression of Dm20/Plp1 in oli-godendrocyte precursor cells (which depends on Sox10; Stolt et al., 2002) and nonmyelinating glia suggests that these proteins must also play nonstructural roles in glia. Indeed, in oligodendrocyte precursor cells, Plp1 forms a complex with α_v_ integrin and is required for their migration in response to glutamate (Gudz, Komuro, & Macklin, 2006; Gudz, Schneider, Haas, & Macklin, 2002). Hence, like Sema6a, Plp1 could act to promote OEC migration.

Conversely, another of the pan-OEC-specific genes that we identified, *Dpysl3 (Crmp4),* acts cell-autonomously to inhibit cell migration (Rosslenbroich et al., 2005). *Dpysl3* encodes a microtubule-stabilising phosphoprotein that bundles F-actin, forms a complex with RhoA, and blocks both cell migration and neurite outgrowth (reviewed by Nagai et al., 2017). The protein encoded by the founding member of this five-gene vertebrate family, Dpysl2 (Crmp2), was identified as mediating axon growth-cone collapse in response to Sema3a, itself previously known as collapsin, hence the original nomenclature of collapsin response mediator protein (Goshima, Nakamura, Strittmatter, & Strittmatter, 1995). All five family members are expressed in postnatal oligodendrocytes, in which process outgrowth is inhibited by Sema3a acting via Nrp1, Dypsl2, and Dypsl5 (Crmp5) (Ricard et al., 2000; Ricard et al., 2001). In contrast, Dpysl5 seems to act in immature and non-myelinating Schwann cells to promote process outgrowth and branching (Camdessanché et al., 2012). Taken together, this suggests that Dpysl3 (Crmp4) may be involved in regulating OEC process outgrowth, rather than (or in addition to) regulating OEC migration.

### 4.7 Parallels with oligodendrocytes suggest that combined Sox5 and Hes5 expression by ONL-OECS could help block the expression of myelin genes

OECs are nonmyelinating in situ but will myelinate larger-diameter axons both in vitro and in vivo, forming myelin that includes both Mpz (e.g., Franklin, 2003) and Mbp (e.g., Babiarz et al., 2011; Devon & Doucette, 1992). We found that mucosal OECs (and trigeminal Schwann cells) but not ONL-OECs expressed *Mpz* at all stages examined, whereas only trigeminal Schwann cells expressed *Mbp*. Both *Mpz* and *Mbp* are direct Sox10 target genes (see Jacob, 2015; Wittstatt, Reiprich, & Küspert, 2019).

Given that embryonic ONL-OECs do not express either *Mpz* or Mbp, it was interesting to find ONL-OEC-specific expression of the Notch transcriptional effector gene *Hes5,* and ONL-OEC enrichment (over mucosal OECs and trigeminal Schwann cells) of the *SoxD* family transcription factor gene *Sox5*. All three SoxE family members (Sox8, Sox9, Sox10) are important for oligodendrocyte development (see Sock & Wegner, 2019; Wittstatt et al., 2019), but Sox10 is essential for terminal differentiation and activation of myelin gene expression, including *Mbp* (Stolt et al., 2002). Hes5 directly inhibits the expression of myelin genes in oligodendrocyte precursor cells, including *Mbp*, and also binds Sox10 protein, preventing Sox10 from binding its target sites in *Mbp* regulatory regions (Liu et al., 2006). The SoxD family members Sox5 and Sox6, which lack a transactivation domain, compete with Sox10 and other SoxE family proteins (i.e., Sox8 and Sox9) for DNA-binding sites on the *Mpz* and *Mbp* promoters (Stolt et al., 2006). Taken together, it is possible that Hes5 and Sox5 coexpression in developing ONL-OECs may help to prevent them from expressing the myelin genes *Mpz* and Mbp.

### 4.8 The pan-OEC-specific receptor tyrosine phosphatase Ptprz1, which blocks oligodendrocyte differentiation, is required for normal olfactory axon targeting

Two of the pan-OEC-specific genes that we identified encode transmembrane receptors whose activity maintains oligodendrocyte precursor cells in an undifferentiated state. *Adgrg1* encodes the adhesion-class G protein-coupled receptor Adgrg1 (Gpr56), which promotes oli-godendrocyte precursor cell proliferation and blocks oligodendrocyte differentiation (Ackerman et al., 2015; Giera et al., 2015; Giera et al., 2018). *Ptprz1* encodes a receptor-type tyrosine phosphatase, whose inactivation in oligodendrocyte precursor cells via binding of its inhibitory ligand pleiotrophin (a secreted heparin-binding protein encoded by *Ptn*, which we also identified as pan-OEC specific) results in increased phosphorylation of target proteins and oligodendrocyte differentiation, myelination and remyelination (Kuboyama et al., 2012; Kuboyama et al., 2015; Kuboyama et al., 2016; McClain, Sim, & Goldman, 2012; Meng et al., 2000; Tanga et al., 2019).

Pleiotrophin is expressed abundantly along axons in the developing and adult brain and spinal cord, as well as by some neurons and glia (Silos-Santiago et al., 1996), and is secreted by cortical neurons and their axons in response to demyelination (Kuboyama et al., 2015). Although to our knowledge this has not been reported for mouse, *Ptn* is expressed by human fetal oligodendrocyte precursor cells themselves (Sim et al., 2006; Sim et al., 2011), suggesting the possibility of autocrine as well as paracrine inhibition of Ptprz1 by pleiotrophin during oligodendrocyte development. In the olfactory system, *Ptn* expression was especially strong in inner ONL-OECs, and also present in olfactory mucosal mesenchyme at E13.0–13.5 and E16.5, and adjacent to the ONL at E16.5. Furthermore, *Ptn* expression was also present throughout the olfactory epithelium at early stages (E11.5), suggesting Ptn might also be expressed along olfactory axons, although olfactory epithelial expression was lower by E13.0–13.5 and almost absent by E16.5.

The pan-OEC-specific expression of *Ptprz1* and *Ptn* (plus local mesenchymal and early olfactory epithelial expression of *Ptn),* and the especially strong expression of *Ptn* in inner ONL-OECs, suggested the possibility that pleiotrophin-Ptprz1 interaction may be important for OEC differentiation, as well as for oligodendrocyte differentiation (Kuboyama et al., 2012; Kuboyama et al., 2015; Kuboyama et al., 2016; McClain et al., 2012; Meng et al., 2000; Tanga et al., 2019). Our analysis of *Ptprz1*-null (*Ptprz1^lacZ/lacZ^*) embryos revealed that expression of *Ptn* itself, plus the other pan-OEC markers *Plp1* and *Hey2*, and of the ONL-OEC-specific markers *Npy* and *Prss56* (though not *Nkd2*), was qualitatively increased compared to wild-type littermates, suggesting that Ptprz1 activity may indeed act as a brake on OEC differentiation, both in the mucosa and the ONL.

We assessed OEC function at E16.5 in the absence of *Ptprz1* by quantifying olfactory receptor neuron maturation as a proxy for olfactory axon targeting, and the proportion of GnRH neurons inside the forebrain (Barraud et al., 2013; Rich et al., 2018). We saw no effect on GnRH neurons, but olfactory receptor neuron maturation was significantly reduced (by about 25%) in Ptprz1-null embryos compared to wild-type littermates. This suggests that the disruption to normal OEC differentiation caused by the absence of Ptprz1 resulted in defects in olfactory axon targeting.

We undertook a similar analysis in *Ptn*-null embryos at the same stage, but saw no obvious differences compared with wild-type or heterozygous littermates. However, pleiotrophin could act redundantly in the olfactory system with another inhibitory ligand for Ptprz1 (Lamprianou et al., 2011), the membrane-tethered cell-adhesion molecule contactin (encoded by *Cntn1*). Contactin is presented on the surface of oligodendrocyte precursor cells themselves, promoting oligodendrocyte differentiation and myelination by inhibiting Ptprz1 phosphatase activity (Lamprianou et al., 2011). *Cntn1* was expressed by all OECs (and trigeminal Schwann cells), and also throughout the olfactory epithelium, suggesting that contactin, unlike pleiotrophin, could be expressed on olfactory axons.

Overall, these results and those described in the preceding two sections suggest that multiple genes involved in oligodendrocyte development also play roles in OEC development.

We also note that the cytokine interleukin-34 is an inhibitory ligand for Ptprz1 (Baghdadi et al., 2018; Chitu, Gokhan, Nandi, Mehler, & Stanley, 2016; Nandi et al., 2013). Given that OECs are the main phagocytic cells of the olfactory system (Nazareth et al., 2015; Su et al., 2013), it is interesting to speculate that Ptprz1 could also be involved in innate immune responses by OECs.

## 5 Summary and Perspective

OECs are a remarkable population of cells with multiple roles in the olfactory system during embryogenesis and throughout adult life, and considerable potential as a patient-specific cell-mediated therapy for CNS injury repair (Assinck et al., 2017; Ekberg et al., 2012; Ekberg & St John, 2014; Radtke & Kocsis, 2014; Roet & Verhaagen, 2014; Yao et al., 2018). Our unbiased laser microdissection and RNA-seq approach has identified 25 novel markers for developing OECs, including 15 that distinguish them from trigeminal Schwann cells, and 11 that distinguish embryonic mucosal OECs from ONL-OECs. Shared gene expression with neural crest-derived boundary cap cells suggests the possibility of a novel role for inner ONL-OECs. We have uncovered several parallels between OEC and oligodendrocyte development, including a role for the receptor tyrosine phosphatase Ptprz1. In addition to providing new insight into the diversification of neural crest-derived glial populations, and unexpected convergence between OECs and oligodendrocytes, this study provides a foundation for future translational work to identify and expand patient-specific OECs versus Schwann cells in culture, whether from olfactory mucosa biopsies, adult neural crest-derived stem cells such as those that persist in skin and hair follicles, or induced pluripotent stem cells (Liu & Cheung, 2016).

## Supplementary Material

File S1

File S2

File S3

File S4

File S5

File S6

Figures S1-15
