# Supplementary material for: Insights into olfactory ensheathing cell development from a laser-microdissection and transcriptome-profiling approach
Source: Glia. Author manuscript; Available in PMC 2020 Dec 1. (PMC7116175; doi:10.1002/glia.23870)
Supplement: Figures S1-15 [file EMS94522-supplement-Figures_S1_15.pdf]

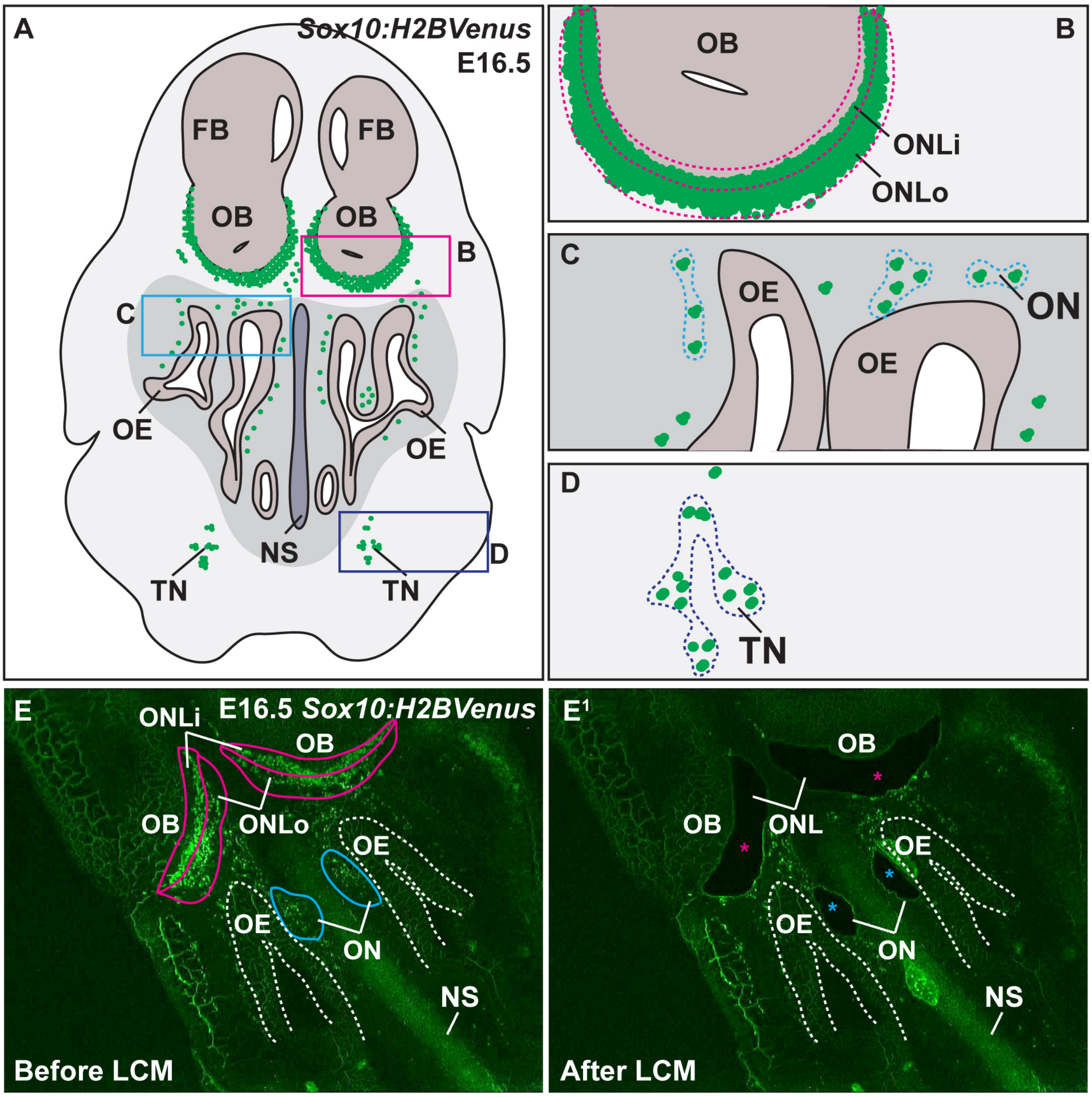

Figure S1. Laser-microdissection of embryonic olfactory and trigeminal nerve regions. (A) Schematic coronal section through the olfactory system of an E16.5 *Sox10:H2BVenus* BAC transgenic mouse embryo showing Sox10-positive OECs (green nuclei) associated with olfactory nerve fascicles near the olfactory epithelium (mucosal OECs) and throughout the ONL (ONL-OECs), which comprises an outer layer (ONLo) and inner layer (ONLi) at this stage. Sox10-positive Schwann cells (green nuclei) are also associated with trigeminal nerve fascicles near the olfactory system. **(B-D)** Higher power views of the boxed regions in A. **(E,E^1)** A coronal section through the olfactory system of an E16.5 *Sox10:H2BVenus* BAC transgenic mouse embryo, shown before (E) and after (E^1) laser-microdissection of pieces of outer and inner ONL (magenta outlines) and olfactory nerve (blue outlines) containing Sox10-positive OECs (green nuclei: native Venus fluorescence). Asterisks in E^1 indicate dissected regions. FB, forebrain; NS, nasal septum; OB, olfactory bulb; OE, olfactory epithelium; ON, olfactory nerve; ONL, olfactory nerve layer; ONLi, inner olfactory nerve layer; ONLo, outer olfactory nerve layer; TN, trigeminal nerve.

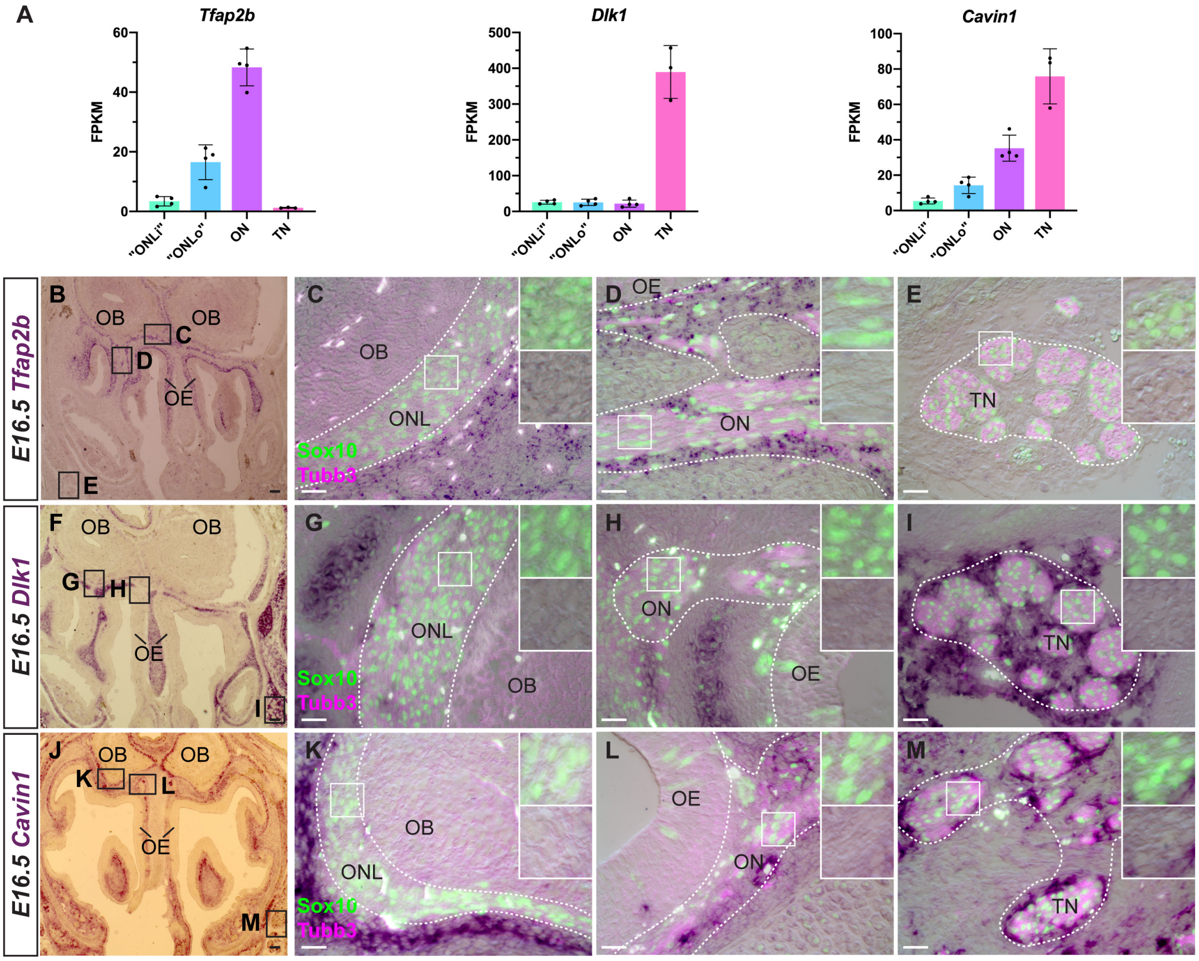

Figure S2. Examples of candidate genes expressed in mesenchyme adjacent to one or more laser-microdissected nerve regions, not glia. (A) Bar-charts showing mean expression values for *Tfap2b, Dlk1* and *Cavin1* across all transcriptomes at E16.5. Error-bars indicate standard deviation. **(B-M)** Coronal sections through the mouse olfactory system at E16.5, immunostained for Sox10 (green nuclei) to identify OECs and Schwann cells and for Tubb3 (magenta) to identify axons, following *in situ* hybridization for: (**B-E**) *Tfap2b*, predicted to be expressed by mucosal OECs and, more weakly, outer ONL-OECs (see A), but strongly expressed in mesenchyme adjacent to the ONL and olfactory nerve, but not adjacent to the trigeminal nerve (n=2 at E15.5-16.5); (**F-I**) *Dlk1*, predicted to be expressed by Schwann cells (see A), but strongly expressed in cartilage and in mesenchyme surrounding trigeminal, but not olfactory, nerve fascicles (n=2 at E15.5-16.5); (**J-M**) *Cavin1*, predicted to be expressed by outer ONL-OECs, mucosal OECs, and Schwann cells (see A), but strongly expressed in mesenchyme adjacent to the ONL and to both olfactory and trigeminal nerve fascicles (n=2 at E15.5-16.5). FPKM, fragments per kilobase of transcript per million mapped reads; OB, olfactory bulb; OE, olfactory epithelium; ON, olfactory nerve; ONL, olfactory nerve layer; ONLi, inner olfactory nerve layer; ONLo, outer olfactory nerve layer; TN, trigeminal nerve. Scale bars: B,F,J, 100 μm; C-E,G-I,K-M, 25 μm.

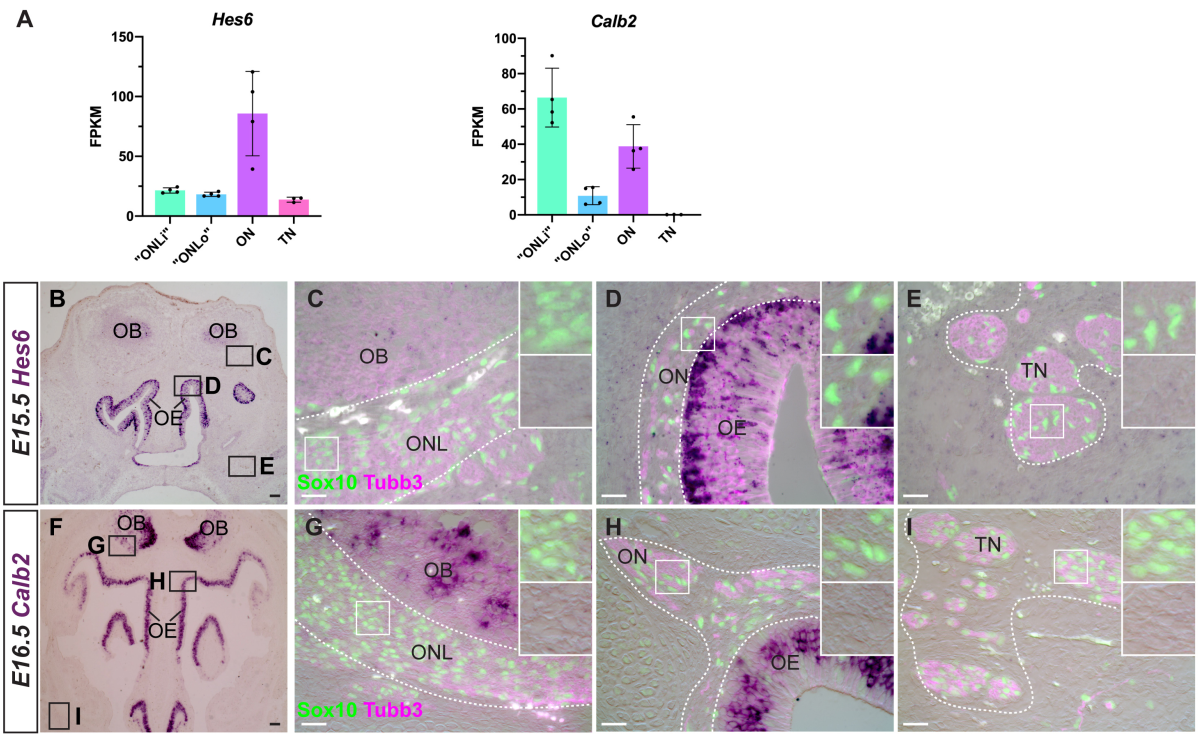

Figure S3. Examples of candidate genes expressed in the olfactory epithelium and/or olfactory bulb, not OECs. (A) Bar-charts showing mean expression values for *Hes6* and *Calb2* across all transcriptomes at E16.5. Error-bars indicate standard deviation. **(B-I)** Coronal sections through the mouse olfactory system at E15.5-16.5, immunostained for Sox10 (green nuclei) to identify OECs and Schwann cells and for Tubb3 (magenta) to identify axons, following *in situ* hybridization for: (**B-E**) *Hes6*, predicted to be expressed by mucosal OECs (see A), but strongly expressed in the olfactory epithelium (n=2); (**F-I**) *Calb2*, predicted to be expressed by inner ONL-OECs and mucosal OECs (see A), but expressed by cells in the olfactory bulb and olfactory epithelium (n=2). FPKM, fragments per kilobase of transcript per million mapped reads; OB, olfactory bulb; OE, olfactory epithelium; ON, olfactory nerve; ONL, olfactory nerve layer; ONLi, inner olfactory nerve layer; ONLo, outer olfactory nerve layer; TN, trigeminal nerve. Scale bars: B,F, 100 μm; C-E,G-I, 25 μm.

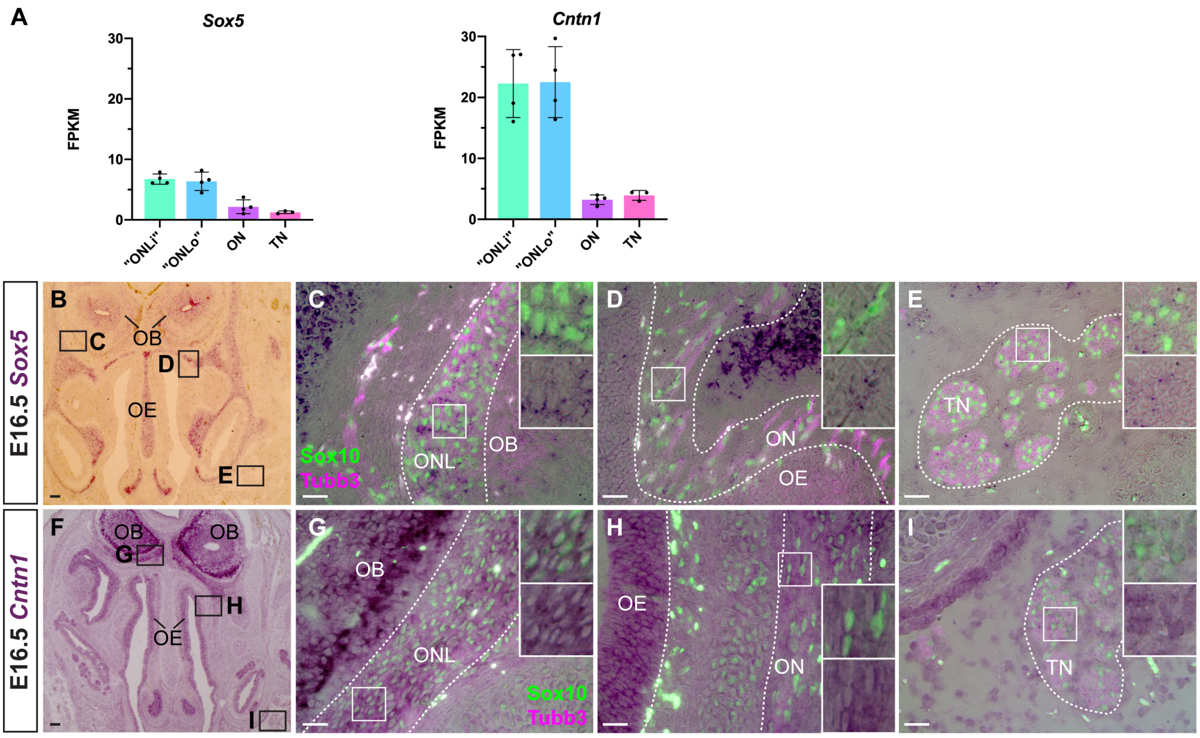

Figure S4. Two other genes involved in oligodendrocyte development are expressed by OECs at E16.5. (A) Bar-charts showing mean expression values across all transcriptomes at E16.5 for *Sox5* and *Cntn1*. Error-bars indicate standard deviation. **(B-I)** Coronal sections through the mouse olfactory system at E16.5, immunostained for Sox10 (green nuclei) to identify OECs and Schwann cells and for Tubb3 (magenta) to identify axons, following *in situ* hybridization for: (**B-E**) *Sox5*, which is expressed more strongly by ONL-OECs than by mucosal OECs or trigeminal Schwann cells (n=4); (**F-I**) *Cntn1*, which is expressed by ONL-OECs, mucosal OECs and trigeminal Schwann cells (n=3). FPKM, fragments per kilobase of transcript per million mapped reads; OB, olfactory bulb; OE, olfactory epithelium; ON, olfactory nerve; ONL, olfactory nerve layer; ONLi, inner olfactory nerve layer; ONLo, outer olfactory nerve layer; TN, trigeminal nerve. Scale bars: B,F, 100 μm; C-E,G-I, 25 μm.

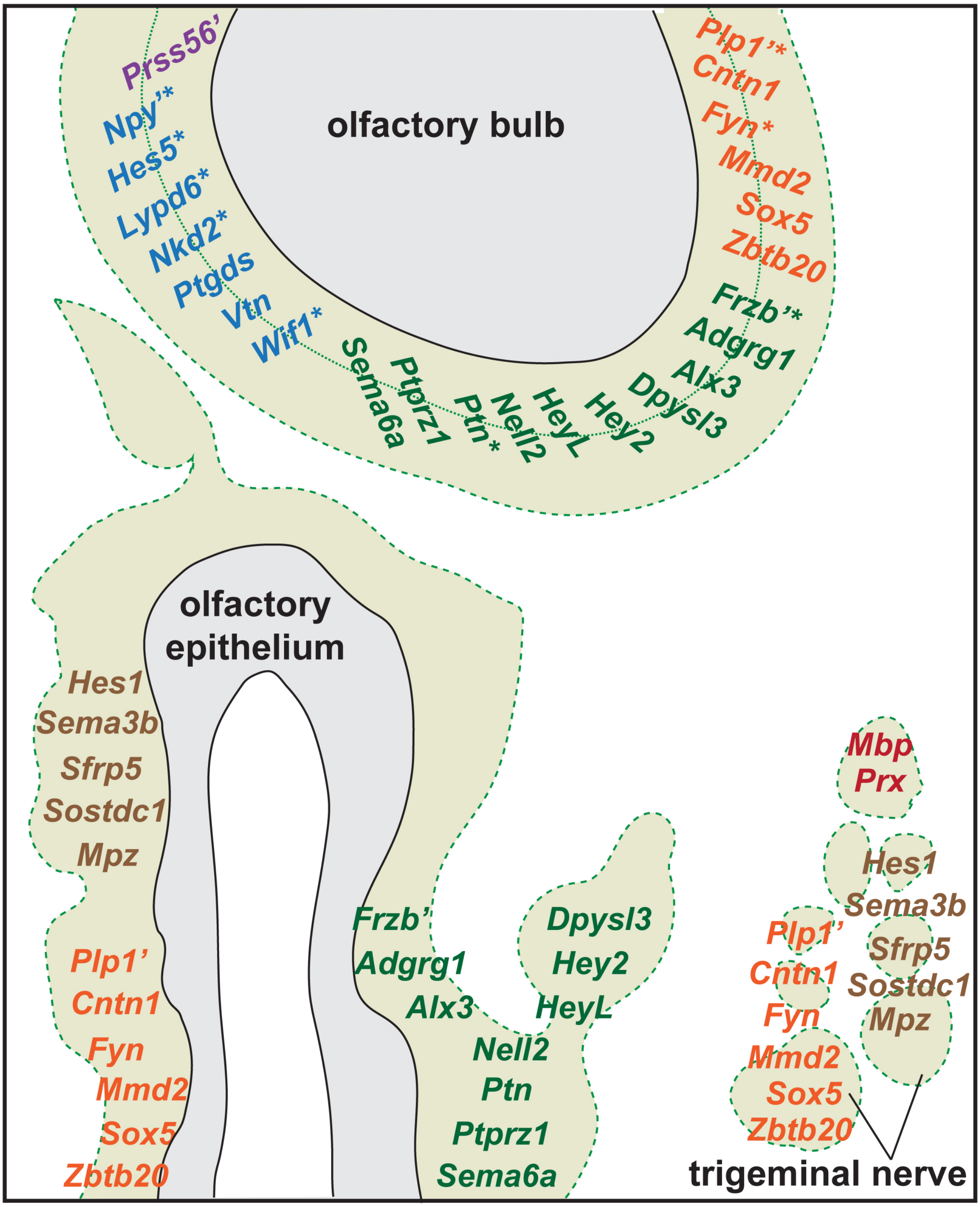

Figure S5. Graphical summary for candidate genes with validated expression in OECs and/or trigeminal Schwann cells at E16.5. Schematic coronal section through the olfactory system, showing the general location of ONL-OECs (around the olfactory bulb), mucosal OECs (associated with olfactory nerve fascicles near the olfactory epithelium), and trigeminal Schwann cells (on nearby trigeminal nerve branches). Genes are grouped by colour-coded expression category (in alphabetical order apart from previously reported markers for embryonic mouse OECs, which are listed first and marked with an apostrophe): purple, inner ONL-OECs only; blue, ONL-OECs only; green, ONL-OECs and mucosal OECs but not trigeminal Schwann cells; brown, mucosal OECs and trigeminal Schwann cells; orange, all OECs and trigeminal Schwann cells; red, trigeminal Schwann cells only. ONL-OEC genes with asterisks are enriched in the inner ONL.

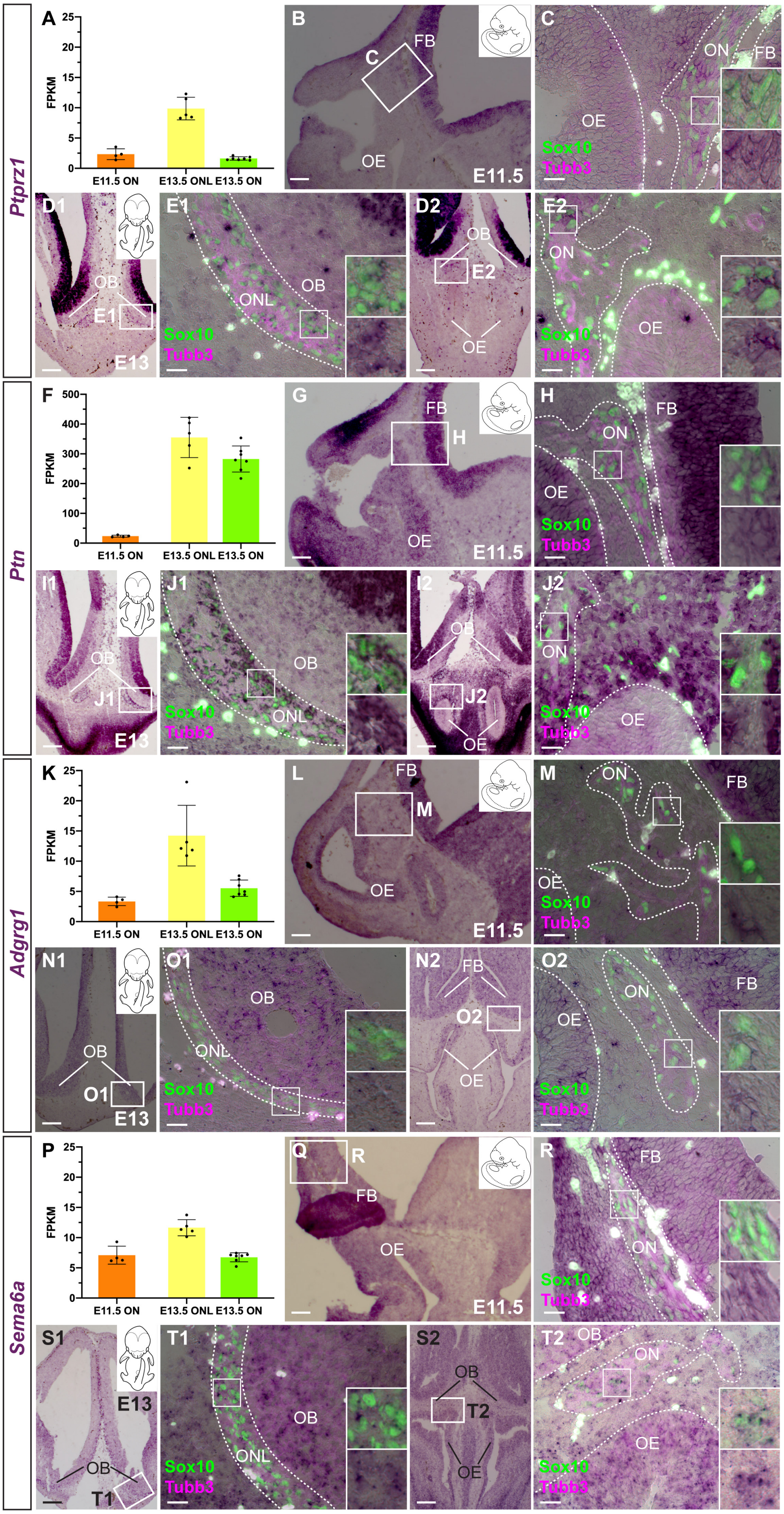
Ptprz1
Ptn
Adgrg1
Sema6a
FPKM
E11.5 ON
E13.5 ONL
E13.5 ON
FB
OE
ON
OB
ONL
Sox10
Tubb3
E11.5
E13

Figure S6. Mucosal OECs at E11.5 already express oligodendrocyte development-associated genes that are pan-OEC-specific at E16.5. Bar-charts showing mean expression values across all transcriptomes at E11.5 and E13.5 (error-bars indicate standard deviation), and parasagittal or coronal sections through the mouse olfactory system at E11.5 or E13.0 (orientation identified by redrawn Theiler stage schematics from the EMAP eMouse Atlas Project, http://www.emouseatlas.org; Richardson et al., 2014), immunostained for Sox10 (green nuclei) to identify OECs and Schwann cells and for Tubb3 (magenta) to identify axons, following *in situ* hybridization for: **(A-E2)** *Ptprz1* (n=2 at E11.5; n=3 at E13.0-13.5; panels D1-E2 show sections from the same embryo); **(F-J2)** *Ptn* (n=2 at E11.5; n=3 at E13.0-13.5; panels I1-J2 show sections from the same embryo); **(K-O2)** *Adgrg1* (n=2 at E11.5; n=3 at E13.0-13.5; panels N1-O2 show sections from the same embryo); **(P-T2)** *Sema6a* (n=2 at E11.5; n=3 at E13.0-13.5; panels S1-T2 show sections from the same embryo). All genes are expressed by mucosal OECs at E11.5 and by both ONL-OECs and mucosal OECs at E13.0. FB, forebrain; FPKM, fragments per kilobase of transcript per million mapped reads; OB, olfactory bulb; OE, olfactory epithelium; ON, olfactory nerve; ONL, olfactory nerve layer. Scale bars: B,D1,D2,G,I1,I2,L,N1,N2,Q,S1,S2, 100 μm; C,E1,E2,H,J1,J2,M,O1,O2,R,T1,T2, 25 μm.

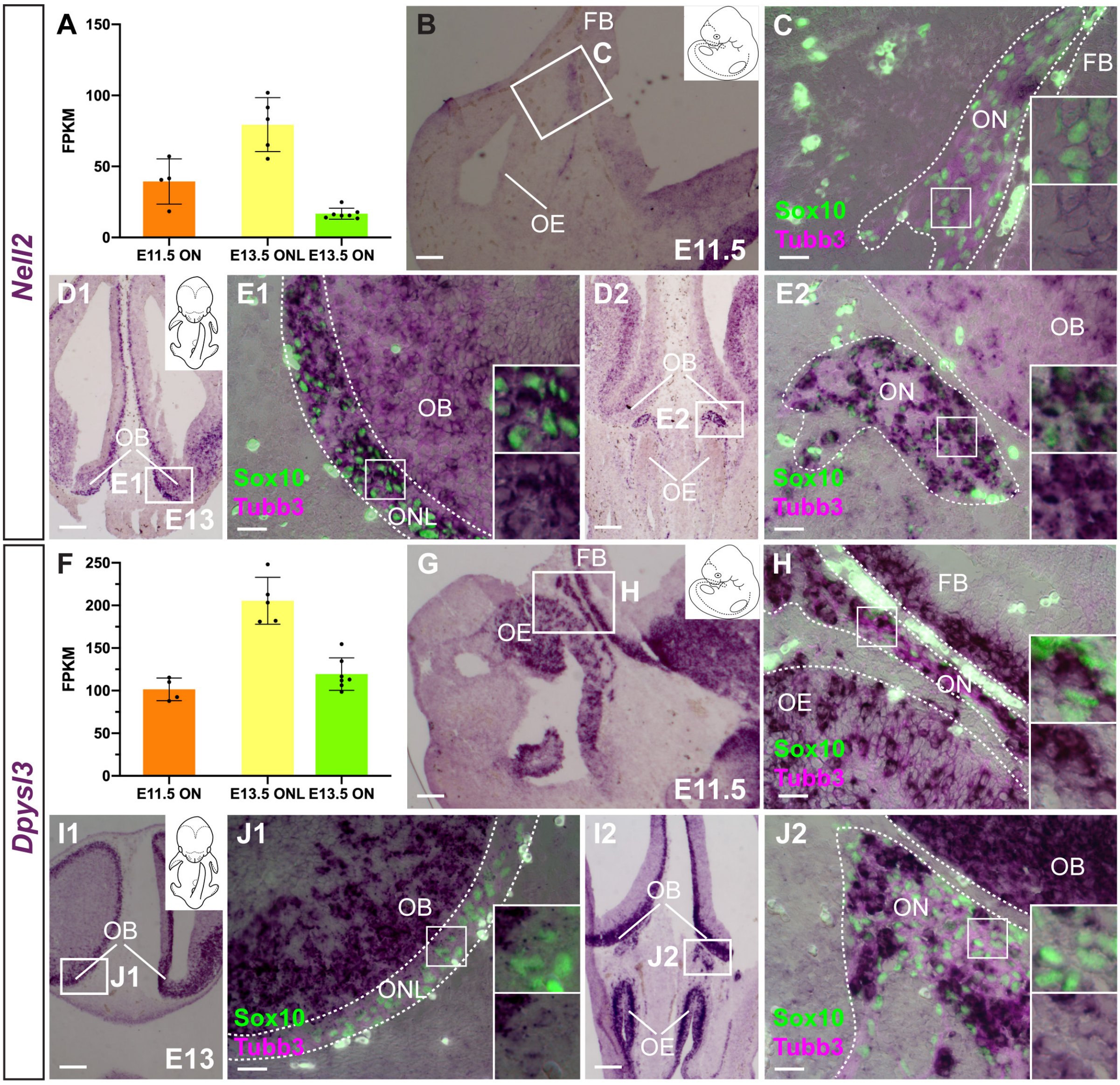

Figure S7. Mucosal OECs at E11.5 already express axon guidance/cell migration-associated genes that are pan-OEC-specific at E16.5: *Nell2*, *Dpysl3*. Bar-charts showing mean expression values across all transcriptomes at E11.5 and E13.5 (error-bars indicate standard deviation), and parasagittal or coronal sections through the mouse olfactory system at E11.5 or E13.0-13.5 (orientation identified by redrawn Theiler stage schematics from the EMAP eMouse Atlas Project, http://www.emouseatlas.org; Richardson et al., 2014), immunostained for Sox10 (green nuclei) to identify OECs and Schwann cells and for Tubb3 (magenta) to identify axons, following *in situ* hybridization for: **(A-E2)** *Nell2* (n=2 at E11.5; n=3 at E13.0-13.5; panels D1-E2 show sections from the same embryo); **(F-J2)** *Dpysl3* (*Crmp4*) (n=2 at E11.5; n=3 at E13.0-13.5; panels I1-J2 show sections from the same embryo). Both genes are expressed weakly by mucosal OECs at E11.5 and by both ONL-OECs and mucosal OECs at E13.0-13.5. *Dpysl3* is also expressed strongly by cells in the olfactory epithelium and at the edge of the rostral forebrain at E11.5 and the olfactory bulb at E13.0-13.5, and by clusters of Sox10-negative cells on the olfactory nerve at both stages, which are likely to be migrating neurons. FB, forebrain; FPKM, fragments per kilobase of transcript per million mapped reads; OB, olfactory bulb; OE, olfactory epithelium; ON, olfactory nerve; ONL, olfactory nerve layer. Scale bars: B,D1,D2,G,I1,I2, 100 µm; C,E1,E2,H,J1,J2, 25 µm.

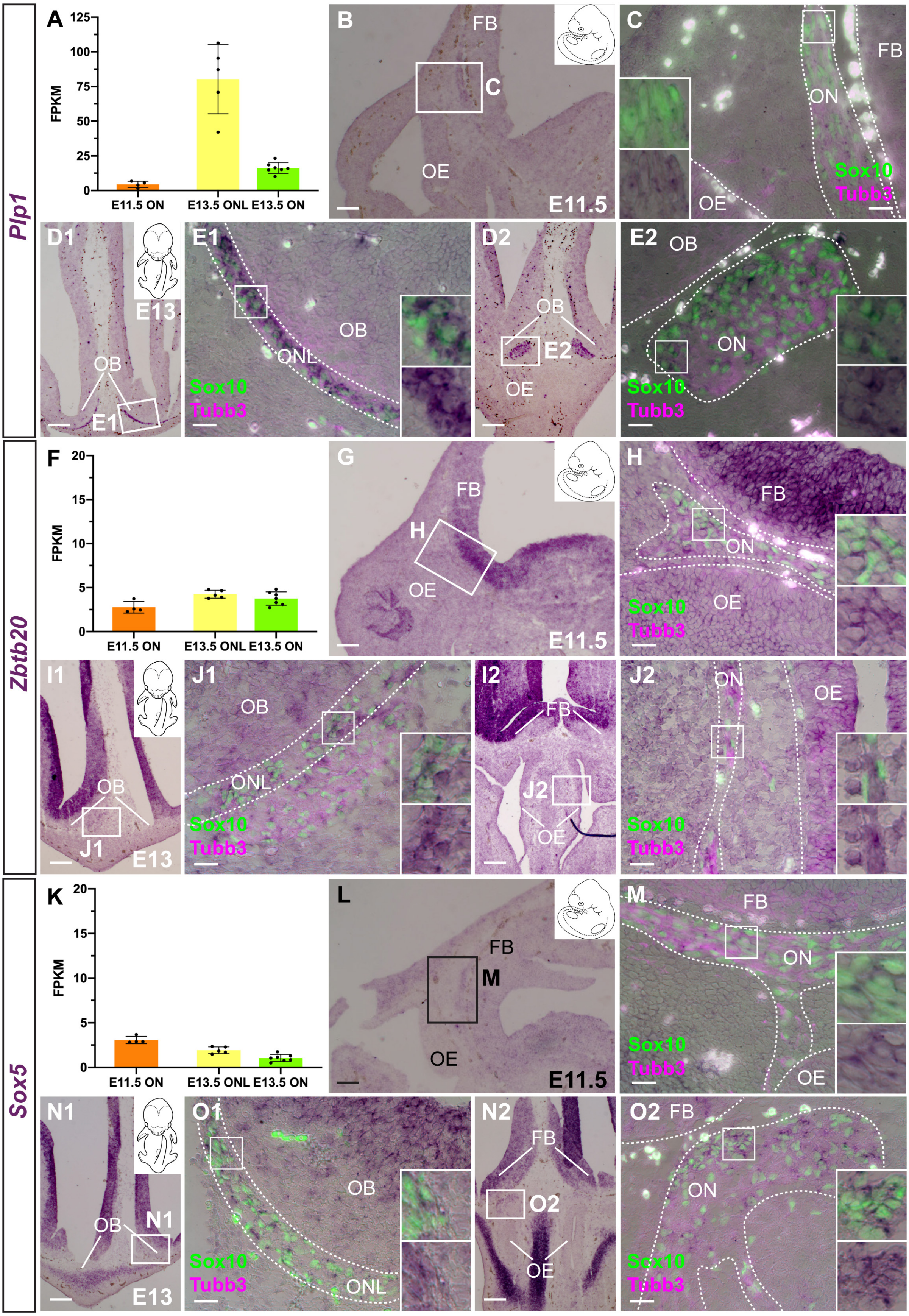

Figure S8. Mucosal OECs at E11.5 already express genes that are expressed by all OECs and trigeminal Schwann cells at E16.5: *Plp1*, *Zbtb20*, *Sox5*. Bar-charts showing

mean expression values across all transcriptomes at E11.5 and E13.5 (error-bars indicate standard deviation), and parasagittal or coronal sections through the mouse olfactory system at E11.5 or E13.0 (orientation identified by redrawn Theiler stage schematics from the EMAP eMouse Atlas Project, http://www.emouseatlas.org; Richardson et al., 2014), immunostained for Sox10 (green nuclei) to identify OECs and Schwann cells and for Tubb3 (magenta) to identify axons, following *in situ* hybridization for: **(A-E2)** *Plp1* (n=2 at E11.5; n=3 at E13.0-13.5; panels D1-E2 show sections from the same embryo); **(F-J2)** *Zbtb20* (n=2 at E11.5; n=3 at E13.0-13.5; panels I1-J2 show sections from the same embryo); **(K-O2)** *Sox5* (n=2 at E11.5; n=3 at E13.0-13.5; panels N1-O2 show sections from the same embryo). All three genes are expressed weakly by mucosal OECs at E11.5 and by both ONL-OECs and mucosal OECs at E13.0-13.5. FB, forebrain; FPKM, fragments per kilobase of transcript per million mapped reads; OB, olfactory bulb; OE, olfactory epithelium; ON, olfactory nerve; ONL, olfactory nerve layer. Scale bars: B,D1,D2,G,I1,I2,L,N1,N2, 100 μm; C,E1,E2,H,J1,J2,M,O1,O2, 25 μm.

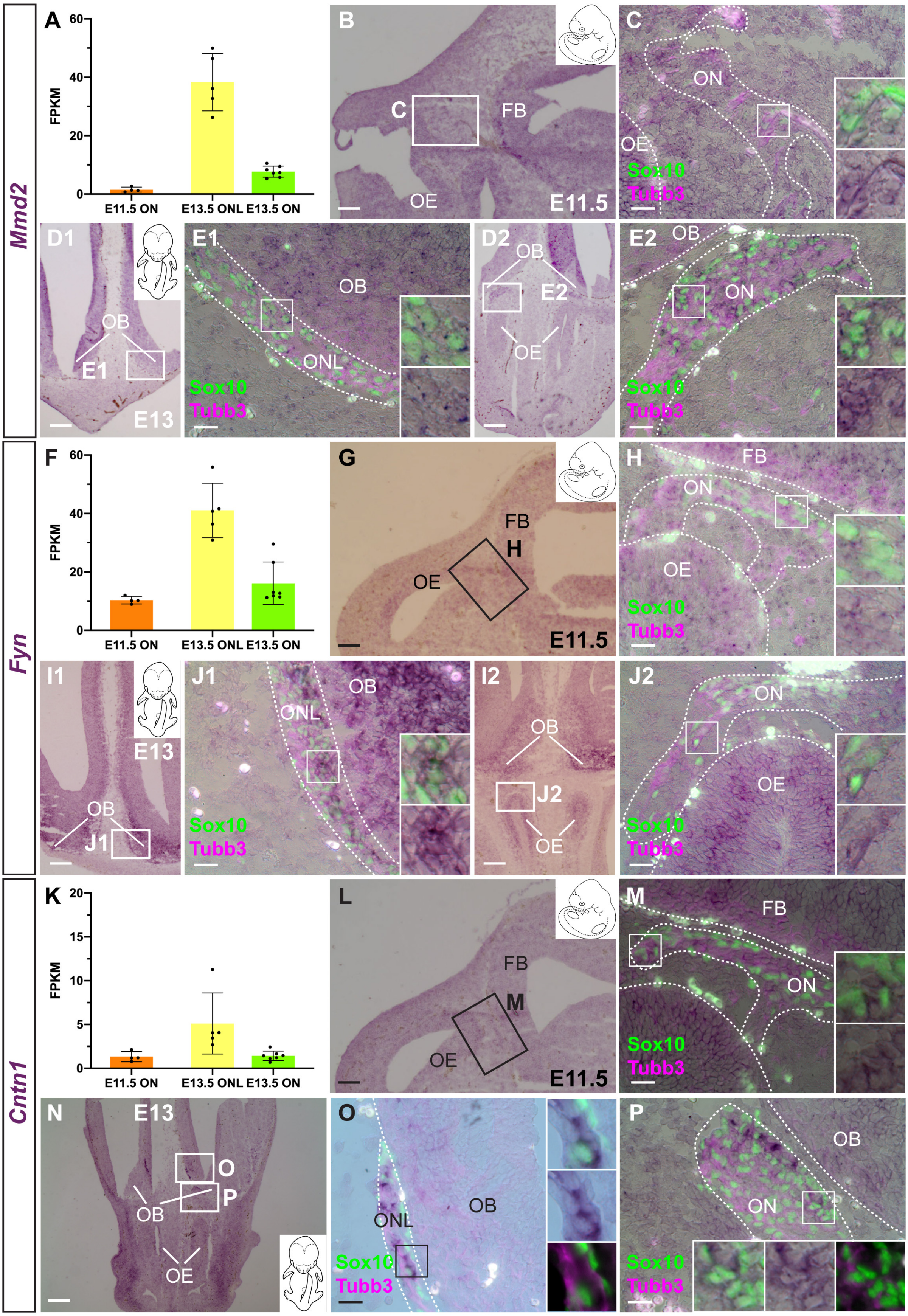

Figure S9. Mucosal OECs at E11.5 already express genes that are expressed by all OECs and trigeminal Schwann cells at E16.5: *Mmd2*, *Fyn*, *Cntn1*. Bar-charts showing

mean expression values across all transcriptomes at E11.5 and E13.5 (error-bars indicate standard deviation), and parasagittal or coronal sections through the mouse olfactory system at E11.5 or E13.0 (orientation identified by redrawn Theiler stage schematics from the EMAP eMouse Atlas Project, http://www.emouseatlas.org; Richardson et al., 2014), immunostained for Sox10 (green nuclei) to identify OECs and Schwann cells and for Tubb3 (magenta) to identify axons, following *in situ* hybridization for: **(A-E2)** *Mmd2* (n=2 at E11.5; n=3 at E13.0-13.5; panels D1-E2 show sections from the same embryo); **(F-J2)** *Fyn* (n=2 at E11.5; n=3 at E13.0-13.5; panels I1-J2 show sections from the same embryo); **(K-P)** *Cntn1* (n=2 at E11.5; n=3 at E13.0-13.5). All three genes are expressed weakly by mucosal OECs at E11.5 and by both ONL-OECs and mucosal OECs at E13.0-13.5. *Cntn1* is also strongly expressed by some Sox10-negative cells on the olfactory nerve at E13.0-13.5, which could be migrating neurons. FB, forebrain; FPKM, fragments per kilobase of transcript per million mapped reads; OB, olfactory bulb; OE, olfactory epithelium; ON, olfactory nerve; ONL, olfactory nerve layer. Scale bars: B,D1,D2,G,I1,I2,L,N, 100 µm; C,E1,E2,H,J1,J2,M,O,P, 25 µm.

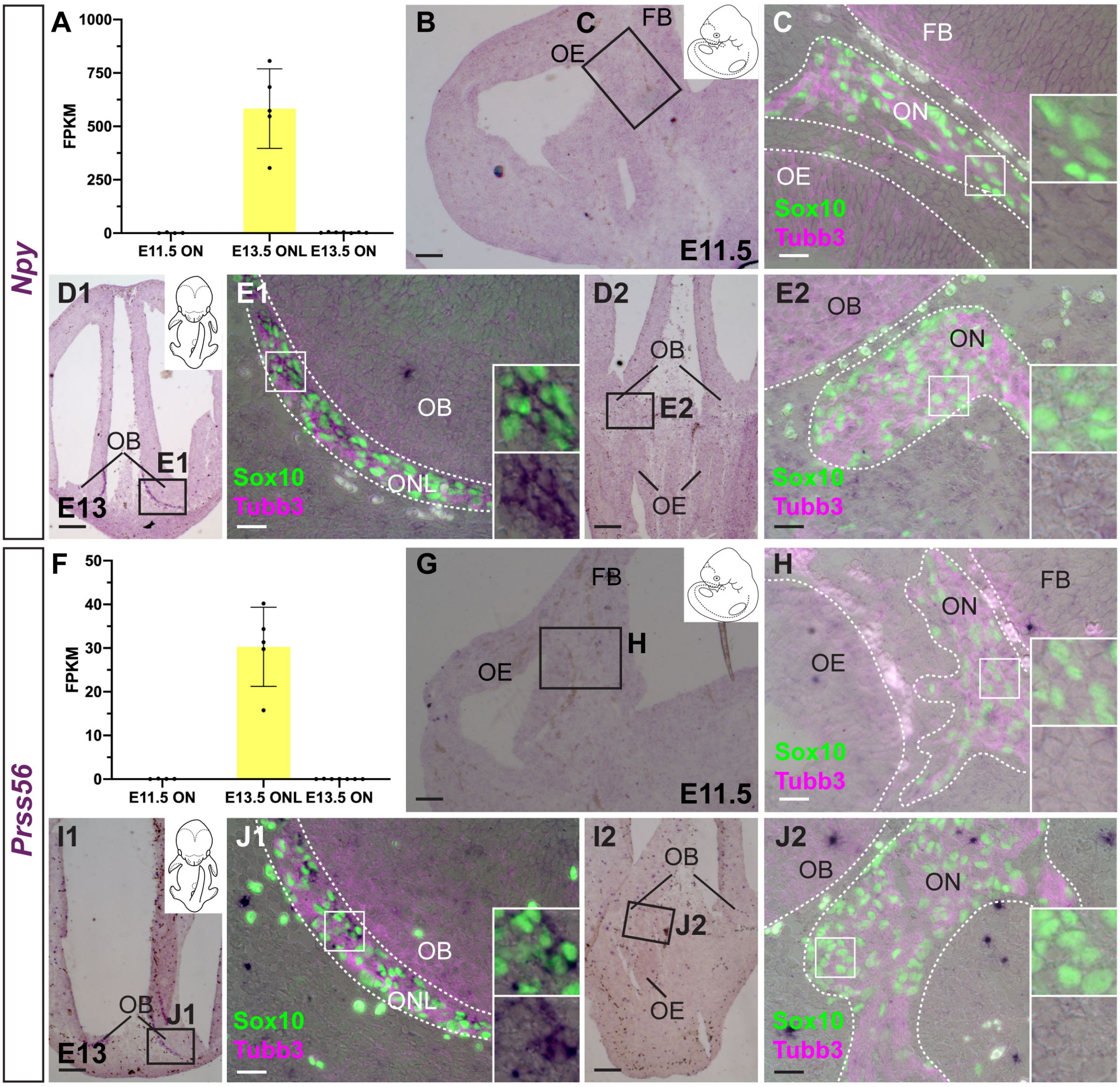

Figure S10. Mucosal OECs at E11.5 and E13.0-13.5 do not express genes that are ONL-OEC-specific at E16.5: *Npy*, *Prss56*. Bar-charts showing mean expression values across all transcriptomes at E11.5 and E13.5 (error-bars indicate standard deviation), and parasagittal or coronal sections through the mouse olfactory system at E11.5 or E13.0-13.5 (orientation identified by redrawn Theiler stage schematics from the EMAP eMouse Atlas Project, http://www.emouseatlas.org; Richardson et al., 2014), immunostained for Sox10 (green nuclei) to identify OECs and Schwann cells and for Tubb3 (magenta) to identify axons, following *in situ* hybridization for: **(A-E2)** *Npy* (n=2 at E11.5; n=7 at E13.0-13.5; panels D1-E2 show sections from the same embryo); **(F-J2)** *Prss56* (n=2 at E11.5; n=3 at E13.0-13.5; panels I1-J2 show sections from the same embryo). No expression is seen in mucosal OECs at any stage; both genes are expressed by ONL-OECs at E13.0-13.5 (though *Prss56* expression is restricted to ONL-OECs closest to the olfactory bulb). FB, forebrain; FPKM, fragments per kilobase of transcript per million mapped reads; OB, olfactory bulb; OE, olfactory epithelium; ON, olfactory nerve; ONL, olfactory nerve layer. Scale bars: B,D1,D2,G,I1,I2, 100 μm; C,E1,E2,H,J1,J2, 25 μm.

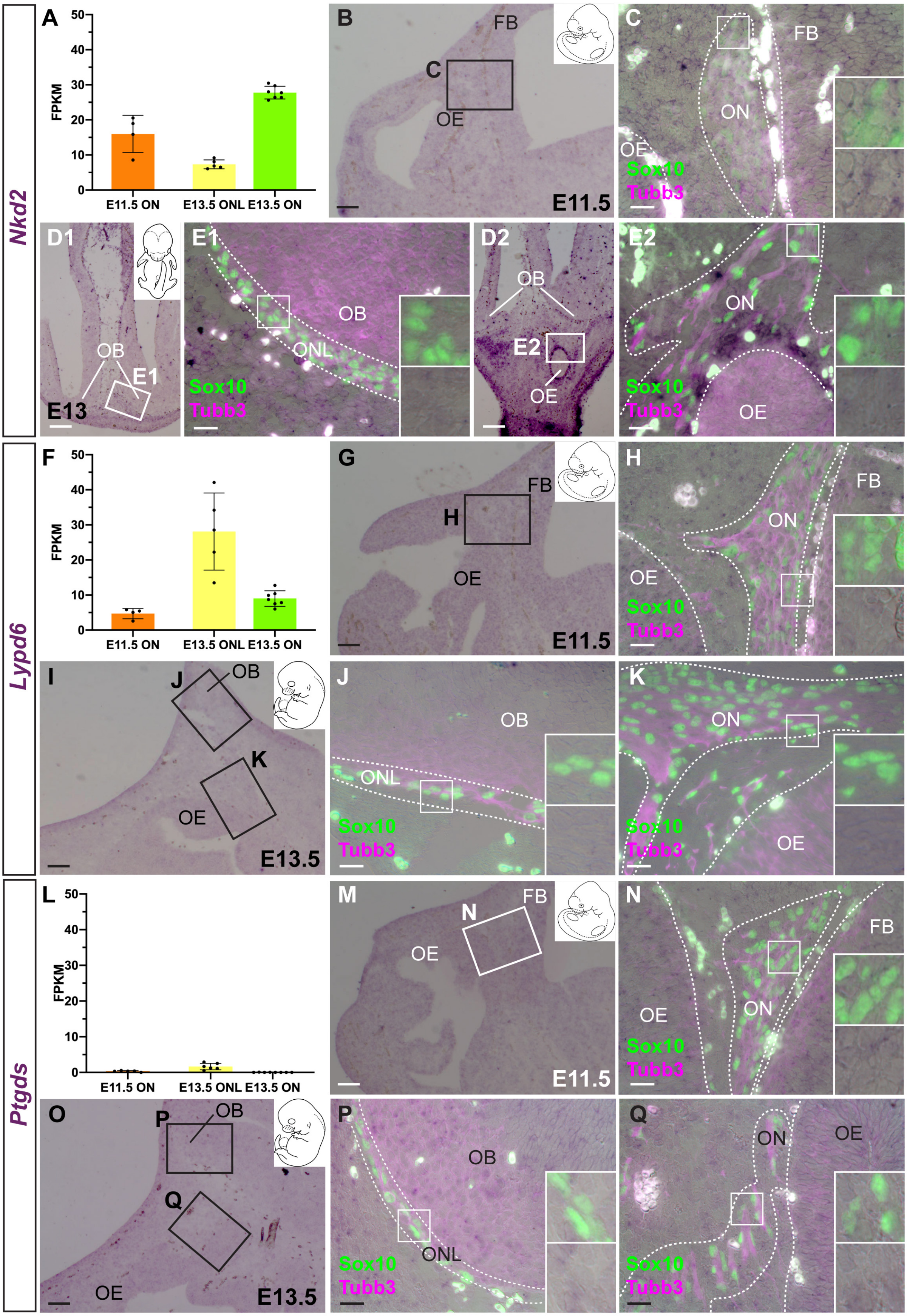

Figure S11. Mucosal OECs at E11.5 and E13.0-13.5 do not express genes that are ONL-OEC-specific at E16.5: *Nkd2*, *Lypd6*, *Ptgds*. Bar-charts showing mean expression

values across all transcriptomes at E11.5 and E13.5 (error-bars indicate standard deviation), and parasagittal or coronal sections through the mouse olfactory system at E11.5 or E13.0-13.5 (orientation identified by redrawn Theiler stage schematics from the EMAP eMouse Atlas Project, http://www.emouseatlas.org; Richardson et al., 2014), immunostained for Sox10 (green nuclei) to identify OECs and Schwann cells and for Tubb3 (magenta) to identify axons, following *in situ* hybridization for: **(A-E2)** *Nkd2* (n=2 at E11.5; n=3 at E13.0-13.5; panels D1-E2 show sections from the same embryo); **(F-K)** *Lypd6* (n=2 at E11.5; n=3 at E13.0-13.5); **(L-Q)** *Ptgds* (n=2 at E11.5; n=3 at E13.0-13.5). These genes are not expressed by mucosal or ONL-OECs at either E11.5 or E13.0-13.5. FB, forebrain; FPKM, fragments per kilobase of transcript per million mapped reads; OB, olfactory bulb; OE, olfactory epithelium; ON, olfactory nerve; ONL, olfactory nerve layer. Scale bars: B,D1,D2,G,I,M,O, 100 μm; C,E1,E2,H,J,K,N,P,Q, 25 μm.

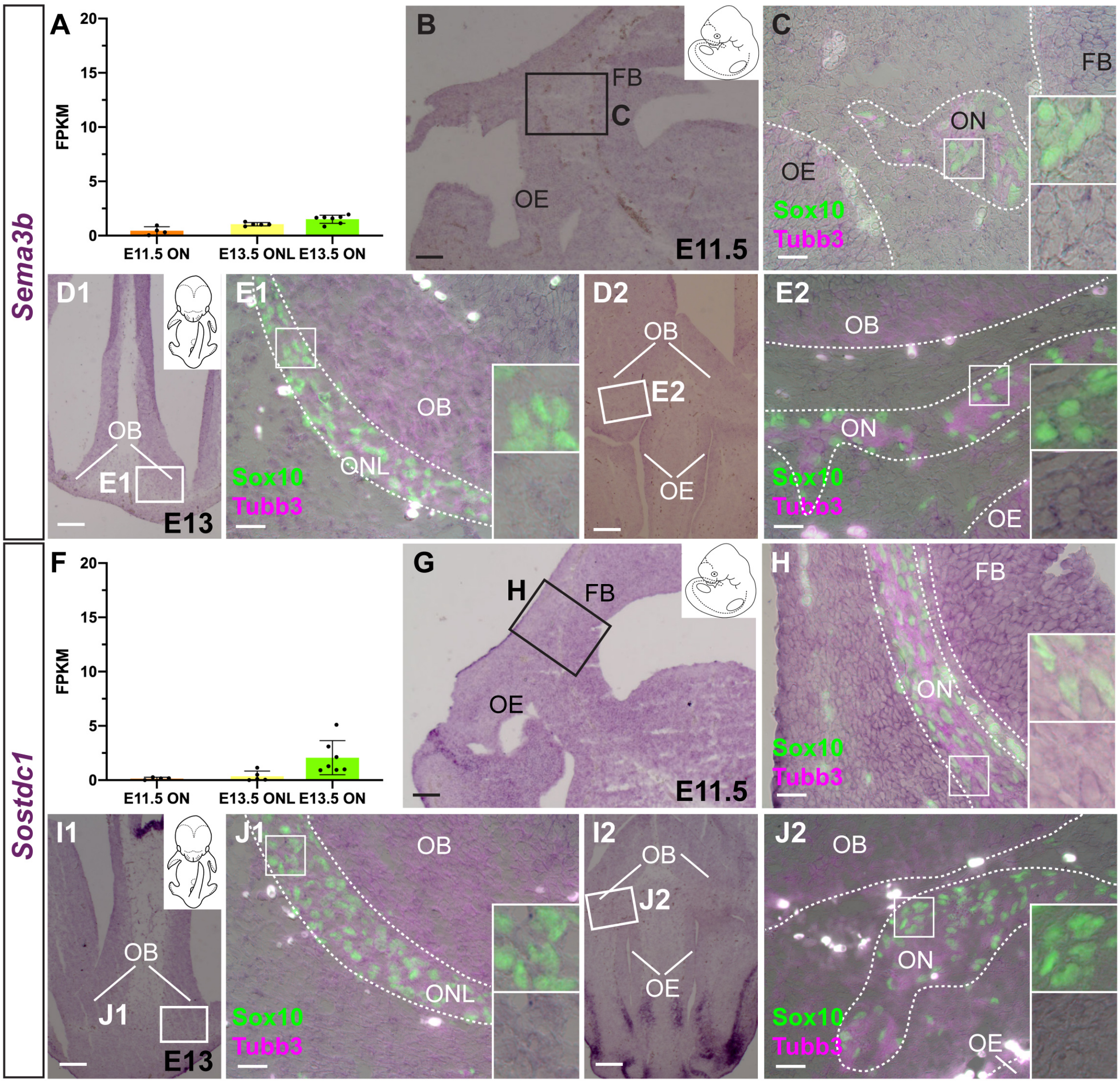

Figure S12. Some genes expressed by mucosal OECs but not ONL-OECs at E16.5 are expressed later than E11.5. Bar-charts showing mean expression values across all transcriptomes at E11.5 and E13.5 (error-bars indicate standard deviation), and parasagittal or coronal sections through the mouse olfactory system at E11.5 or E13.0 (orientation identified by redrawn Theiler stage schematics from the EMAP eMouse Atlas Project, http://www.emouseatlas.org; Richardson et al., 2014), immunostained for Sox10 (green nuclei) to identify OECs and Schwann cells and for Tubb3 (magenta) to identify axons, following *in situ* hybridization for: **(A-E2)** *Sema3b* (n=2 at E11.5; n=3 at E13.0-13.5), which is not expressed by mucosal OECs at E11.5 but is expressed by mucosal but not ONL-OECs at E13.0-13.5; **(F-J2)** *Sostdc1* (*Wise*) (n=2 at E11.5; n=3 at E13.0-13.5; panels I1-J2 show sections from the same embryo), which is not expressed by OECs at either stage. FB, forebrain; FPKM, fragments per kilobase of transcript per million mapped reads; OB, olfactory bulb; OE, olfactory epithelium; ON, olfactory nerve; ONL, olfactory nerve layer. Scale bars: B,D1,D2,G,I1,I2, 100 μm; C,E1,E2,H,J1,J2, 25 μm.

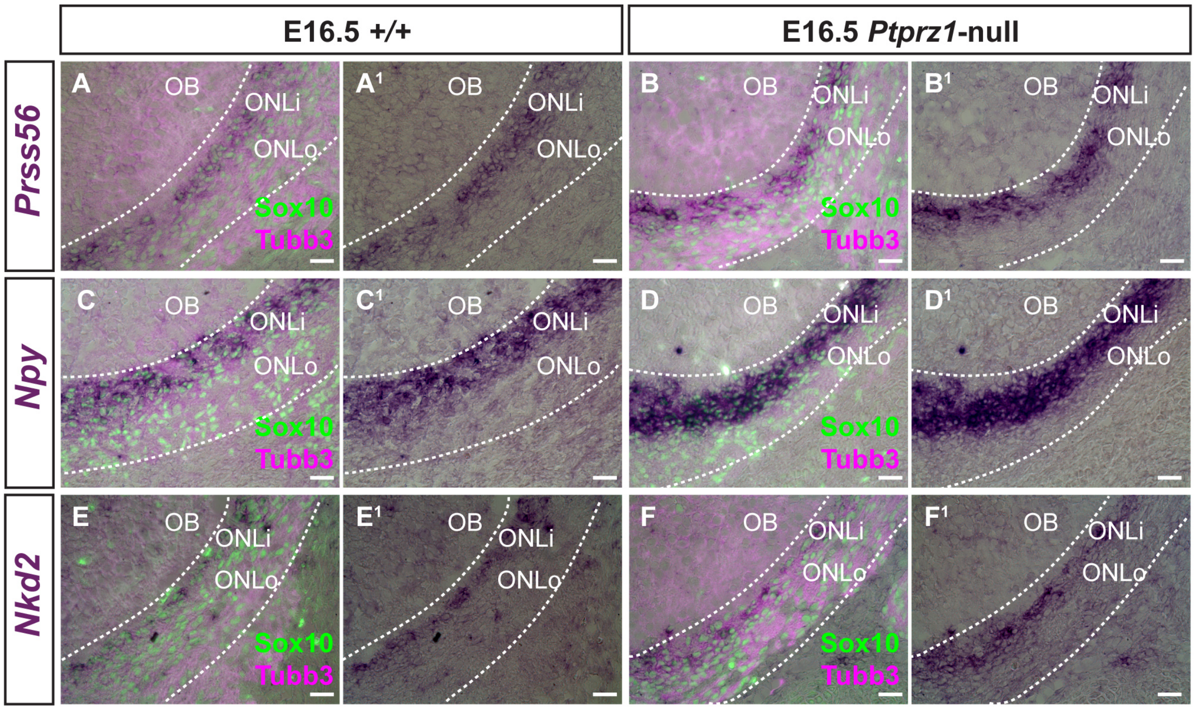

Figure S13. *Ptprz1* deletion leads to increased expression of the ONL-OEC specific markers *Prss56* and *Npy*, but not *Nkd2*. Coronal sections through the olfactory system of wild-type (n=4 from one litter for each gene) or *Ptprz1*-null (*Ptprz1*$^{lacZ/lacZ}$) littermates at E16.5 (n=3 from one litter for each gene), immunostained for Sox10 (green nuclei) to identify OECs and for Tubb3 (magenta) to identify axons, following *in situ* hybridization for: (**A-B^1**) the inner ONL-OEC-specific marker *Prss56*, whose expression is stronger in *Ptprz1*-null inner ONL-OECs than in wild-type embryos; (**C-D^1**) the ONL-OEC-specific (inner ONL-enriched) marker *Npy*, whose expression is stronger in *Ptprz1*-null ONL-OECs than in wild-type embryos; (**E-F^1**) the ONL-OEC-specific (inner ONL-enriched) marker *Nkd2*, whose expression does not seem to differ between *Ptprz1*-null and wild-type embryos. The ISH colour reaction was stopped at the same time for each pair of sections shown from the different genotypes. OB, olfactory bulb; OE, olfactory epithelium; ON, olfactory nerve; ONL, olfactory nerve layer; ONLi, inner olfactory nerve layer; ONLo, outer olfactory nerve layer; TN, trigeminal nerve. Scale bar: 25 µm.

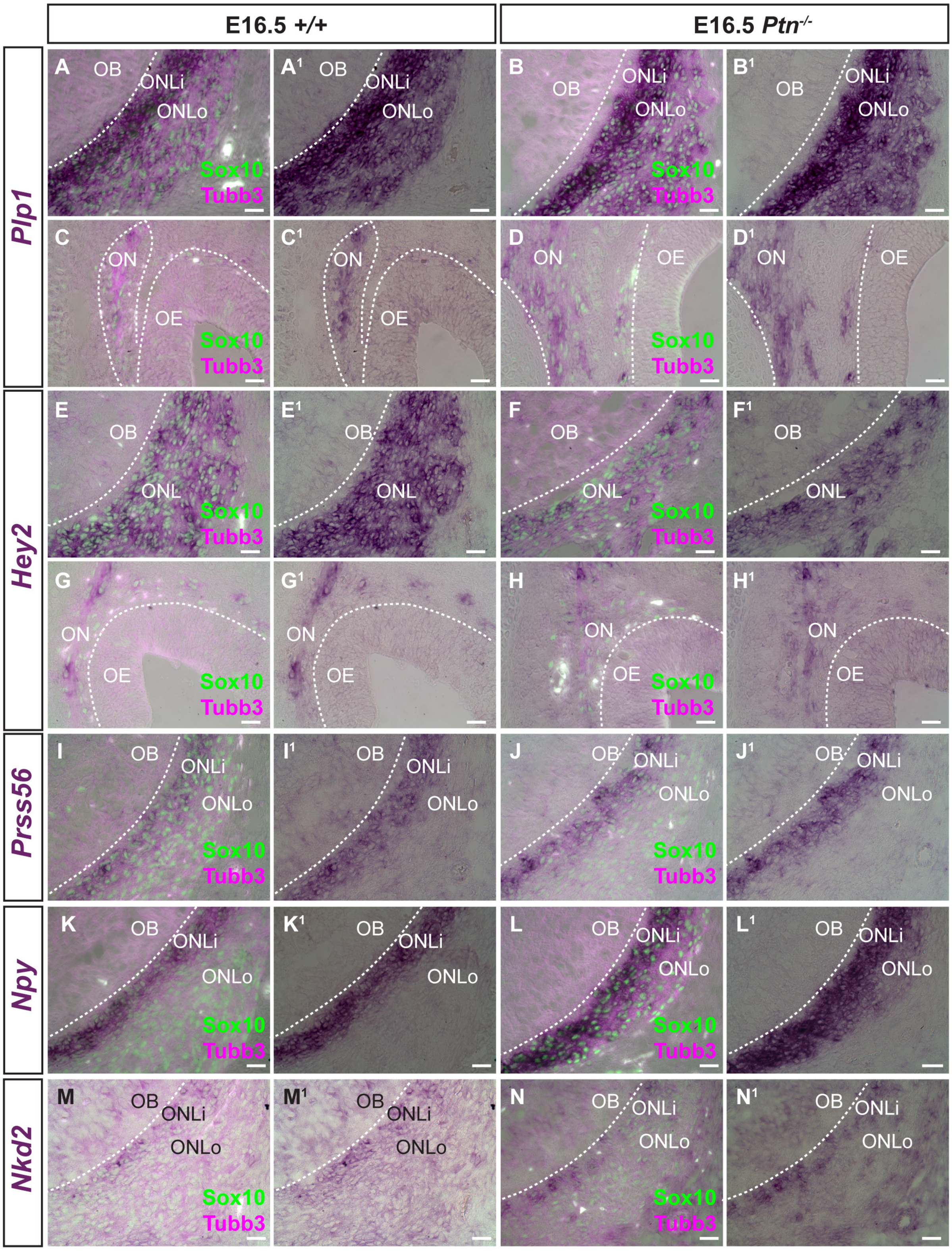

Figure S14. *Ptn* deletion has no obvious effect on OEC differentiation. Coronal sections through the olfactory system of wild-type E16.5 embryos (n=3 from one litter for each gene) or *Ptn*-null littermates (n=4 for each gene), immunostained for Sox10 (green nuclei) to identify OECs and for Tubb3 (magenta) to identify axons, following *in situ* hybridization for: (**A-D^1**) the pan-OEC (inner ONL-enriched) gene *Plp1*; (**E-H^1**) the pan-OEC-specific gene *Hey2*; (**I-J^1**) the inner ONL-OEC-specific gene *Prss56*; (**K-L^1**) the ONL-OEC-specific (inner ONL-enriched) gene *Npy*; (**M-N^1**) the ONL-OEC-specific (inner ONL-enriched) gene *Nkd2*.

No differences in OEC gene expression were seen between wild-type and *Ptn*-null littermates. The ISH colour reaction was stopped at the same time for each pair of sections shown from the different genotypes. OB, olfactory bulb; OE, olfactory epithelium; ON, olfactory nerve; ONL, olfactory nerve layer; ONLi, inner olfactory nerve layer; ONLo, outer olfactory nerve layer. Scale bar: 25 μm.

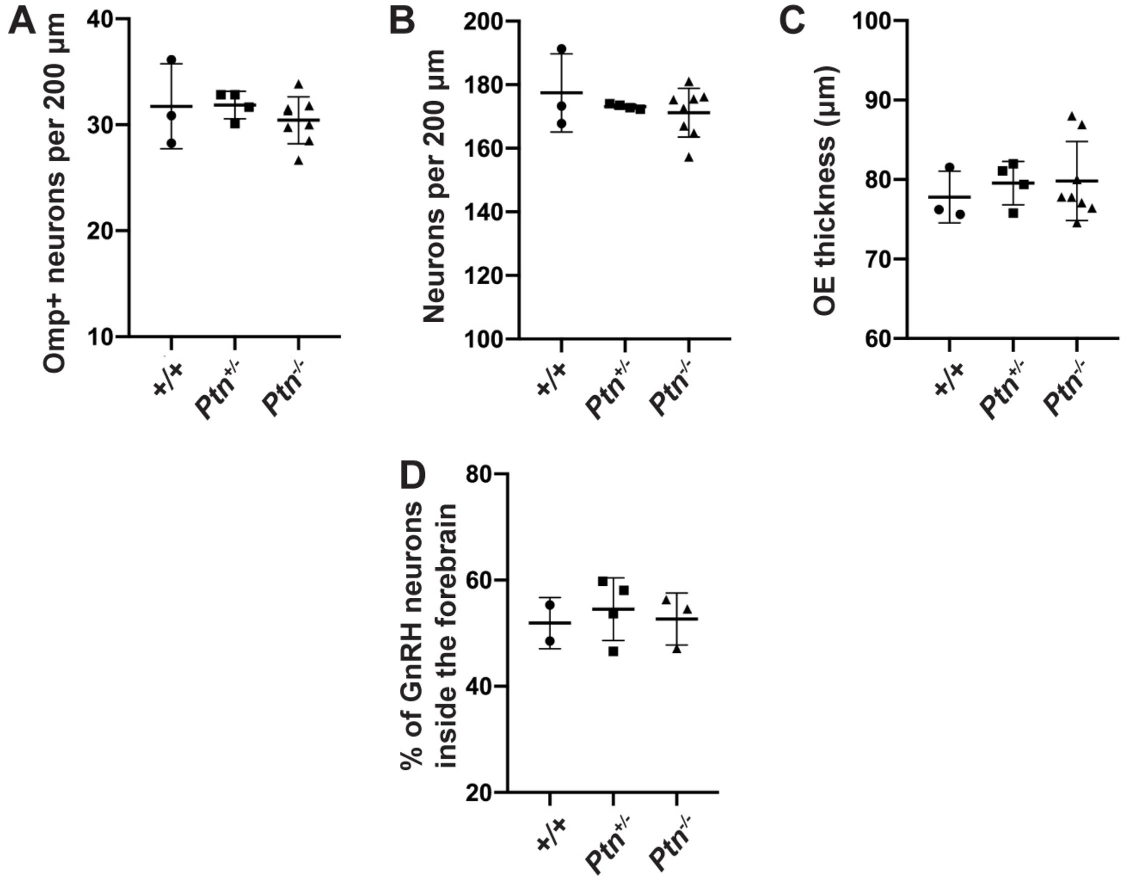

Figure S15. ***Ptn*** **deletion does not disrupt olfactory receptor neuron maturation or GnRH neuron entry into the forebrain.** Bars on scatter plots show mean and standard deviation; data collected from wild-type, $Ptn^{+/-}$ heterozygotes and *Ptn*-null littermates from two litters at E16.5. (**A**) Scatter plot showing the mean number per embryo of Omp-positive (mature) neurons per 200 µm of dorsal olfactory epithelium (selected on both left and right sides of at least 3 coronal sections per embryo) for wild-type embryos (mean 31.8 ± 4.0; n=3), $Ptn^{+/-}$ heterozygotes (mean 31.9 ± 1.3; n=4) and *Ptn*-null embryos (mean 30.4 ± 2.2; n=8). There is no significant difference between these groups (p=0.56; one-way analysis of variance with Dunnett's multiple comparison test). (**B**) Scatter plot showing the mean number per embryo of neurons per 200 µm of olfactory epithelium (at least 6 such regions analysed per embryo) for wild-type embryos (mean 177.4 ± 12.3; n=3), $Ptn^{+/-}$ heterozygotes (mean 173.2 ± 0.75; n=4) and *Ptn*-null embryos (mean 171.2 ± 7.6; n=8). There is no significant difference between these groups (p=0.51; one-way analysis of variance with Dunnett's multiple comparison test). (**C**) Scatter plot showing the mean thickness per embryo of the olfactory epithelium (3 measurements made per 200 µm region of olfactory epithelium counted for neurons) for wild-type embryos (mean 77.8 ± 3.3 µm; n=3), $Ptn^{+/-}$ heterozygotes (mean 79.6 ± 2.7 µm; n=4) and *Ptn*-null embryos (mean 79.8 ± 5.0 µm; n=8). There is no significant difference between these groups (p=0.78; one-way analysis of variance with Dunnett's multiple comparison test). (**D**) Scatter plot showing the percentage of GnRH neurons (at least 87 counted per embryo) found inside the brain for wild-type embryos (51.9 ± 4.8%; n=2), $Ptn^{+/-}$ heterozygotes (mean 54.5 ± 5.9%; n=4) and *Ptn*-null embryos (mean 52.7 ± 4.9%; n=3). There is no significant difference between these groups (p=0.92; Kruskal-Wallis test followed by Dunn's multiple comparisons test).
